# A network meta-analysis of psychological interventions for children and adolescents after natural and man-made disasters

**DOI:** 10.1186/s12888-024-05924-8

**Published:** 2024-06-25

**Authors:** Yu Xie, Xiang Zhu, Lijun Wang, Zhendong Wan, Jiyu Yang, Chen Su, Shuyu Duan, Chenxi Xu, Binbin Kan

**Affiliations:** 1 School of Educational Science, Anhui Normal University, Wuhu, China; 2 School of Computer and Information, Anhui Normal University, Wuhu, China; 3 Office of Scientific Research, XuanCheng Vocational & Technical College, Xuancheng, China; 4 School of Educational Science, Jiangsu Second Normal University, Nanjing, China

**Keywords:** Network meta-analysis, Post-traumatic stress disorder, Depression, Intervention, Disaster

## Abstract

**Introduction:**

Children and adolescents, after natural and man-made disasters, often exhibit various psychological, emotional, and behavioral issues, showing a range of clinical symptoms related to post-traumatic stress disorder (PTSD) and depression. This review used a network meta-analysis (NMA) approach to compare and rank psychological interventions for PTSD and depression in children and adolescents after exposure to natural and man-made disasters.

**Methods:**

Randomized studies of psychosocial interventions for PTSD and depression in children and adolescents exposed to natural and man-made disasters were identified. PTSD and depression symptoms at postintervention and 1–12 month follow-up are the outcomes. The standardized mean differences (SMDs) between pairs of interventions at postintervention and follow-up were pooled. Mean effect sizes with 95% credible intervals (CI) were calculated, and the ranking probabilities for all interventions were estimated using the surface under the cumulative ranking curve. Study quality was assessed with version 2 of the Cochrane risk-of-bias tool for randomized trials (RoB 2).

**Results:**

In total, 26 studies with 4331 participants were included in this NMA. Eye movement desensitization and reprocessing therapy (EMDR) (SMD = − 0.67; 95% CI − 1.17 to − 0.17), exposure therapy (ET) (SMD = − 0.66; 95% CI − 1.11 to − 0.22), and cognitive behavioral therapy (CBT) (SMD = − 0.62; 95% CI − 0.90 to − 0.34) were significantly more effective for PTSD at postintervention than inactive intervention. EMDR (SMD = − 0.72; 95% CI − 1.11 to − 0.33) and ET (SMD = − 0.62; 95% CI − 0.97 to − 0.27) were associated with a higher reduction in PTSD symptoms at follow-up than inactive intervention. EMDR (SMD = − 0.40; 95% CI − 0.78 to − 0.03) and play therapy (PT) (SMD = − 0.37; 95% CI − 0.62 to − 0.12) were significantly more effective for depression at postintervention than inactive intervention. For all psychological interventions in reducing depression symptoms at follow-up compared with inactive intervention, the differences were not significant.

**Conclusion:**

EMDR appears to be most effective in reducing PTSD and depression in children and adolescents exposed to natural and man-made disasters. In addition, ET and CBT are potentially effective in reducing PTSD symptoms at postintervention, while PT is beneficial in managing depression symptoms at the treatment endpoint.

**Supplementary Information:**

The online version contains supplementary material available at 10.1186/s12888-024-05924-8.

## Introduction

Natural and man-made disasters, such as earthquakes, cyclones, tsunamis, floods, war, and terrorist attacks, are mostly unpredictable, which often lead to severe consequences, such as environmental loss, displacement of the family, property damage, physical injury, and even death of a loved one [1]. Disasters and social and economic losses leave victims in despair, fear, shock, and maladjustment [2]. The victims of these traumatic experiences often display psychological, emotional, and behavioral issues. They show many clinical symptoms of post-traumatic stress disorder (PTSD) and depression. In particular, children and adolescents are particularly vulnerable after exposure to disasters because they lack psychological preparedness for disasters and coping skills for traumatic experiences [3]. A cross-sectional study found that the prevalence of PTSD and depression in adolescents six months after exposure to an earthquake was 58.3% and 16.8% [4]. Natural and man-made disasters might negatively impact mental health outcomes in childhood and influence psychological symptoms into adulthood. Experiencing a disaster at a young age is a risk factor for adult mental health disorders [5]. Therefore, psychological interventions for children and adolescents should be given a greater emphasis besides socioeconomic support following a disaster.

Given the psychological impact of natural and man-made disasters, a variety of psychological interventions, including cognitive behavioral therapy (CBT), eye movement desensitization and reprocessing therapy (EMDR), and narrative exposure therapy, among others, have been provided by or under the supervision of psychologists or psychiatrists. Children and adolescents can benefit from these psychological interventions, which could significantly promote their mental health and well-being. Various psychological intervention programs in terms of theoretical background, methodology, content, and duration were proposed. Several studies have been conducted to evaluate effective interventions. Previous systematic reviews and meta-analyses have aggregated the results from these studies of interventions in children and adolescents exposed to disasters, finding psychological interventions efficacious at reducing PTSD symptoms [6–8].

The previous meta-analyses have two shortcomings. First, the previous meta-analyses mainly target PTSD as the primary outcome of natural and man-made disasters, estimating the effectiveness of psychological interventions for reducing PTSD symptoms. Although depression was often found to co-occur with PTSD in children and adolescents after traumatic events, the symptoms or mechanisms of these mental disorders differed, making psychological intervention effects on PTSD and depression inconsistent. Further extending this work, the current meta-analysis was conducted to explore not only the effect sizes of psychological intervention on PTSD but also on depression. Second, the existing meta-analyses used standard meta-analytic techniques and evaluated effect sizes based on direct evidence, which limits the comparison of each psychological intervention’s effectiveness to others. In addition, psychologists or psychiatrists need to choose the most effective programs for children and adolescents after disasters, among numerous psychological interventions. However, traditional meta-analysis cannot provide the first choice of intervention and the possible rank for each psychological intervention.

Network meta-analysis (NMA) is a mixed treatment comparison meta-analysis and multiple treatment comparison meta-analysis. NMA is developed from classical meta-analysis, extending the standard meta-analysis that only deals with two interventions to a method that simultaneously compares several interventions with each other and performs comprehensive ranking. This means that NMA could estimate the relative effects of multiple interventions based on indirect evidence and rank the effectiveness of a particular outcome to select the most appropriate treatment plan [9]. This meta-analysis was conducted to estimate the relative effectiveness of psychological interventions for PTSD and depression in children and adolescents after exposure to natural and man-made disasters using NMA.

## Methods

### Protocol and registration

The study protocol for the current NMA was registered in the International Prospective Register of Systematic Reviews (PROSPERO) on May 6, 2023. The registration number is CRD42023421304. The design and reporting of the study followed the Preferred Reporting Items for Systematic Review and Meta-Analyses (PRISMA) guidelines [10].

### Literature search

Searches were conducted in the following electronic databases in June 2023: MEDLINE, PubMed, Web of Science, PsycINFO, and EMBASE. Articles were identified by using the following terms: (disaster* or mass trauma or earthquake* or tsunami* or flood* or hurricane* or terroris*) and (intervent* or counsel* or support* or treatment or therapy) and (randomized controlled trials or randomized control trial or random*) and (child* or adolescent* or youth or teen*). Searches were limited to children and adolescents (under 18 years) and English publications. In addition, five systematic reviews on relevant topics were identified, and the reference lists of these reviews were examined for potential studies [6–8, 11, 12].

### Inclusion criteria

Studies included in this NMA were required to meet the following inclusion criteria: (1) randomized controlled trials (RCTs), (2) children or adolescents exposed to natural and man-made disasters, (3) assessed the efficacy of at least a psychological intervention, (4) compared the psychological intervention with inactive intervention or other interventions, (5) reported validated measurement of PTSD or depression, and (6) reported the mean score and standard deviation (SD) or provided other related information to estimate effect sizes. If one data set were published in a journal article and a book simultaneously, we would include the journal article.

The authors (YX and CS) independently examined the articles for eligibility, and any disagreements were resolved through discussion. Online supplemental material outlines the reasons for the exclusion of full-text articles. After assessing the full text of articles for eligibility, 26 studies were included in the current review.

### Data extraction

NMA outcomes included PTSD symptoms and depression symptoms, which were measured on relative rating scales. The authors (YX and SD) collected these outcomes’ mean scores and SD at the treatment endpoint and follow-up. The scores obtained nearest to the end of the intervention are identified as postintervention scores, while those assessed at least one month after the intervention’s completion are identified as follow-up scores. When multiple scales are used to assess symptoms of PTSD or depression, all scores were extracted and analyzed together. If studies failed to report SD, we calculated SD from other related information, such as standard errors, confidence intervals, t-values, or p-values [13]. In addition, we extracted data from the included studies. This data consisted of author, year, type of trauma, intervention, comparison, number of participants in each group, average age, female percentage, level of intervention, PTSD measurement, and depression measurement.

### Quality assessment

The authors (SD and CX) performed the quality assessment of these included studies using version 2 of the Cochrane risk-of-bias tool for randomized trials (RoB 2) [14]. The risk of bias for each study was assessed in five domains, including randomization, deviations from intended interventions, missing data, outcome measurement, and selection of reported results. Overall, studies were regarded as high risk of bias if one domain of RoB 2 was found to be at high risk. Studies were determined as low risk of bias if all domains of RoB 2 were judged to be at low risk. All other cases were judged to be of some concern.

### Statistical analysis

The current NMA was conducted with the *mvmeta* package in Stata14.0 and OpenBUGS [15, 16]. The standardized mean differences (SMDs) between pairs of interventions at postintervention and follow-up were pooled to synthesize outcomes because the included studies used different rating scales to report PTSD and depression symptoms. Interventions belonging to a similar theory were combined into a single group. Mean effect sizes with 95% credible intervals (CI) were calculated for each analysis. In addition, the ranking probabilities for all interventions were estimated using the surface under the cumulative ranking curve (SUCRA), which was a percentage of the effectiveness of each intervention that would be ranked first [17]. The heterogeneity across the comparisons was estimated in the network [18]. Subgroup analyses by level of intervention (individual vs. group intervention), profession treatment provider (psychologist/psychiatrist vs. teacher/counselor), and country income (HICs vs. LMICs) were conducted.

These psychological interventions belonging to the same theory or technique were combined into a single node. Cognitive Behavioral Therapy (CBT) is a relatively broad category of psychological interventions, and any intervention that employs cognitive behavioral techniques is classified under this category. Exposure techniques such as narrative, writing, and imaginal reliving were classified together as Exposure Therapy (ET). Psychological interventions that employ mindfulness techniques or theories are classified under Mindfulness Based Therapy (MBT). Treatment as usual (TAU) primarily consists of general support measures, such as routine courses and training methods. Waiting list (WL) and no treatment (NT) were combined. Eye Movement Desensitisation and Reprocessing (EMDR), Trauma and Grief Component Therapy (TGCT), Building Resilience Intervention (BRI), and Play Therapy (PT), which cannot be classified into the aforementioned categories, form a category of their own.

Consistency in a network of interventions refers to the similarity between direct and indirect evidence in comparisons [19, 20]. The inconsistency test was performed by comparing direct and indirect evidence in the closed loop of nodes [21, 22] and assessing the goodness of fit for the NMA model [23]. In addition, the possible inconsistency was investigated using a side-splitting approach between direct and indirect evidence. The likelihood-ratio test was conducted to examine the consistency model.

Publication bias was evaluated for each comparison by conducting global funnel plot analyses. The symmetry of funnel plots was visually inspected to distinguish publication bias [24]. Sensitivity analyses were performed by excluding studies with high bias risk. In addition, we assessed the certainty of evidence using Confidence in NMA (https://cinema.ispm.unibe.ch/). This web application evaluates the confidence of findings for NMA [25].

## Results

### Characteristics of included studies

**Fig. 1:**
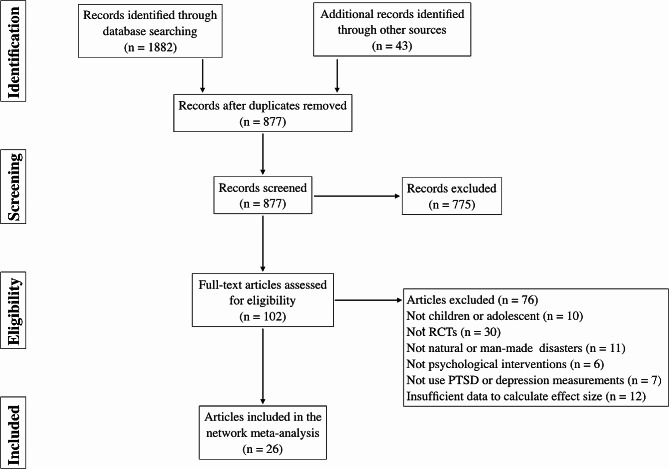
The PRISMA diagram

The flow diagram of study selection is presented in Fig. 1. After removing duplicates, 877 articles were included in the initial title and abstract screening, 102 of which were selected for full-text assessment. Seventy-six articles failed to meet the criteria for the current NMA, leaving 26 eligible articles that reported at least one outcome of interest.

Table 1 shows the characteristics of the included studies. The number of participants was 4331, while their mean age was 12.69 years (ranging from 8.40 to 16.30 years). The mean sample size of the included studies was 166.58, ranging from 26 to 1220. One study included only females, and the other had only males. In the remaining studies, the average percentage of females was 54.93, varying from 34.67 to 74.12. Seven studies were conducted in high-income countries (HICs), and nineteen in low/middle-income countries (LMICs). Eleven studies were carried out by psychologists or psychiatrists, and fifteen studies were carried out by teachers or counselors. Twenty-three studies recruited participants with measurements of PTSD symptoms using the CPSS, the CPTS-RI, the CRI, the CRIES, the HTQ, the PSS, the RI, the TGIC, the UCLA Grief Inventory, and the UCLA PTSD Index. Fifteen studies recruited participants with measurements of depression symptoms using the APAI, the BDS, the CDI, the CES-D, the DSRS, the MDI, the SCARED, and the SSRS. Twelve studies compared a psychological intervention with WL, and eight employed NT as the control condition. Twenty-four psychological interventions were included in the current research, including BRI, CBT, CP, CT, EMDR, EMDR-GP/C, ET, GSI, IPT-G, KIDNET, MBSGP, MED-RELAX, m-WET, NET, OT, PBT, PS, PY, SBT, SSET, TAU, TF-CBT, TGCT, TRT, and WR.

**Table 1:** Characteristics of included studies

Study	Type of Trauma	Intervention	Intervention Group, *n*	Comparison	Comparison Group, *n*	Number of sessions	Follow-up Duration	Country and Territory	Mean Age	%Female	Group/ Individual	Profession of Treatment Provider	PTSD Measure	Depression Measure
Ahmadi, 2022 [26]	Man-made disaster	m-WET/TF-CBT	40/40	NT	40	5	3 months	Afghanistan	15.99	100	Group	Psychologist	CRIES	N.A.
Amin, 2020 [27]	Natural disaster	SSET	38	NT	37	10	N.A.	Pakistan	11.43	34.67	Group	Program trainee	CPSS	N.A.
Bahar, 2008 [28]	Natural disaster	PBT	73	OT	114	3	N.A.	Turkey	13.33	48.12	Group	Psychiatrist	N.A.	CDI
Banoglu, 2022 [29]	Man-made disaster	EMDR	47	WL	47	3–4	N.A.	Turkey	N.A.	40.98	Group	Psychologist	CPTS-RI	MDI
Barron, 2016 [30]	Man-made disaster	TRT	79	WL	75	5	N.A.	Palestine	N.A.	59.71	Group	School counselor	CRIES	DSRS
Baum, 2013 [31]	Man-made disaster	BRI	310	WL	359	N.A.	N.A.	Israel	10.85	48.78	Group	Teacher	UCLA PTSD Index	SCARED
Bolton, 2007 [32]	Man-made disaster	CP/IPT-G	105/105	WL	104	16	4 months	Uganda	14.97	57.32	Group	Program trainee	N.A.	APAI
Catani, 2009 [33]	Natural and man-made disasters	KIDNET	16	TAU	15	6	6 months	Sri Lanka	11.94	45.16	Individual	Local counselor	UCLA PTSD Index	N.A.
Chemtob, 2002 [34]	Natural disaster	EMDR	17	WL	15	3	6 months	USA	8.4	68.75	Individual	Clinicians	CRI	CDI
Chen, 2014 [35]	Natural disaster	CBT	16	GSI /NT	12/12	6	3 months	China	14.50	67.50	Individual	Program trainee	CRIES	CES-D
Dawson, 2017 [36]	Man-made disaster	CBT	32	PS	32	5	3 months	Hati	10.70	46.88	Individual	Lay counselor	UCLA PTSD Index	CDI
de Roos, 2011 [37]	Man-made disaster	CBT	26	EMDR	26	4	3 months	Netherland	10.10	44.23	Individual	Clinicians	UCLA PTSD Index/ CROPS	BDS
Dhital, 2019 [38]	Natural disaster	SBT	605	NT	615	8	6 months	Nepal	12.90	53.52	Group	Teacher	CPSS	SSRS
Gordon, 2008 [39]	Man-made disaster	MBSGP	41	WL	41	12	N.A.	Kosovo	16.30	73.17	Group	Teacher	HTQ	N.A.
Jordans, 2010 [40]	Man-made disaster	SBT	164	WL	161	15	N.A.	Nepal	12.70	48.62	Group	Program trainee	CPSS	DSRS
Kalantari, 2012 [41]	Man-made disaster	WR	29	NT	32	6	N.A.	Afghanistan	14.82	52.46	Group	Teacher	TGIC	N.A.
Layne, 2008 [42]	Man-made disaster	TGCT	66	TAU	61	17	4 months	Bosnia	15.95	64.57	Group	Counselor	UCLA Grief Inventory	DSRS
Mahmoudi-Gharaei, 2009 [43]	Natural disaster	CBT	36	NT	49	4	N.A.	Iran	N.A.	74.12	Group	Psychiatrist	PSS	N.A.
Mcmullen, 2013 [44]	Man-made disaster	TF-CBT	25	WL	25	15	N.A.	DR Congo	15.75	0	Group	Psychologist	UCLA PTSD Index	N.A.
Ooi, 2016 [45]	Man-made disaster	CBT	45	WL	37	8	3 months	Australia	12.64	64.63	Group	Program trainee	CRIES	DSRS
Pityaratstian, 2015 [46]	Natural disaster	CBT	18	WL	18	5	1 month	Thailand	12.25	72.22	Group	Psychiatrist	CRIES/ UCLA PTSD Index	N.A.
Qouta, 2012 [47]	Man-made disaster	TRT	242	WL	240	16	6 months	Palestine	11.29	49.38	Group	Psychologist	CRIES	DSRS
Ronan, 1999 [48]	Natural disaster	CBT	69	ET	43	1	4 months	New Zealand	10.50	53.98	Group	Psychologist	RI	N.A.
Ruf, 2010 [49]	Man-made disaster	NET	13	WL	13	8	12 months	Germany	11.45	46.15	Individual	Psychologist	UCLA PTSD Index	N.A.
Shen, 2002 [50]	Natural disaster	PT	15	NT	15	10	N.A.	China, Taiwan	N.A.	53.33	Group	School counselor	N.A.	MDI
Zhu, 2014 [51]	Natural disaster	CT	129	NT	81	30	N.A.	China	10.51	50.00	Group	Teacher	CRIES	N.A.

Abbreviations: APAI: Acholi Psychosocial Assessment Instrument; BASC: Behavioral Assessment System for Children; BDS: Birleson Depression Scale; BRI: Building Resilience Intervention; CBT: Cognitive Behavioral Therapy; CDI: Children’s Depression Inventory; CES-D: Center for Epidemiologic Studies Depression Scale; CP: Creative Play; CPSS: Child PTSD Symptoms Scale; CPTS-RI: Child Posttraumatic Stress Reaction Index; CROPS: Child Report of Post-traumatic Symptoms; CRI: Child PTSD Reaction Index; CRIES: Child Revised Impact of Event Scale; CT: Calligraphy Training; DSRS: Depression Self-Rating Scale; EMDR: Eye Movement Desensitisation and Reprocessing; ET: Exposure Therapy; GSI: General Supportive Intervention; HTQ: Harvard Trauma Questionnaire; IPT-G: Interpersonal Psychotherapy for Groups; KIDNET: Narrative Exposure Therapy for Children; MBSGP: Mind-Body Skills Group Program; MDI: Major Depression Inventory; m-WET: Modified Written Exposure Therapy; NET: Narrative Exposure Therapy; NT: No Treatment; OT: Occupational Therapy; PBT: Problem-Based Therapy; PS: Problem-solving; PSS: Post-traumatic Stress Scale; PT: Play Therapy; RI: Reaction Index; SBT: School Based Treatment; SSET: Support for Students Exposed to Trauma; SSRS: Depression Self-Rating Scale; TAU: Treatment as Usual; TF-CBT: Trauma-Focused Cognitive Behavior Therapy; TGCT: Trauma and Grief Component Therapy; TGIC: Traumatic Grief Inventory for Children; TRT: Teaching Recovery Techniques; UCLA: University of California, Los Angeles; WL: Waiting List; WR: Writing for Recovery

### Risk of bias assessment

**Fig. 2:**
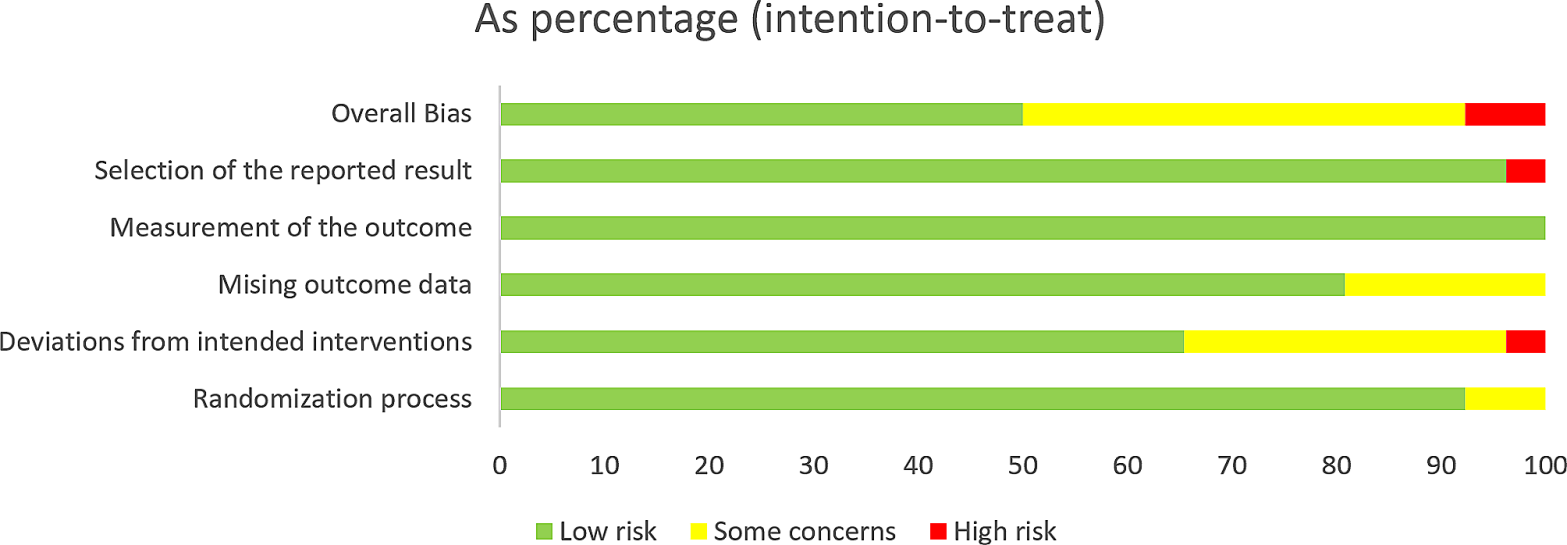
Risk of bias across all included studies

**Fig. 3:**
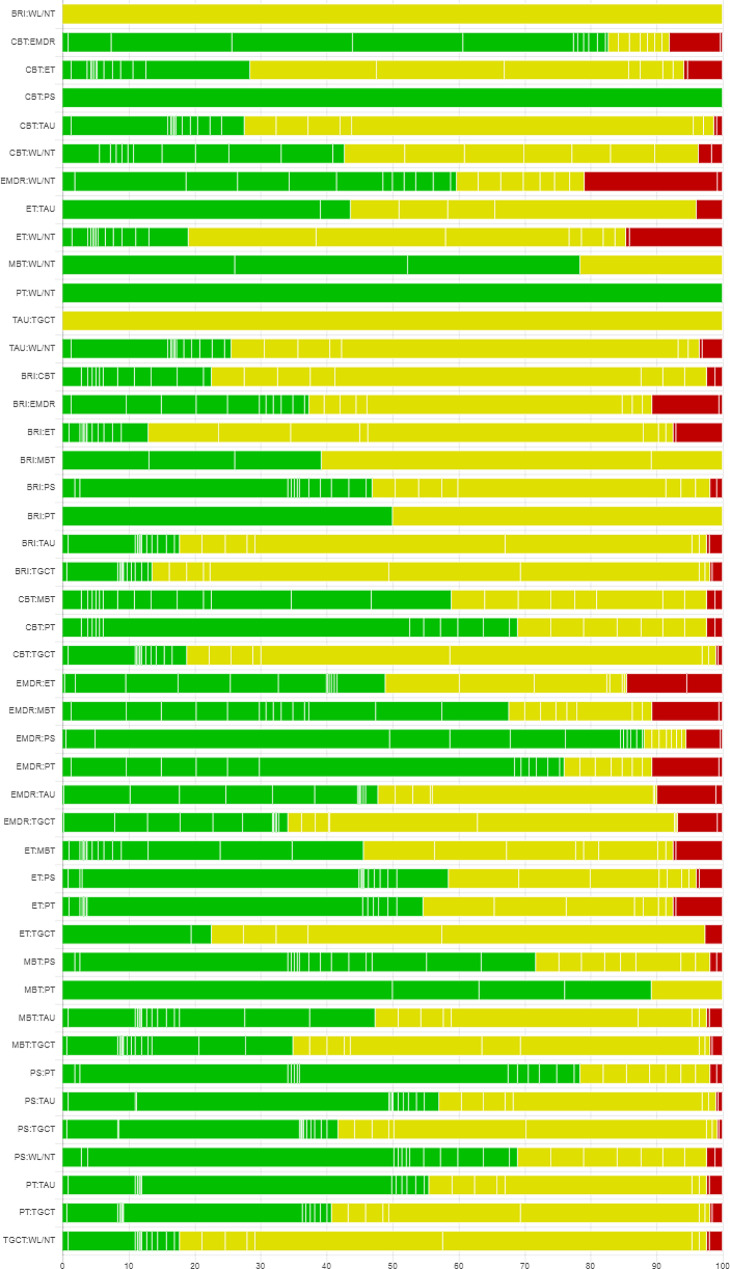
Risk of bias bar chart for the comparison of psychological interventions

All 26 included studies were assessed for risk of bias using RoB 2. Two studies were at high risk of bias, thirteen were at low risk of bias, and eleven had some concerns. Each risk of bias item, presented as percentages across all included studies, is shown in Fig. 2, and each risk-of-bias item for each included study is provided in online supplemental material. Combined with the risk of bias judgments, the certainty of evidence is shown in Fig. 3.

### PTSD symptoms at postintervention

**Fig. 4:**
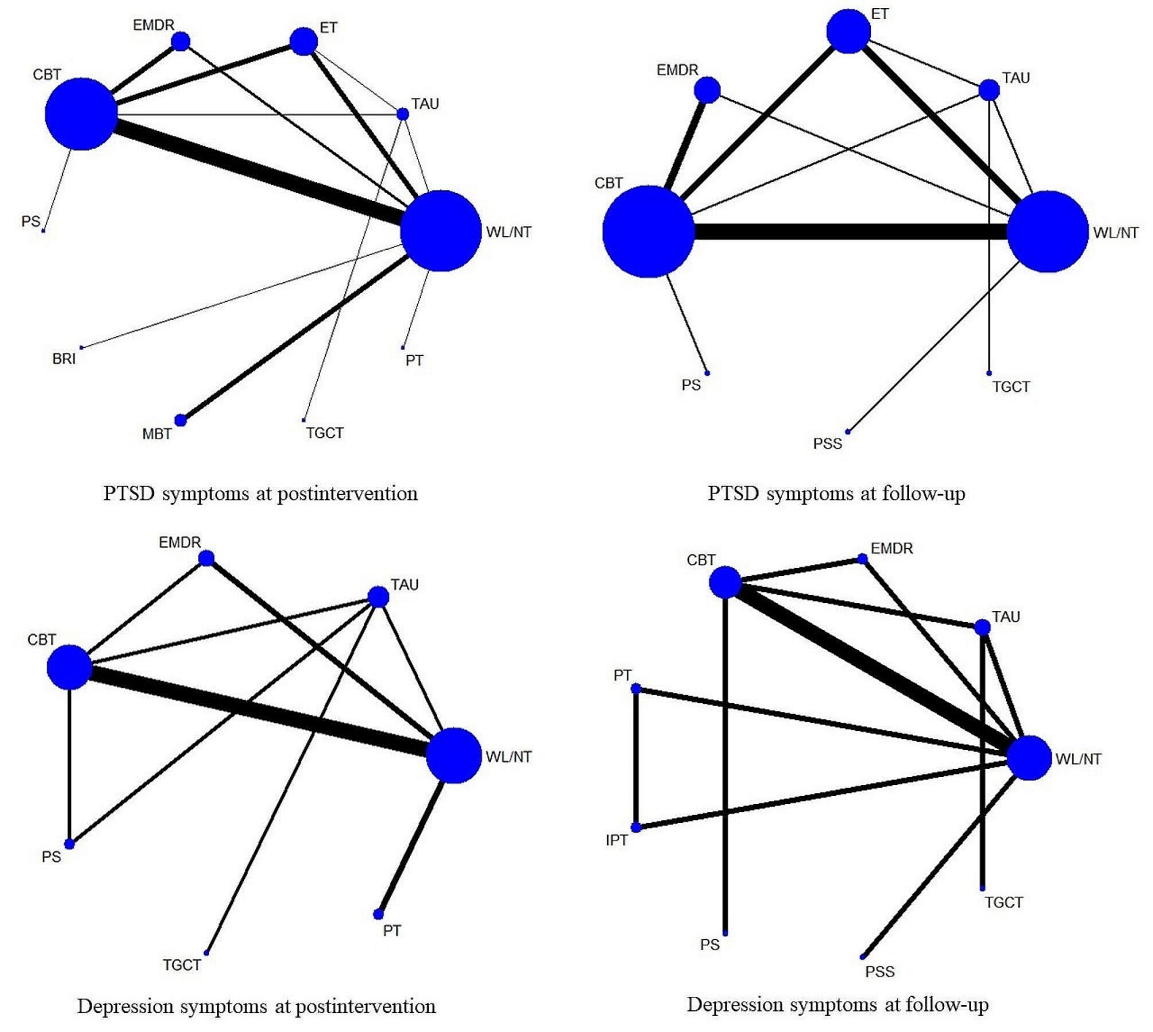
Network plots of evidence for PTSD and depression symptoms at postintervention and follow-up

**Fig. 5:**
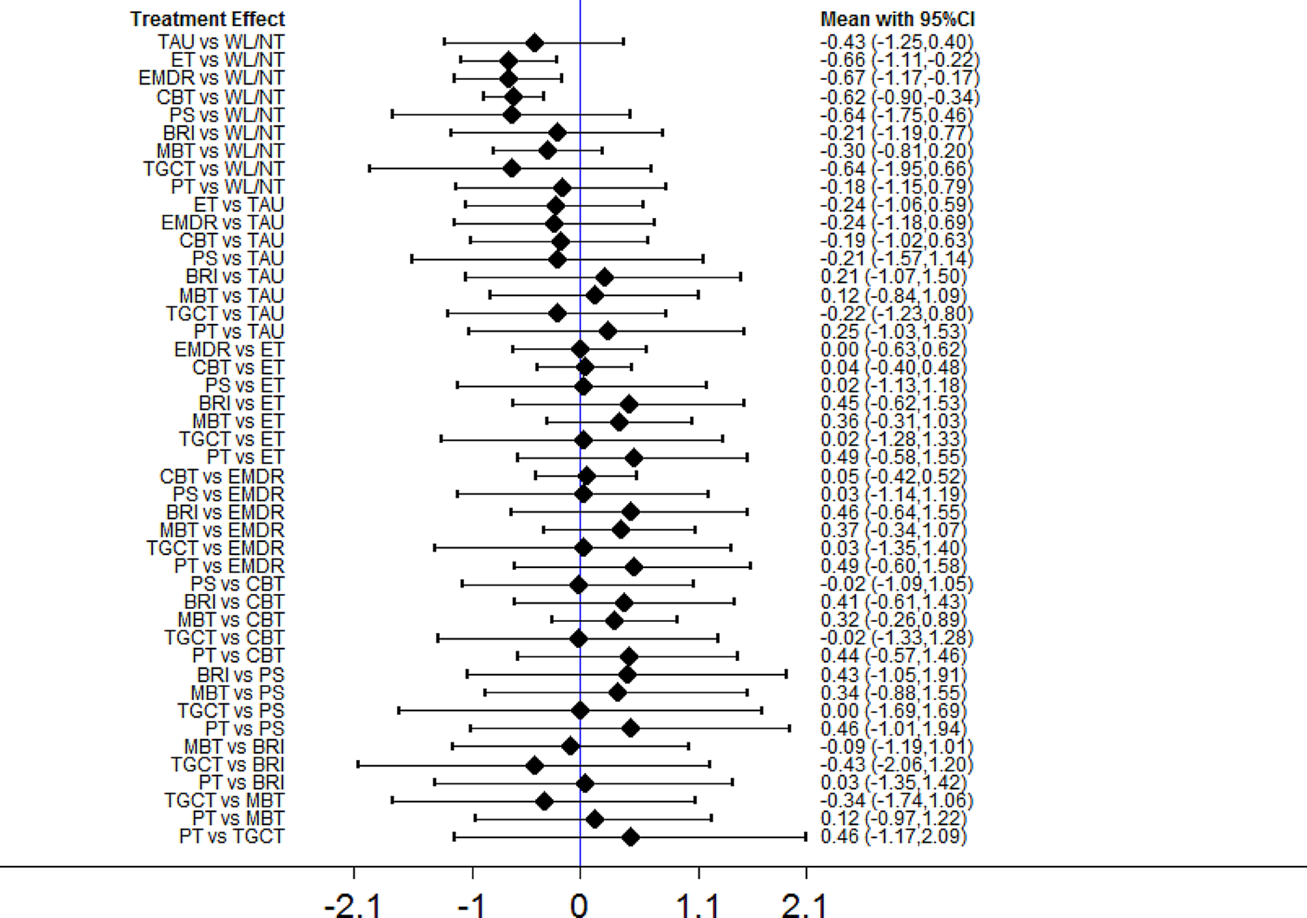
Forest plots of head-to-head comparisons for PTSD symptoms at postintervention

Figure 4 presents the network plot of psychological interventions for the PTSD symptoms at postintervention, Table 2 shows the results of each analysis, and Fig. 5 illustrates the forest plots of head-to-head comparisons. EMDR (SMD = − 0.67; 95% CI − 1.17 to − 0.17), ET (SMD = − 0.66; 95% CI − 1.11 to − 0.22), and CBT (SMD = − 0.62; 95% CI − 0.90 to − 0.34) were significantly more effective than inactive intervention. For the other comparisons, the differences were not significant. The heterogeneity of pairwise comparisons was not found, except for one comparison (CBT versus inactive intervention, *I*^*2*^ = 90.6). The design-by-treatment test was *P* = 0.960, indicating that the overall incoherence was not substantial. Loop-specific heterogeneity results were not significant for all five loops. There was no statistical disagreement between direct and indirect evidence for each comparison. The mean rank of each psychological intervention was estimated, indicating that EMDR and ET were ranked best according to the SUCRA and cumulative probability plots (online supplemental material). Results of sensitivity analyses confirmed that EMDR had the best performance compared with other interventions.

**Table 2:** Results of NMA of psychological interventions for PTSD and depression at postintervention and follow-up in children and adolescents

PSTD at postintervention			
Intervention	SMD (95% CI) vs. WL/NT	Mean ranking	SUCRA
EMDR	-0.67 (-1.17, -0.17)	3.7	69.6
ET	-0.66 (-1.11, -0.22)	3.7	69.6
CBT	-0.62 (-0.90, -0.34)	4.1	66.0
PS	-0.64 (-1.75, 0.46)	4.3	63.6
TGCT	-0.64 (-1.95, 0.66)	4.5	61.6
TAU	-0.43 (-1.25, 0.40)	5.7	47.8
MBT	-0.30 (-0.81, 0.20)	6.5	38.5
BRI	-0.21 (-1.19, 0.77)	6.8	35.4
PT	-0.18 (-1.15, 0.79)	7.0	33.4
WL/NT	Reference	8.7	14.5
PSTD at follow-up			
Intervention	SMD (95% CI) vs. WL/NT	Mean ranking	SUCRA
EMDR	-0.72 (-1.11, -0.33)	1.9	87.1
ET	-0.62 (-0.97, -0.27)	2.5	78.9
TGCT	-0.45 (-1.34, 0.44)	3.9	58.6
CBT	-0.43 (-0.67, -0.19)	4.0	56.6
TAU	-0.34 (-0.94, 0.26)	4.7	47.7
PS	-0.18 (-0.88, 0.52)	5.5	35.1
PSS	-0.05 (-0.51, 0.40)	6.5	21.5
WL/NT	Reference	7.0	14.5
Depression at postintervention			
Intervention	SMD (95% CI) vs. WL/NT	Mean ranking	SUCRA
EMDR	-0.40 (-0.78, -0.03)	1.7	88.5
PT	-0.37 (-0.62, -0.12)	1.8	87.3
PS	0.00 (-0.45, 0.46)	4.3	44.3
CBT	-0.00 (-0.17, 0.17)	4.5	41.0
TGCT	0.09 (-0.54, 0.71)	5.0	33.7
TAU	0.18 (-0.31, 0.67)	6.1	15.8
WL/NT	Reference	4.6	39.5
Depression at follow-up			
Intervention	SMD (95% CI) vs. WL/NT	Mean ranking	SUCRA
IPT	-0.69 (-1.73, 0.34)	2.7	79.2
EMDR	-0.38 (-1.28, 0.52)	3.9	64.2
TAU	-0.25 (-1.40, 0.89)	4.6	55.5
CBT	-0.18 (-0.80, 0.43)	4.8	52.3
TGCT	-0.19 (-1.78, 1.40)	4.9	50.8
PS	-0.06 (-1.33, 1.20)	5.4	44.9
PSS	0.03 (-0.97, 1.02)	5.9	38.8
PT	0.22 (-0.82, 1.25)	6.8	27.2
WL/NT	Reference	6.0	37.1

### PTSD symptoms at follow-up

**Fig. 6:**
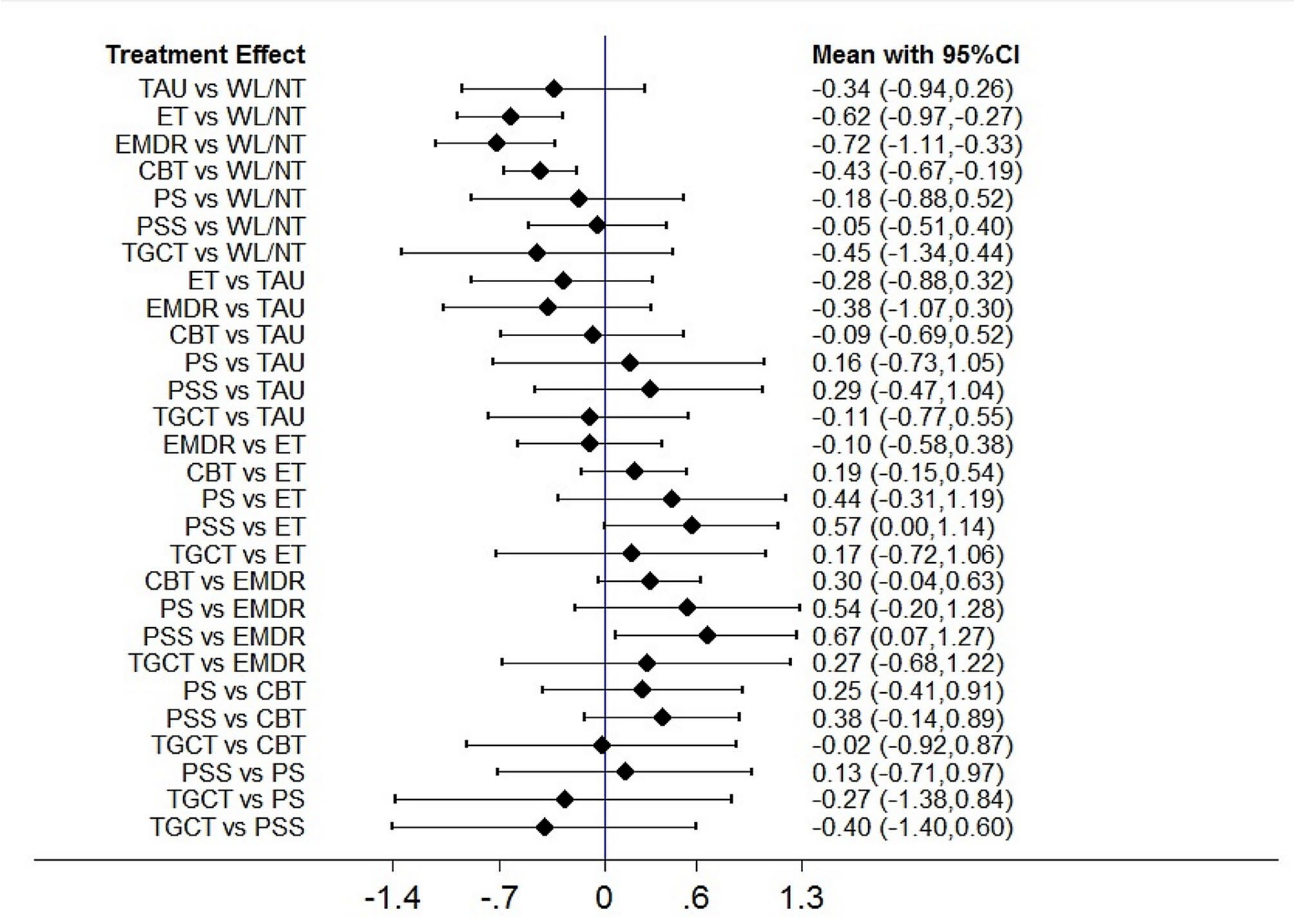
Forest plots of head-to-head comparisons for PTSD symptoms at follow-up

Figure 4 presents the network plot of psychological interventions for PTSD symptoms at follow-up, Table 2 shows the results of each analysis, and Fig. 6 illustrates the forest plots of head-to-head comparisons. EMDR (SMD = − 0.72; 95% CI − 1.11 to − 0.33) and ET (SMD = − 0.62; 95% CI − 0.97 to − 0.27) were significantly more effective than the inactive intervention. The differences were insignificant for other psychological interventions compared with the inactive intervention. The heterogeneity of pairwise comparisons was not found. The design-by-treatment test was *P* = 0.178, suggesting that overall incoherence was not significant. Significant loop-specific heterogeneity emerged for one loop involving ET, CBT, and TAU (*Z* = 2.265, *P* < 0.05). No evidence of statistical disagreement between direct and indirect evidence for each comparison was found, except for the comparison between CBT and TAU (*P* < 0.05). The mean rank of each psychological intervention was estimated, indicating that EMDR was ranked best according to the SUCRA and cumulative probability plots (online supplemental material).

### Depression symptoms at postintervention

**Fig. 7:**
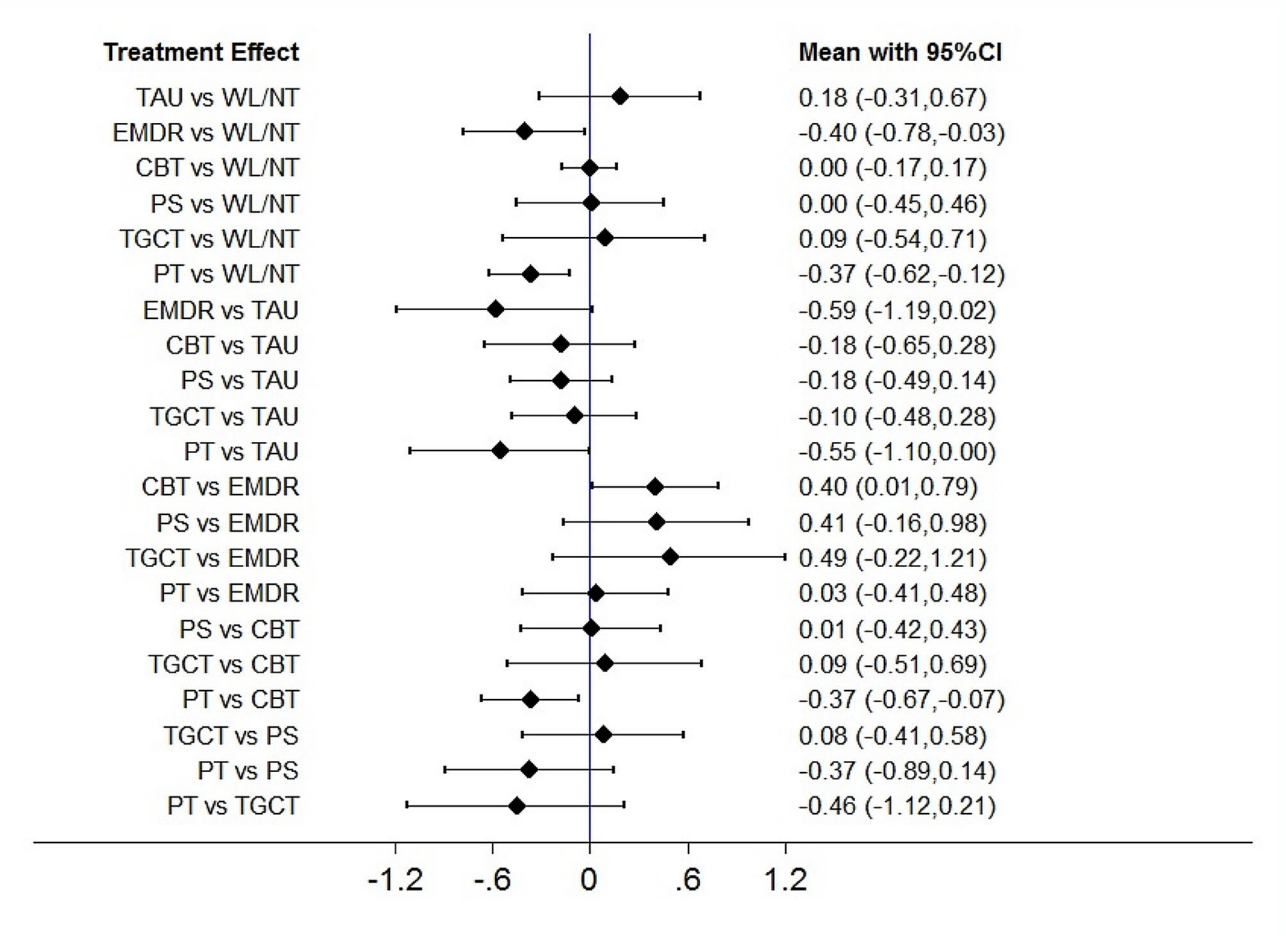
Forest plots of head-to-head comparisons for depression symptoms at postintervention

Figure 4 presents the network plot of psychological interventions for depression symptoms at postintervention, Table 2 shows the results of each analysis, and Fig. 7 illustrates the forest plots of head-to-head comparisons. EMDR (SMD = − 0.40; 95% CI − 0.78 to − 0.03) and play therapy (PT) (SMD = − 0.37; 95% CI − 0.62 to − 0.12) were significantly more effective than the inactive intervention. The differences were insignificant for other psychological interventions compared with the inactive intervention. The heterogeneity of pairwise comparisons was not found. The design-by-treatment test was *P* = 0.175, suggesting that overall incoherence was not significant. Intra-loop incoherence was not substantial for all three loops. No evidence of statistical disagreement between direct and indirect evidence for each comparison was found, except for the comparison between CBT and WL/NT (*P* < 0.05). The mean rank of each psychological intervention was estimated, indicating that EMDR was ranked best according to the SUCRA and cumulative probability plots (online supplemental material).

### Depression symptoms at follow-up

**Fig. 8:**
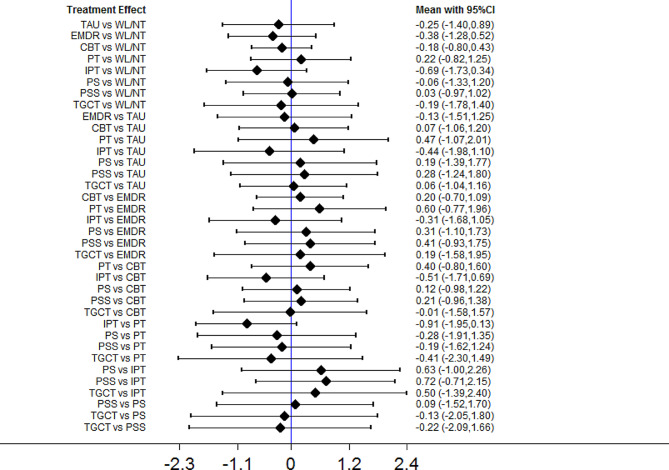
Forest plots of head-to-head comparisons for depression symptoms at follow-up

Figure 4 presents the network plot of psychological interventions for depression symptoms at follow-up, Table 2 shows the results of each analysis, and Fig. 8 illustrates the forest plots of head-to-head comparisons. The differences were not significant for all psychological interventions compared with inactive interventions. The heterogeneity of pairwise comparisons was not found. The design-by-treatment test was *P* < 0.01, suggesting overall incoherence. Significant loop-specific heterogeneity emerged for one loop involving CBT, TAU, and WL/NT (*Z* = 2.353, *P* < 0.05). There was no statistical disagreement between direct and indirect evidence for each comparison except for two comparisons (WL/NT and TAU, *P* < 0.001; CBT and TAU, *P* < 0.001). The mean rank of each psychological intervention was estimated, indicating that EMDR was ranked best according to the SUCRA and cumulative probability plots (online supplemental material).

### Subgroup analyses

Subgroup analyses by level of intervention, profession of treatment provider, and country income level were performed (online supplemental material). For level of intervention, overall incoherence on PTSD and depression symptoms at postintervention and follow-up emerged. For individual interventions, EMDR ranked best on PTSD symptoms for both postintervention and follow-up. Regarding the profession of treatment provider, no overall incoherence was found between professionals and lay counselors on PTSD symptoms at postintervention. In the psychologist/psychiatrist category, ET showed the most favorable outcomes, and CBT showed the greatest improvement in the teacher/counselor category according to the mean SUCRA and cumulative probability plots. EMDR showed the greatest improvement in the psychologist/psychiatrist category on PTSD symptoms at follow-up, and PT showed the most favorable outcomes in the teacher/counselor category on depression symptoms at postintervention. For country income level on PTSD symptoms at postintervention, no overall incoherence between HICs and LMICs groups emerged. ET ranked best in LMICs, and EMDR ranked best in HICs according to the mean SUCRA and cumulative probability plots. For PTSD symptoms at follow-up, ET ranked best in LMICs. The remaining subgroup analyses were not performed due to unavailability of pairs of interventions with both direct and indirect comparisons.

### Publication bias

Global funnel plots for outcomes were performed, as documented in the online supplemental material. There was potential publication bias for PTSD symptoms at postintervention, while there was no potential publication bias for PTSD symptoms at follow-up. No evidence of potential publication bias was shown for depression symptoms, either post-intervention or at follow-up. Visual inspections of funnel plots for subgroups were also conducted and are presented in the online supplemental material.

## Discussion

The current NMA comprised results from 26 studies on 4331 children and adolescents exposed to natural or man-made disasters, estimating the relative intervention effects of psychological interventions for participants with PTSD symptoms and depression symptoms. The rank probability for each psychological intervention was calculated to evaluate the most effective interventions at postintervention and 1–12 month follow-up. Overall, the results showed some psychological interventions to be effective for PTSD and depression symptoms compared to control groups.

In these included studies, EMDR, ET, and CBT were found to reduce PTSD symptoms significantly at postintervention. Similar results were obtained at follow-up, with EMDR and ET appearing to be effective interventions. Compared with inactive intervention, the results for other interventions were inconclusive because 95% CI of effects crossed the line of effect. EMDR was found at the top of the hierarchy, suggesting it to be the most effective intervention for reducing PTSD symptoms in children and adolescents with PTSD at postintervention and follow-up. Based on the included studies in this NMA, EMDR was found to have the greatest effectiveness at postintervention and follow-up and should be recommended. Although CBT had a slightly lower effect size than EMDR and ET, CBT was the most represented psychological intervention, suggesting that the evidence is more robust. However, the evidence of CBT’s long-term effectiveness is limited due to the insignificant effect size. The results of this NMA were consistent with previous reviews that used standard meta-analysis techniques. Brown and colleagues found CBT, EMDR, narrative exposure therapy for children (KIDNET), and classroom-based interventions have similar efficacy [6]. A meta-analysis conducted by Newman et al. also showed the significant effect sizes of psychological intervention on reducing PTSD symptoms in children and adolescents after disasters, finding that EMDR, ET, and Strict CBT appeared to have the largest effect sizes [7]. In line with previous meta-analyses, EMDR, ET, and CBT are effective at reducing PTSD symptoms in children and adolescents affected by natural and man-made disasters.

Our analysis also found that EMDR and PT may effectively treat depression in children and adolescents post-disaster at postintervention. However, all psychological interventions failed to show significant effect sizes at follow-up. Previous meta-analyses of intervention studies in children and adolescents exposed to disasters have found a range of results, from no effects on depression [12, 52] to minimal effects [53]. Consistent with previous studies, most psychological interventions showed no effect in the current review. Even though EMDR and PT significantly reduced depression symptoms at postintervention, the effect sizes were small. PTSD is the most prevalent mental disorder in children and adolescents exposed to natural and man-made disasters. Most psychological interventions were designed to treat PTSD rather than depression. Therefore, the effectiveness of depression interventions is relatively limited. One study that employed a short-term group PT substantially reduced depression and anxiety in children after the earthquake [50]. In addition, we also tried to explore the effect of intervention on anxiety, but the analysis failed due to insufficient studies.

EMDR effectively reduces PTSD and depression in children and adolescents affected by disasters, grounded in the Adaptive Information Processing (AIP) model [54]. This model posits that humans have a natural system for integrating new experiences into adaptive memory networks, linking experiences to thoughts, images, emotions, and sensations. Disorders arise when information is improperly processed, leading to maladaptive storage in these networks, re-triggering traumatic memories through similar stimuli. The AIP model suggests that proper processing of these memories, especially using EMDR’s bilateral stimulation such as eye movements, can alleviate symptoms and promote healing. During EMDR therapy, children and adolescents concentrate on the worst images in their traumatic memories while also following the therapist’s fingers with rhythmic, bilateral, pulsating eye movements. This dual attention task can process traumatic memories, thereby alleviating symptoms of PTSD and depression [55]. In contrast to conventional psychological interventions, EMDR surpasses the limitations inherent in traditional talk therapy modalities. It obviates the need for children and adolescents to engage in deep, focused contemplation of their traumatic experiences. Rather, through the process of eye movements, it facilitates the association and integration of targeted traumatic memories via associative pathways.

Some imitations should be accounted for when interpreting these NMA results. First, the number of studies included in this review was relatively low. Considering the quality of the studies, we selected studies that employed RCTs. Many researchers were urged to provide immediate intervention for children and adolescents following natural and man-made disasters. In many cases, it is impossible to have a group of participants with inactive interventions such as WL or NT conditions to control for spontaneous symptom remission due to ethical concerns [33]. Limiting studies to those published in English-language, peer-reviewed journals could increase the risk of publication bias. Second, the number of direct comparisons between active psychological interventions was also relatively low. Most of the findings were based on comparisons of indirect treatments, which are more likely to have biases. Moreover, the original studies did not adequately report the results at follow-up, which led to poorly connected networks. Third, subgroup and sensitivity analyses cannot fully interpret the statistical heterogeneity detected in some comparisons. Many aspects could impact heterogeneity, such as the number of participants in each group, the mean age of participants, outcome measures, sessions of interventions, and time since disasters. Overall coherence, the key statistical manifestation of transitivity, appeared accepted in most analyses. Four, WL and NT were combined into one group because they were inactive, and keeping them together was more appropriate than other active interventions. WL was found to be less effective than NT because participants in WL would be aware that they can receive interventions after the study period is over and are likely to retain mental disorder symptoms. In contrast, participants in NT may be more active in relieving symptoms of PTSD and depression [56]. It is therefore recommended to use this NMA’s findings with caution.

### Implications

The implications of this NMA are profound for the field of psychological interventions for children and adolescents exposed to natural or man-made disasters. The evidence suggests that EMDR stands out as the most effective intervention, based on the hierarchy of interventions; this indicates that it should be prioritized as a treatment option in the immediate aftermath of traumatic events. While the current NMA offers valuable insights into the immediate benefits of psychological interventions for disaster-affected youths, it also calls for a more in-depth and nuanced approach to research and treatment. Stakeholders, including clinicians, researchers, and policymakers, should take these findings into account when designing, implementing, and funding interventions for this vulnerable population.

## Conclusion

This NMA revealed that EMDR appears to be most efficacious in reducing PTSD and depression symptoms in children and adolescents exposed to natural and man-made disasters. In addition, ET and CBT are potentially effective in reducing PTSD symptoms at postintervention, while PT is effective in managing depression symptoms at the treatment endpoint. Other psychological interventions fail to affect this population. Further study is needed to support the results of the current NMA, as the evidence of findings was very limited. Moreover, more research is required to examine interventions’ long-term effectiveness in children and adolescents, particularly for depression symptoms.

### Electronic supplementary material

Below is the link to the electronic supplementary material.

Supplementary Material 1

## Data Availability

The datasets generated in this review are available from the corresponding author on reasonable request.
