# Supplementary material for: A network meta-analysis of psychological interventions for children and adolescents after natural and man-made disasters
Source: BMC Psychiatry. 2024 Jun 25;24:468. doi: 10.1186/s12888-024-05924-8 (PMC11201056; doi:10.1186/s12888-024-05924-8)
Supplement: Supplementary file 1 — Supplementary Material 1 [file 12888_2024_5924_MOESM1_ESM.docx]

**Online Supplemental Material**

[Exclusion with reasons 2](#_Toc162661383)

[Risk of bias of included studies 8](#_Toc162661384)

[Outcome1：PTSD - post 9](#_Toc162661385)

[Outcome2：PTSD - follow 14](#_Toc162661386)

[Outcome3：Depression-post 18](#_Toc162661387)

[Outcome4：Depression- follow 22](#_Toc162661388)

[Sensitivity analysis: excluding trials with a high risk of bias 26](#_Toc162661389)

[Subgroup analysis : Level of Intervention 32](#_Toc162661390)

[Subgroup analysis : Profession of Treatment Provider 38](#_Toc162661391)

[Subgroup analysis : Country Income 52](#_Toc162661392)

[CINeMA-Final report 65](#_Toc162661393)

# Exclusion with reasons

1. Basoglu, M., et al. (2005). "Single-session behavioral treatment of earthquake-related posttraumatic stress disorder: A randomized waiting list controlled trial." JOURNAL OF TRAUMATIC STRESS **18**(1): 1-11. Not children or adolescent
2. Berger, R. and M. Gelkopf (2009). "School-based intervention for the treatment of tsunami-related distress in children: A quasi-randomized controlled trial." PSYCHOTHERAPY AND PSYCHOSOMATICS **78**(6): 364-371.Not RCTs
3. Berger, R., et al. (2007). "School-based intervention for prevention and treatment of elementary-students' terror-related distress in Israel: A quasi-randomized controlled trial." JOURNAL OF TRAUMATIC STRESS **20**(4): 541-551. Not RCTs
4. Berger, R., et al. (2012). "A teacher-delivered intervention for adolescents exposed to ongoing and intense traumatic war-related stress: A quasi-randomized controlled study." JOURNAL OF ADOLESCENT HEALTH **51**(5): 453-461. Not RCTs
5. Berger, R., et al. (2016). "Reducing Primary and Secondary Traumatic Stress Symptoms Among Educators by Training Them to Deliver a Resiliency Program (ERASE-Stress) Following the Christchurch Earthquake in New Zealand." AMERICAN JOURNAL OF ORTHOPSYCHIATRY **86**(2): 236-251. Not children or adolescent
6. Betancourt TS, McBain R, Newnham EA, et al. A behavioral intervention for war affected youth in Sierra Leone. J AM ACAD CHILD ADOLESC PSYCHIAT. 2014;53(12): 1288–1297. Not children or adolescent
7. Bolton, D., O’Ryan, D., Udwin, O., Boyle, S., & Yule, W. (2000). The long-term psychological effects of a disaster experienced in adolescence: II. General Psychopathology. JOURNAL OF CHILD PSYCHOLOGY AND PSYCHIATRY, *41*(4), 513–523. Not children or adolescent
8. Brown EJ, McQuaid J, Farina L, Ali R, Winnick-Gelles A (2006). Matching interventions to children’s mental health needs: feasibility and acceptability of a pilot school-based trauma intervention program. EDUCATION AND TREATMENT OF CHILDREN 29, 257–286. Not RCTs
9. Brown, E. J., et al. (2020). "An Exploratory Trial of Cognitive-Behavioral vs Client-Centered Therapies for Child-Mother Dyads Bereaved from Terrorism." JOURNAL OF CHILD & ADOLESCENT TRAUMA **13**(1): 113-125. Not children or adolescent
10. Chemtob, C. M., et al. (2002). "Psychosocial intervention for postdisaster trauma symptoms in elementary school children: A controlled community field study." ARCHIVES OF PEDIATRICS AND ADOLESCENT MEDICINE **156**(3): 211-216. Not RCTs
11. Dueweke, A. R., et al. (2021). "35.3 TREATING CHILD TRAUMA IN A LOWER-MIDDLE INCOME COUNTRY: IMPLEMENTATION OF TRAUMA-FOCUSED CBT IN EL SALVADOR." JOURNAL OF THE AMERICAN ACADEMY OF CHILD AND ADOLESCENT PSYCHIATRY **60**(10): S313. Not natural or man-made disasters
12. Ede, M. O., et al. (2022). "The Effect of Rational Emotive Behaviour Therapy on Post-Traumatic Depression in Flood Victims." JOURNAL OF RATIONAL-EMOTIVE AND COGNITIVE-BEHAVIOR THERAPY **40**(1): 124-143. Not children or adolescent
13. Ehntholt KA, Smith PA, Yule W (2005). School-based cognitive–behavioral therapy group intervention for refugee children who have experiences war-related trauma. CLINICAL CHILD PSYCHOLOGY AND PSYCHIATRY 10, 235–250. Not RCTs
14. Ertl, V., Pfeiffer, A., Schauer, E., Elbert, T., & Neuner, F. (2011). Community-implemented trauma therapy for former child soldiers in Northern Uganda. A randomized control trial. JAMA, *306*(5), 503–512. Not children or adolescent
15. Field, T., et al. (1996). "Alleviating posttraumatic stress in children following Hurricane Andrew." Journal of Applied Developmental Psychology **17**(1): 37-50. Insufficient data to calculate effect size
16. Froehlich-Grobe, K., et al. (2020). "Group Lifestyle Balance Adapted for Individuals With Impaired Mobility: Outcomes for 6-Month RCT and Combined Groups at 12 Months." AMERICAN JOURNAL OF PREVENTIVE MEDICINE **59**(6): 805-817. Not psychological interventions
17. Fu, C., Leoutsakos, J. M., & Underwood, C. (2013, December). Moderating effects of a postdisaster intervention on risk and resilience factors associated with posttraumatic stress disorder in Chinese children. JOURNAL OF TRAUMATIC STRESS, 26(6), 663–670 Insufficient data to calculate effect size
18. Gelkopf, M. and R. Berger (2009). "A school-based, teacher-mediated prevention program (ERASE-Stress) for reducing terror-related traumatic reactions in Israeli youth: a quasi-randomized controlled trial." Journal of child psychology and psychiatry, and allied disciplines **50**(8): 962-971. Not RCTs
19. Gerber, M. M., Hogan, L. R., Maxwell, K., Callahan, J. L., Ruggero, C. J., & Sundberg, T. (2014). Children after war: A novel approach to promoting resilience through music. TRAUMATOLOGY: AN INTERNATIONAL JOURNAL, 20(2), 112–118. Not RCTs
20. Giannopoulou I, Dikaiakou A, Yule W (2006). Cognitive–behavioural group intervention for PTSD symptoms in children following the Athens 1999 earthquake: a pilot study. CLINICAL CHILD PSYCHOLOGY AND PSYCHIATRY 11, 543–553. Not RCTs
21. Gilboa-Schechtman E et al. Prolonged exposure versus dynamic therapy for adolescent PTSD: a pilot randomized controlled trial. J AM ACAD CHILD ADOLESC PSYCHIATRY. 2010;49(10):1034–42. Not natural or man-made disasters
22. Gilmore, A. K., et al. (2018). "A Longitudinal Examination of Interpersonal Violence Exposure, Concern for Loved Ones During a Disaster, and Web-Based Intervention Effects on Posttraumatic Stress Disorder Among Adolescent Victims of the Spring 2011 Tornadoes." JOURNAL OF INTERPERSONAL VIOLENCE **36**(9-10): NP4611-NP4625. Not psychological interventions
23. Gil-Rivas, V., et al. (2004). "Adolescent vulnerability following the September 11th terrorist attacks: A study of parents and their children." APPLIED DEVELOPMENTAL SCIENCE **8**(3): 130-142. Not psychological interventions
24. Goenjian AK et al. Outcome of psychotherapy among early adolescents after trauma. AM J PSYCHIATRY. 1997;154(4):536–42. Not RCTs
25. Goenjian, A. K., Walling, D., Steinberg, A. M., Karayan, I., Najarian, L. M., & Pynoos, R. (2005, December). A prospective study of posttraumatic stress and depressive reactions among treated and untreated adolescents 5 years after a catastrophic disaster. THE AMERICAN JOURNAL OF PSYCHIATRY, 162(12), 2302–2308. Not RCTs
26. Hardayati, Y. A., et al. (2021). "The effects of thought stopping on anxiety levels in adolescents living in earthquake-prone areas." ENFERMERIA CLINICA **31**: S395-S399. Not natural or man-made disasters
27. Hardin, S. B., et al. (2002). "Effects of a long-term psychosocial nursing intervention on adolescents exposed to catastrophic stress." ISSUES IN MENTAL HEALTH NURSING **23**(6): 537-551. Not use PTSD or depression measurements.
28. Hechanova, M., et al. (2018). "Evaluation of a resilience intervention for Filipino displaced survivors of Super Typhoon Haiyan." DISASTER PREVENTION AND MANAGEMENT **27**(3): 346-359. Not RCTs
29. Hermenau K, Hecker T, Schaal S, Maedl A, Elbert T. Addressing post-traumatic stress and aggression by means of narrative exposure: a randomized controlled trial with ex-combatants in the Eastern DRC. J AGGRESS MALTREAT TRAUMA. 2013;22(8):916–934 Not children or adolescent
30. Hewitt-Ramirez, N., et al. (2020). "Efficacy of a Primary Care Mental Health Program for Victims of the Armed Conflict in Colombia." PEACE AND CONFLICT-JOURNAL OF PEACE PSYCHOLOGY **26**(1): 62-77. Not RCTs
31. Jacob, N., et al. (2014). "Dissemination of Psychotherapy for Trauma Spectrum Disorders in Postconflict Settings: A Randomized Controlled Trial in Rwanda." PSYCHOTHERAPY AND PSYCHOSOMATICS **83**(6): 354-363. Not children or adolescent
32. Jaycox, L. H., et al. (2010). "Children's Mental Health Care Following Hurricane Katrina: A Field Trial of Trauma-Focused Psychotherapies." JOURNAL OF TRAUMATIC STRESS 23(2): 223-231. Not RCTs
33. Karadag, M. and P. G. Karadeniz (2021). "Comparison of Group Eye Movement Desensitization and Reprocessing with Cognitive and Behavioral Therapy Protocol after the 2020 Earthquake in Turkey: A Field Study in Children and Adolescents." EUROPEAN JOURNAL OF THERAPEUTICS **27**(1): 40-44. Not use PTSD or depression measurements.
34. Karam, E. G., et al. (2008). "Effectiveness and specificity of a classroom-based group intervention in children and adolescents exposed to war in Lebanon." WORLD PSYCHIATRY **7**(2): 103-109. Insufficient data to calculate effect size
35. Kenardy, J., et al. (2010). "Protocol for a randomised controlled trial of risk screening and early intervention comparing child- and family-focused cognitive-behavioural therapy for PTSD in children following accidental injury." BMC PSYCHIATRY **10**. Not natural or man-made disasters
36. Kohrt, B. A., et al. (2015). "Alternative approaches for studying humanitarian interventions: propensity score methods to evaluate reintegration packages impact on depression, PTSD, and function impairment among child soldiers in Nepal." GLOBAL MENTAL HEALTH (CAMBRIDGE, ENGLAND) **2**: e16-e16. Insufficient data to calculate effect size
37. Lesmana, C. B. J., et al. (2009). "A spiritual-hypnosis assisted treatment of children with PTSD after the 2002 Bali terrorist attack." AMERICAN JOURNAL OF CLINICAL HYPNOSIS **52**(1): 23-34. Not RCTs
38. Li, J., et al. (2022). "The Acceptability, Feasibility, and Preliminary Effectiveness of Group Cognitive Behavioral Therapy for Chinese Children With Posttraumatic Stress Disorder: A Pilot Randomized Controlled Trial." PSYCHOLOGICAL TRAUMA-THEORY RESEARCH PRACTICE AND POLICY. Not natural or man-made disasters
39. Mehrotra S (2014). Humanitarian projects and growth of EMDR therapy in Asia. JOURNAL OF EMDR PRACTICE AND RESEARCH 8, 252–259. Not RCTs
40. Mueller, L. R. F., et al. (2021). "Screening for Posttraumatic Stress Symptoms in Young Refugees: Comparison of Questionnaire Data with and without Involvement of an Interpreter." INTERNATIONAL JOURNAL OF ENVIRONMENTAL RESEARCH AND PUBLIC HEALTH **18**(13). Not natural or man-made disasters
41. Niu, Y., et al. (2021). "Developing a resilience intervention approach for adolescents living with natural hazards risks: A pilot randomized controlled trial." INTERNATIONAL JOURNAL OF DISASTER RISK REDUCTION **58**. Not use PTSD or depression measurements.
42. O’Callaghan P, Branham L, Shannon C, Betancourt TS, Dempster M, McMullen JA. A pilot study of a family focused, psychosocial intervention with war-exposed youth at risk of attack and abduction in north-eastern Democratic Republic of Congo. CHILD ABUSE NEGL. 2014;38(7):1197–1207. Not natural or man-made disasters
43. O’Callaghan P, McMullen J, Shannon C, Rafferty H (2015). Comparing a trauma focused and non trauma focused intervention with war affected Congolese youth: a preliminary randomised trial. INTERVENTION 13, 28–44 NOT RCTS
44. O’Callaghan P, McMullen J, Shannon C, Rafferty H, Black A. A randomized controlled trial of trauma-focused cognitive behavioral therapy for sexually exploited, war-affected Congolese girls. J AM ACAD CHILD ADOLESC PSYCHIAT. 2013;52(4):359–369. Not natural or man-made disasters
45. Onyut, L. P., et al. (2004). "The Nakivale Camp Mental Health Project: Building local competency for psychological assistance to traumatised refugees." INTERVENTION: INTERNATIONAL JOURNAL OF MENTAL HEALTH, PSYCHOSOCIAL WORK & COUNSELLING IN AREAS OF ARMED CONFLICT **2**(2): 90-107. Not use PTSD or depression measurements.
46. Panter-Brick C, Dajani R, Eggerman M, Hermosilla S, Sancilio A, Ager A. Insecurity, distress and mental health: experimental and randomized controlled trials of a psychosocial intervention for youth affected by the Syrian crisis. J CHILD PSYCHOL PSYCHIAT. 2018;59(5):523–541 Not use PTSD or depression measurements.
47. Pityaratstian N, Liamwanich K, Ngamsamut N, Narkpongphun A, Chinajitphant N, Burapakajornpong N, Thongphitakwong W, Khunchit W, Weerapakorn W, Rojanapornthip B, Jayasvasti K (2007). Cognitive–behavioral intervention for young tsunami victims. JOURNAL OF THE MEDICAL ASSOCIATION THAILAND 90, 518–523 Not RCTs
48. Price, M., et al. (2015). "Access and Completion of a Web-based Treatment in a Population-Based Sample of Tornado-Affected Adolescents." PSYCHOLOGICAL SERVICES **12**(3): 283-290. Insufficient data to calculate effect size
49. Ruggiero, K. J., et al. (2015). "Bounce back now! protocol of a population-based randomized controlled trial to examine the efficacy of a web-based intervention with disaster-affected families." CONTEMPORARY CLINICAL TRIALS **40**: 138-149. Insufficient data to calculate effect size
50. Ruggiero, K. J., et al. (2015). "Web Intervention for Adolescents Affected by Disaster: Population-Based Randomized Controlled Trial." JOURNAL OF THE AMERICAN ACADEMY OF CHILD AND ADOLESCENT PSYCHIATRY **54**(9): 709-717. Not psychological interventions
51. Salloum, A. and S. Overstreet (2008). "Evaluation of individual and group grief and trauma interventions for children post disaster." JOURNAL OF CLINICAL CHILD AND ADOLESCENT PSYCHOLOGY 37(3): 495-507. Not RCTs
52. Salloum, A. and S. Overstreet (2012). "Grief and trauma intervention for children after disaster: Exploring coping skills versus trauma narration." BEHAVIOUR RESEARCH AND THERAPY **50**(3): 169-179. Not natural or man-made disasters
53. Scheeringa MS et al. Trauma-focused cognitive-behavioral therapy for posttraumatic stress disorder in three-through six year-old children: a randomized clinical trial. J CHILD PSYCHOL PSYCHIATRY ALLIED DISCIP. 2011;52(8):853–60 Not natural or man-made disasters
54. Shooshtary, M. H., et al. (2008). "Outcome of cognitive behavioral therapy in adolescents after natural disaster." JOURNAL OF ADOLESCENT HEALTH **42**(5): 466-472. Not RCTs
55. Slone, M., et al. (2013). "Helping Youth Immediately Following War Exposure: A Randomized Controlled Trial of a School-Based Intervention Program." JOURNAL OF PRIMARY PREVENTION **34**(5): 293-307. Not use PTSD or depression measurements.
56. Slone, M., Shoshani, A., & Lobel, T. (2013). Helping youth immediately following war exposure: A randomized controlled trial of a school-based intervention program. JOURNAL OF PRIMARY PREVENTION*, 34*(5), 293–307. Not use PTSD or depression measurements.
57. Smith, P., et al. (2007). "Cognitive-behavioral therapy for PTSD in children and adolescents: A preliminary randomized controlled trial." JOURNAL OF THE AMERICAN ACADEMY OF CHILD AND ADOLESCENT PSYCHIATRY **46**(8): 1051-1061. Not natural or man-made disasters
58. Smith, P., et al. (2022). "Therapist-supported online cognitive therapy for post-traumatic stress disorder (PTSD) in young people: protocol for an early-stage, parallel-group, randomised controlled study (OPTYC trial)." BMJ OPEN **12**(3). Insufficient data to calculate effect size
59. Sole, E. J., et al. (2017). "Anxiety and stress in children following an earthquake: Clinically beneficial effects of treatment with micronutrients." JOURNAL OF CHILD AND FAMILY STUDIES **26**(5): 1422-1431. Not RCTs
60. Stasiak, K., et al. (2018). "Delivering solid treatments on shaky ground: Feasibility study of an online therapy for child anxiety in the aftermath of a natural disaster." PSYCHOTHERAPY RESEARCH : JOURNAL OF THE SOCIETY FOR PSYCHOTHERAPY RESEARCH **28**(4): 643-653. Not RCTs
61. Suarez-Morales, L., et al. (2017). "Trauma in Hispanic Youth With Psychiatric Symptoms: Investigating Gender and Family Effects." PSYCHOLOGICAL TRAUMA-THEORY RESEARCH PRACTICE AND POLICY **9**(3): 334-343. Insufficient data to calculate effect size
62. Tang TC, Yang P, Yen CF, Liu TL (2015). Eye movement desensitization and reprocessing for treating psychological disturbances in Taiwanese adolescents who experienced Typhoon Morakot. KAOHSIUNG JOURNAL OF MEDICAL SCIENCES 31, 363–369 Not RCTs
63. Thabet AA, Vostanis P, Karim K (2005). Group crisis intervention for children during ongoing war conflict. EUROPEAN JOURNAL OF CHILD AND ADOLESCENT PSYCHIATRY 14, 262–269. Not RCTs
64. Tol WA, Komproe IH, Susanty D, Jordans MJD, Macy RD, de Jong JTVM. Schoolbased mental health intervention for children affected by political violence in Indonesia. A cluster randomized trial. JAMA. 2008;300(6):655–662. Insufficient data to calculate effect size
65. Tol, W. A., Komproe, I. H., Jordans, M. J. D., Ndayisaba, A., Ntamutumba, P., Sipsma, H., et al. (2014). School-based mental health intervention for children in war-affected Burundi: a cluster randomized trial. BMC MEDICINE*, 12*(1), 56. Insufficient data to calculate effect size
66. Tol, W. A., Komproe, I. H., Jordans, M. J. D., Vallipuram, A., Sipsma, H., Sivayokan, S., et al. (2012). Outcomes and moderators of a preventive school-based mental health intervention for children affected by war in Sri Lanka: A cluster randomized trial. WORLD PSYCHIATRY*, 11*(2), 114–122. Insufficient data to calculate effect size
67. Unterhitzenberger, J., & Rosner, R. (2014). Lessons from writing sessions: A school-based randomized trial with adolescent orphans in Rwanda. EUROPEAN JOURNAL OF PSYCHOTRAUMATOLOGY. Not psychological interventions
68. Valenti, M., et al. (2012). "A before and after study on personality assessment in adolescents exposed to the 2009 earthquake in L'Aquila, Italy: Influence of sports practice." BMJ OPEN **2**(3). Not RCTs
69. Vijayakumar L et al. Do all children need intervention after exposure to tsunami? INT REV PSYCHIATRY (Abingdon, England). 2006;18(6):515–22 Insufficient data to calculate effect size
70. Wadaa NN, Zaharim NM, Alqashan HF (2010). The use of EMDR in treatment of traumatized Iraqi children. DIGEST OF MIDDLE EAST STUDIES 19, 26–36. Not RCTs
71. Wang, Z., et al. (2013). "Chinese My Trauma Recovery, A Web-Based Intervention for Traumatized Persons in Two Parallel Samples: Randomized Controlled Trial." JOURNAL OF MEDICAL INTERNET RESEARCH **15**(9): 112-125. Not psychological interventions
72. Wang, Z., et al. (2016). "Program Use and Outcome Change in a Web-Based Trauma Intervention: Individual and Social Factors." JOURNAL OF MEDICAL INTERNET RESEARCH **18**(9). Not RCTs
73. Wolmer L et al. Teacher-mediated intervention after disaster: A controlled three-year follow-up of children's functioning. J Child Psychol Psychiatry. 2005;46(11):1161–8 Not RCTs
74. Wolmer, L., et al. (2011). "Teacher-Delivered Resilience-Focused Intervention in Schools With Traumatized Children Following the Second Lebanon War." JOURNAL OF TRAUMATIC STRESS **24**(3): 309-316. Not RCTs
75. Wolmer, L., Laor, N., & Yazgan, Y. (2003, April). School reactivation programs after disaster: Could teachers serve as clinical mediators? CHILD AND ADOLESCENT PSYCHIATRIC CLINICS OF NORTH AMERICA, 12(2), 363–381 Not RCTs
76. Zhang, Y., et al. (2011). "Clinical Study on Treatment of the Earthquake-caused Post-traumatic Stress Disorder by Cognitive-behavior Therapy and Acupoint Stimulation." JOURNAL OF TRADITIONAL CHINESE MEDICINE **31**(1): 60-63. Not children or adolescent

# Risk of bias of included studies

Risk of bias summary: review authors' judgements about each risk of bias item for each included study.


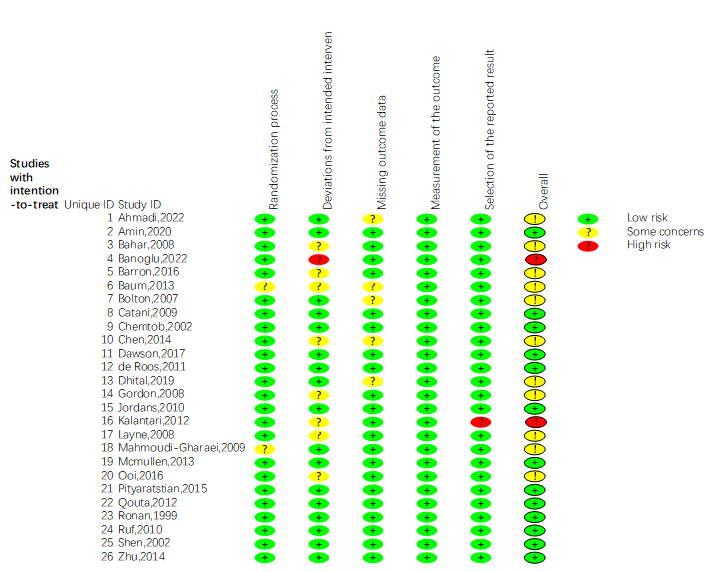


# Outcome1：PTSD - post

**Intervention codes：**

| WL/NT | A |
| --- | --- |
| TAU | B |
| ET | C |
| EMDR | D |
| CBT | E |
| PS | F |
| BRI | G |
| MBT | H |
| TGCT | I |
| PT | J |

Studies contributing to the analysis n = 30

**Network map**


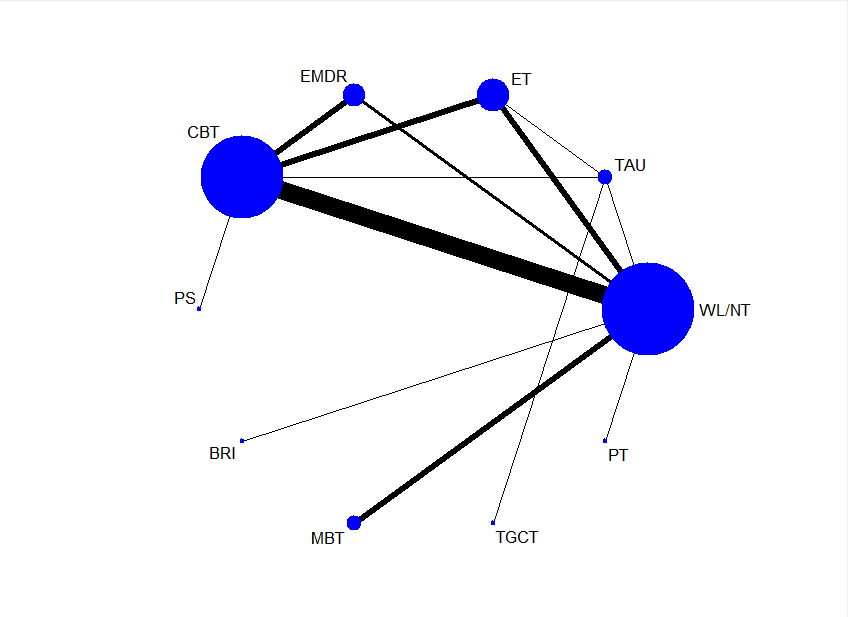


**Net league table**

| EMDR | 0.00 (-0.62,0.63) | 0.05 (-0.42,0.52) | 0.03 (-1.14,1.19) | 0.03 (-1.35,1.40) | 0.24 (-0.69,1.18) | 0.37 (-0.34,1.07) | 0.46 (-0.64,1.55) | 0.49 (-0.60,1.58) | 0.67 (0.17,1.17) |
| --- | --- | --- | --- | --- | --- | --- | --- | --- | --- |
| -0.00 (-0.63,0.62) | ET | 0.04 (-0.40,0.48) | 0.02 (-1.13,1.18) | 0.02 (-1.28,1.33) | 0.24 (-0.59,1.06) | 0.36 (-0.31,1.03) | 0.45 (-0.62,1.53) | 0.49 (-0.58,1.55) | 0.66 (0.22,1.11) |
| -0.05 (-0.52,0.42) | -0.04 (-0.48,0.40) | CBT | -0.02 (-1.09,1.05) | -0.02 (-1.33,1.28) | 0.19 (-0.63,1.02) | 0.32 (-0.26,0.89) | 0.41 (-0.61,1.43) | 0.44 (-0.57,1.46) | 0.62 (0.34,0.90) |
| -0.03 (-1.19,1.14) | -0.02 (-1.18,1.13) | 0.02 (-1.05,1.09) | PS | -0.00 (-1.69,1.69) | 0.21 (-1.14,1.57) | 0.34 (-0.88,1.55) | 0.43 (-1.05,1.91) | 0.46 (-1.01,1.94) | 0.64 (-0.46,1.75) |
| -0.03 (-1.40,1.35) | -0.02 (-1.33,1.28) | 0.02 (-1.28,1.33) | 0.00 (-1.69,1.69) | TGCT | 0.22 (-0.80,1.23) | 0.34 (-1.06,1.74) | 0.43 (-1.20,2.06) | 0.46 (-1.17,2.09) | 0.64 (-0.66,1.95) |
| -0.24 (-1.18,0.69) | -0.24 (-1.06,0.59) | -0.19 (-1.02,0.63) | -0.21 (-1.57,1.14) | -0.22 (-1.23,0.80) | TAU | 0.12 (-0.84,1.09) | 0.21 (-1.07,1.50) | 0.25 (-1.03,1.53) | 0.43 (-0.40,1.25) |
| -0.37 (-1.07,0.34) | -0.36 (-1.03,0.31) | -0.32 (-0.89,0.26) | -0.34 (-1.55,0.88) | -0.34 (-1.74,1.06) | -0.12 (-1.09,0.84) | MBT | 0.09 (-1.01,1.19) | 0.12 (-0.97,1.22) | 0.30 (-0.20,0.81) |
| -0.46 (-1.55,0.64) | -0.45 (-1.53,0.62) | -0.41 (-1.43,0.61) | -0.43 (-1.91,1.05) | -0.43 (-2.06,1.20) | -0.21 (-1.50,1.07) | -0.09 (-1.19,1.01) | BRI | 0.03 (-1.35,1.42) | 0.21 (-0.77,1.19) |
| -0.49 (-1.58,0.60) | -0.49 (-1.55,0.58) | -0.44 (-1.46,0.57) | -0.46 (-1.94,1.01) | -0.46 (-2.09,1.17) | -0.25 (-1.53,1.03) | -0.12 (-1.22,0.97) | -0.03 (-1.42,1.35) | PT | 0.18 (-0.79,1.15) |
| -0.67 (-1.17,-0.17) | -0.66 (-1.11,-0.22) | -0.62 (-0.90,-0.34) | -0.64 (-1.75,0.46) | -0.64 (-1.95,0.66) | -0.43 (-1.25,0.40) | -0.30 (-0.81,0.20) | -0.21 (-1.19,0.77) | -0.18 (-1.15,0.79) | WL/NT |

**Interval Plot**


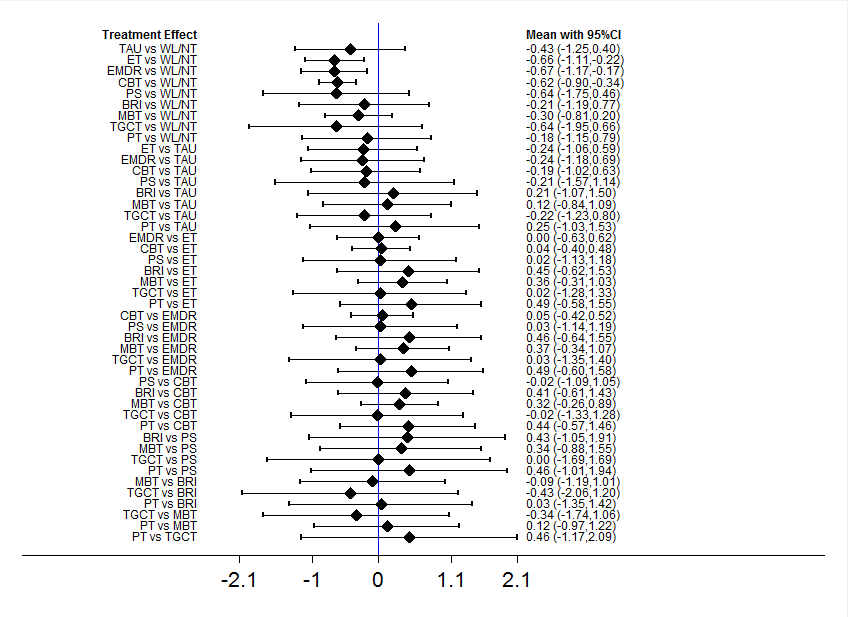


**Pairwise meta-analysis**

| WL/NT-CBT | SMD | [95% Conf. | Interval] |
| --- | --- | --- | --- |
| D+L pooled SMD | 0.783 | 0.331 | 1.236 |

**Test(s) of heterogeneity**

|  | Heterogeneity | d.f. | p | I-squared | Tau-squared |
| --- | --- | --- | --- | --- | --- |
| WL/NT-CBT | 85.05 | 8 | 0.000 | 90.6% | 0.413 |

**Evaluation of incoherence**

**Overall incoherence**

Design-by-treatment test: P= 0.960

**Loop-specific heterogeneity**

| Loop | IF | seIF | z_value | p_value | CI_95 | Loop_Heterog_tau2 |
| --- | --- | --- | --- | --- | --- | --- |
| A-B-C | 0.465 | 0.608 | 0.765 | 0.444 | (0.00,1.66) | 0.026 |
| B-C-E | 0.432 | 0.698 | 0.62 | 0.535 | (0.00,1.80) | 0.084 |
| A-D-E | 0.291 | 0.621 | 0.469 | 0.639 | (0.00,1.51) | 0.224 |
| A-C-E | 0.23 | 0.532 | 0.432 | 0.666 | (0.00,1.27) | 0.262 |
| A-B-E | 0.197 | 1.274 | 0.155 | 0.877 | (0.00,2.69) | 0.287 |

**Consistency between direct and indirect estimates**

| Side | Direct Coef. | Std. Err. | Indirect Coef. | Std. Err. | Difference Coef. | Std. Err. | P>\|z\| | tau |
| --- | --- | --- | --- | --- | --- | --- | --- | --- |
| A B | -0.163 | 0.641 | -0.639 | 0.573 | 0.476 | 0.860 | 0.580 | 0.494 |
| A C | -0.644 | 0.279 | -0.714 | 0.410 | 0.071 | 0.495 | 0.887 | 0.501 |
| A D | -0.484 | 0.417 | -0.785 | 0.327 | 0.301 | 0.530 | 0.570 | 0.496 |
| A E | -0.643 | 0.157 | -0.476 | 0.426 | -0.167 | 0.455 | 0.714 | 0.499 |
| A G | . | . | . | . | . | . | . | . |
| A H | . | . | . | . | . | . | . | . |
| A J | . | . | . | . | . | . | . | . |
| B C | -0.014 | 0.611 | -0.449 | 0.594 | 0.436 | 0.852 | 0.609 | 0.494 |
| B E | -0.294 | 0.627 | -0.112 | 0.577 | -0.182 | 0.847 | 0.830 | 0.497 |
| B I * | -0.216 | 0.516 | 0.863 | 63.263 | -1.080 | 63.265 | 0.986 | 0.485 |
| C E | 0.151 | 0.272 | -0.207 | 0.414 | 0.358 | 0.494 | 0.469 | 0.496 |
| D E | 0.137 | 0.289 | -0.164 | 0.445 | 0.302 | 0.530 | 0.570 | 0.496 |
| E F * | -0.021 | 0.545 | 1.243 | 63.254 | -1.264 | 63.257 | 0.984 | 0.485 |

**SUCRA and cumulative probability plots**

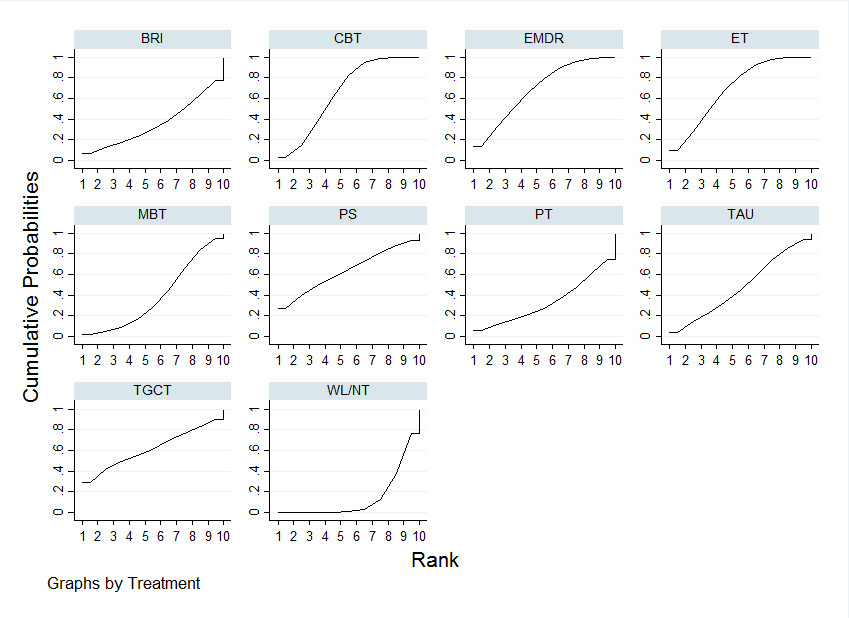


**Funnel Plot**


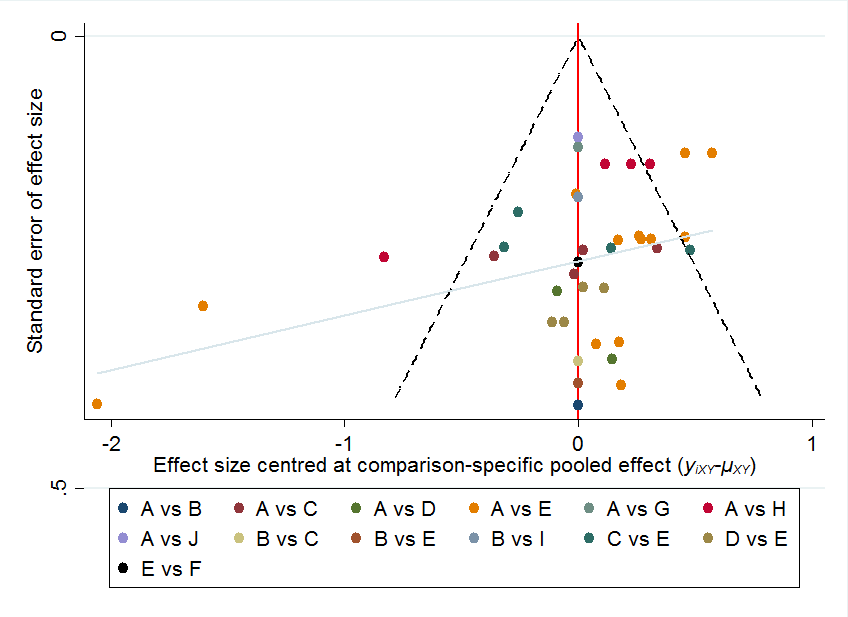


# Outcome2：PTSD - follow

**Intervention codes：**

| WL/NT | A |
| --- | --- |
| TAU | B |
| ET | C |
| EMDR | D |
| CBT | E |
| PS | F |
| PSS | G |
| TGCT | H |

Studies contributing to the analysis n = 18

**Network map**


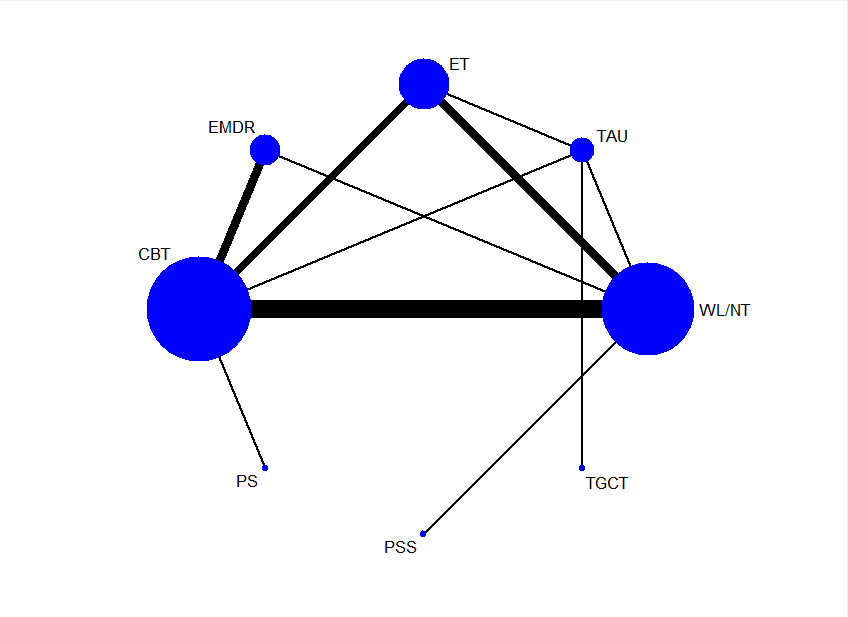


**Net league table**

| EMDR | 0.10 (-0.38,0.58) | 0.27 (-0.68,1.22) | 0.30 (-0.04,0.63) | 0.38 (-0.30,1.07) | 0.54 (-0.20,1.28) | 0.67 (0.07,1.27) | 0.72 (0.33,1.11) |
| --- | --- | --- | --- | --- | --- | --- | --- |
| -0.10 (-0.58,0.38) | ET | 0.17 (-0.72,1.06) | 0.19 (-0.15,0.54) | 0.28 (-0.32,0.88) | 0.44 (-0.31,1.19) | 0.57 (-0.00,1.14) | 0.62 (0.27,0.97) |
| -0.27 (-1.22,0.68) | -0.17 (-1.06,0.72) | TGCT | 0.02 (-0.87,0.92) | 0.11 (-0.55,0.77) | 0.27 (-0.84,1.38) | 0.40 (-0.60,1.40) | 0.45 (-0.44,1.34) |
| -0.30 (-0.63,0.04) | -0.19 (-0.54,0.15) | -0.02 (-0.92,0.87) | CBT | 0.09 (-0.52,0.69) | 0.25 (-0.41,0.91) | 0.38 (-0.14,0.89) | 0.43 (0.19,0.67) |
| -0.38 (-1.07,0.30) | -0.28 (-0.88,0.32) | -0.11 (-0.77,0.55) | -0.09 (-0.69,0.52) | TAU | 0.16 (-0.73,1.05) | 0.29 (-0.47,1.04) | 0.34 (-0.26,0.94) |
| -0.54 (-1.28,0.20) | -0.44 (-1.19,0.31) | -0.27 (-1.38,0.84) | -0.25 (-0.91,0.41) | -0.16 (-1.05,0.73) | PS | 0.13 (-0.71,0.97) | 0.18 (-0.52,0.88) |
| -0.67 (-1.27,-0.07) | -0.57 (-1.14,0.00) | -0.40 (-1.40,0.60) | -0.38 (-0.89,0.14) | -0.29 (-1.04,0.47) | -0.13 (-0.97,0.71) | PSS | 0.05 (-0.40,0.51) |
| -0.72 (-1.11,-0.33) | -0.62 (-0.97,-0.27) | -0.45 (-1.34,0.44) | -0.43 (-0.67,-0.19) | -0.34 (-0.94,0.26) | -0.18 (-0.88,0.52) | -0.05 (-0.51,0.40) | WL/NT |

**Interval Plot**


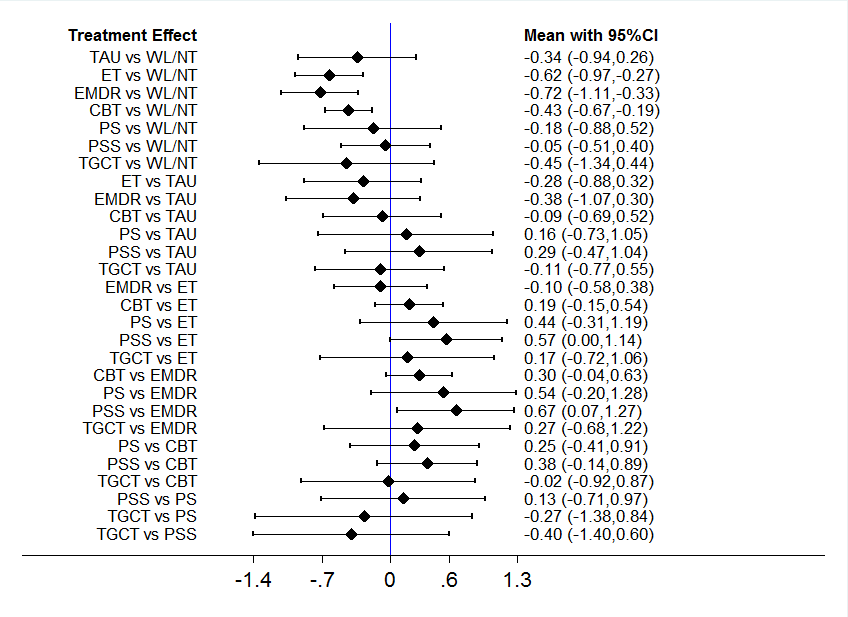


**Evaluation of incoherence**

**Overall incoherence**

Design-by-treatment test: P= 0.178

**Loop-specific heterogeneity**

| Loop | IF | seIF | z_value | p_value | CI_95 | Loop_Heterog_tau2 |
| --- | --- | --- | --- | --- | --- | --- |
| B-C-E | 1.267 | 0.559 | 2.265 | 0.024 | (0.17,2.36) | 0 |
| A-B-C | 0.851 | 0.604 | 1.407 | 0.159 | (0.00,2.04) | 0.018 |
| A-B-E | 0.63 | 0.656 | 0.96 | 0.337 | (0.00,1.91) | 0.046 |
| A-C-E | 0.243 | 0.317 | 0.769 | 0.442 | (0.00,0.86) | 0.044 |
| A-D-E | 0.232 | 0.48 | 0.482 | 0.63 | (0.00,1.17) | 0.051 |

**Consistency between direct and indirect estimates**

| Side | Direct Coef. | Std. Err. | Indirect Coef. | Std. Err. | Difference Coef. | Std. Err. | P>z | tau |
| --- | --- | --- | --- | --- | --- | --- | --- | --- |
| A B | -0.171 | 0.471 | -0.474 | 0.416 | 0.303 | 0.630 | 0.630 | 0.235 |
| A C | -0.781 | 0.187 | -0.037 | 0.346 | -0.744 | 0.393 | 0.058 | 0.177 |
| A D | -0.532 | 0.433 | -0.779 | 0.231 | 0.247 | 0.490 | 0.614 | 0.238 |
| A E | -0.436 | 0.130 | -0.383 | 0.431 | -0.053 | 0.450 | 0.906 | 0.238 |
| A G | . | . | . | . | . | . | . | . |
| B C | 0.252 | 0.414 | -0.838 | 0.425 | 1.090 | 0.593 | 0.066 | 0.203 |
| B E | -0.832 | 0.432 | 0.537 | 0.389 | -1.369 | 0.579 | 0.018 | 0.177 |
| B H * | -0.109 | 0.336 | 0.693 | 63.189 | -0.802 | 63.190 | 0.990 | 0.225 |
| C E | 0.176 | 0.214 | 0.233 | 0.336 | -0.058 | 0.395 | 0.884 | 0.238 |
| D E | 0.332 | 0.191 | 0.084 | 0.452 | 0.248 | 0.490 | 0.614 | 0.238 |
| E F * | 0.247 | 0.337 | 0.854 | 63.233 | -0.607 | 63.234 | 0.992 | 0.225 |

**SUCRA and cumulative probability plots**

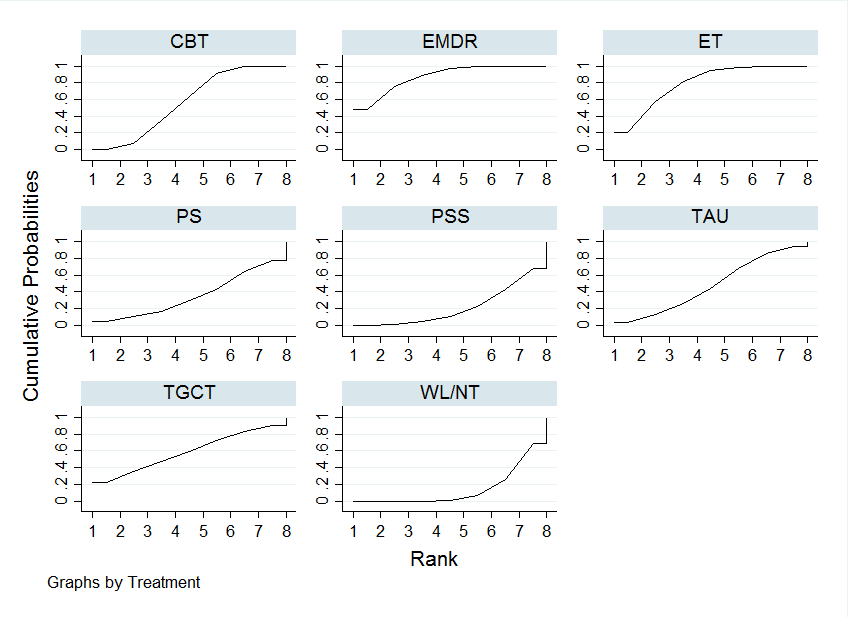


**Funnel Plot**


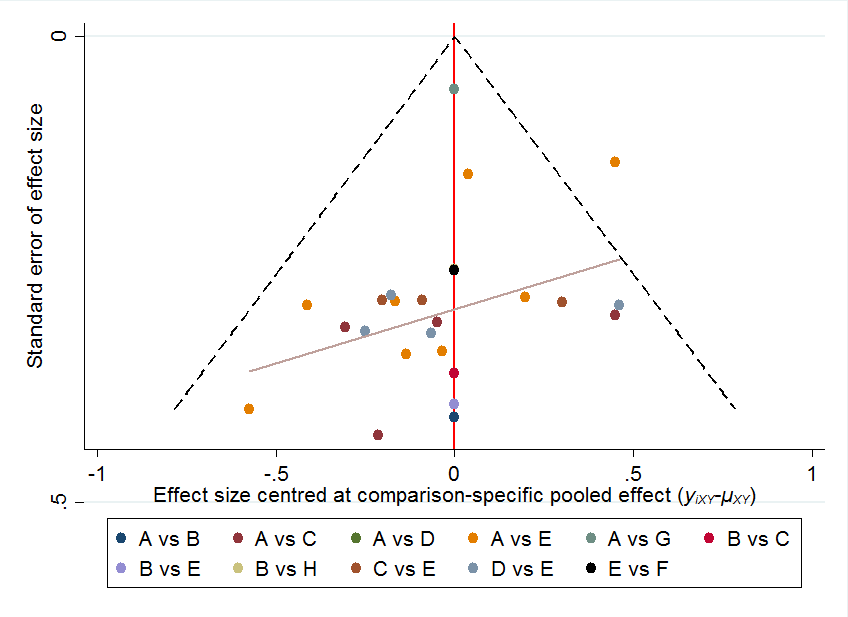


# Outcome3：Depression-post

| WL/NT | A |
| --- | --- |
| TAU | B |
| EMDR | C |
| CBT | D |
| PS | E |
| TGCT | F |
| PT | G |

Studies contributing to the analysis n = 13

**Network map**


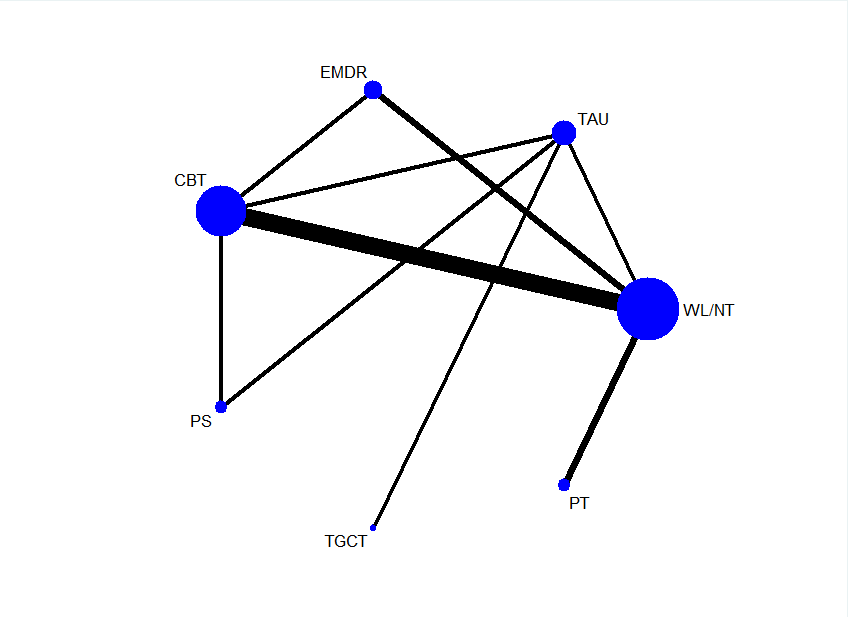


**Net league table**

| EMDR | 0.03 (-0.41,0.48) | 0.41 (-0.16,0.98) | 0.40 (0.01,0.79) | 0.40 (0.03,0.78) | 0.49 (-0.22,1.21) | 0.59 (-0.02,1.19) |
| --- | --- | --- | --- | --- | --- | --- |
| -0.03 (-0.48,0.41) | PT | 0.37 (-0.14,0.89) | 0.37 (0.07,0.67) | 0.37 (0.12,0.62) | 0.46 (-0.21,1.12) | 0.55 (0.00,1.10) |
| -0.41 (-0.98,0.16) | -0.37 (-0.89,0.14) | PS | -0.01 (-0.43,0.42) | -0.00 (-0.46,0.45) | 0.08 (-0.41,0.58) | 0.18 (-0.14,0.49) |
| -0.40 (-0.79,-0.01) | -0.37 (-0.67,-0.07) | 0.01 (-0.42,0.43) | CBT | 0.00 (-0.17,0.17) | 0.09 (-0.51,0.69) | 0.18 (-0.28,0.65) |
| -0.40 (-0.78,-0.03) | -0.37 (-0.62,-0.12) | 0.00 (-0.45,0.46) | -0.00 (-0.17,0.17) | WL/NT | 0.09 (-0.54,0.71) | 0.18 (-0.31,0.67) |
| -0.49 (-1.21,0.22) | -0.46 (-1.12,0.21) | -0.08 (-0.58,0.41) | -0.09 (-0.69,0.51) | -0.09 (-0.71,0.54) | TGCT | 0.10 (-0.28,0.48) |
| -0.59 (-1.19,0.02) | -0.55 (-1.10,-0.00) | -0.18 (-0.49,0.14) | -0.18 (-0.65,0.28) | -0.18 (-0.67,0.31) | -0.10 (-0.48,0.28) | TAU |

**Interval Plot**


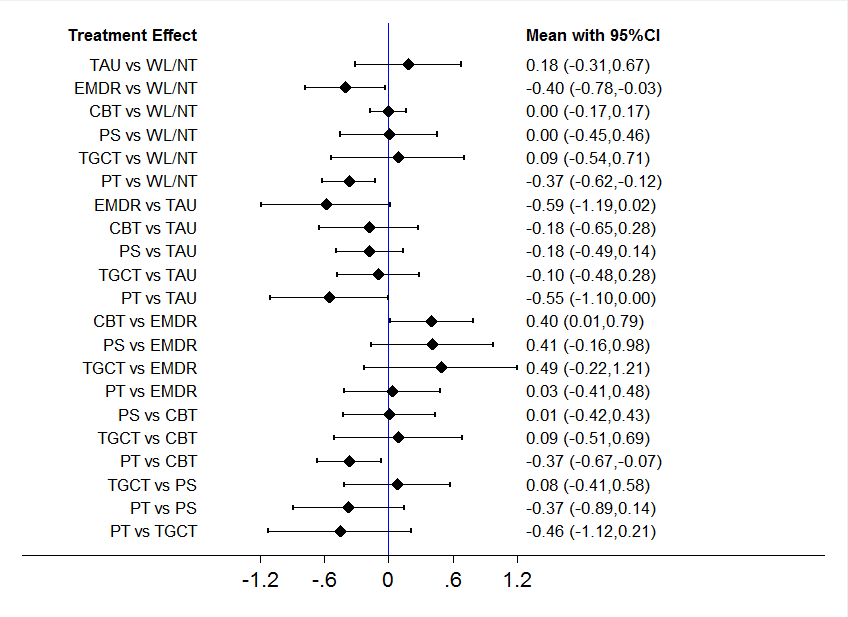


**Pairwise meta-analysis**

| WL/NT-CBT | SMD | [95% Conf. | Interval] |
| --- | --- | --- | --- |
| I-V pooled SMD | -0.035 | -0.183 | 0.114 |

**Test(s) of heterogeneity**

|  | Heterogeneity | d.f. | p | I-squared | Tau-squared |
| --- | --- | --- | --- | --- | --- |
| WL/NT-CBT | 2.47 | 3 | 0.482 | 0.0% | 0.000 |

**Evaluation of incoherence**

**Overall incoherence**

Design-by-treatment test: P= 0.175

**Loop-specific heterogeneity**

| Loop | IF | seIF | z_value | p_value | CI_95 | Loop_Heterog_tau2 |
| --- | --- | --- | --- | --- | --- | --- |
| A-C-D | 0.703 | 0.395 | 1.781 | 0.075 | (0.00,1.48) | 0 |
| B-D-E | 0.642 | 0.481 | 1.333 | 0.182 | (0.00,1.59) | 0 |
| A-B-D | 0.077 | 0.565 | 0.137 | 0.891 | (0.00,1.18) | 0 |

**Consistency between direct and indirect estimates**

| Side | Direct Coef. | Std. Err. | Indirect Coef. | Std. Err. | Difference Coef. | Std. Err. | P>z | tau |
| --- | --- | --- | --- | --- | --- | --- | --- | --- |
| A B | -0.085 | 0.414 | 0.322 | 0.300 | -0.408 | 0.511 | 0.425 | 0.066 |
| A C | -0.627 | 0.224 | 0.069 | 0.326 | -0.696 | 0.396 | 0.079 | 0.015 |
| A D | 0.038 | 0.074 | -0.746 | 0.356 | 0.783 | 0.364 | 0.031 | 0.000 |
| A G | . | . | . | . | . | . | . | . |
| B D | 0.184 | 0.388 | -0.379 | 0.283 | 0.563 | 0.473 | 0.234 | 0.067 |
| B E | -0.254 | 0.161 | 0.343 | 0.435 | -0.597 | 0.464 | 0.198 | 0.057 |
| B F * | -0.095 | 0.194 | -0.360 | 63.143 | 0.265 | 63.143 | 0.997 | 0.074 |
| C D | -0.038 | 0.317 | 0.657 | 0.237 | -0.696 | 0.396 | 0.079 | 0.015 |
| D E | 0.190 | 0.257 | -0.407 | 0.386 | 0.597 | 0.464 | 0.198 | 0.057 |

**SUCRA and cumulative probability plots**

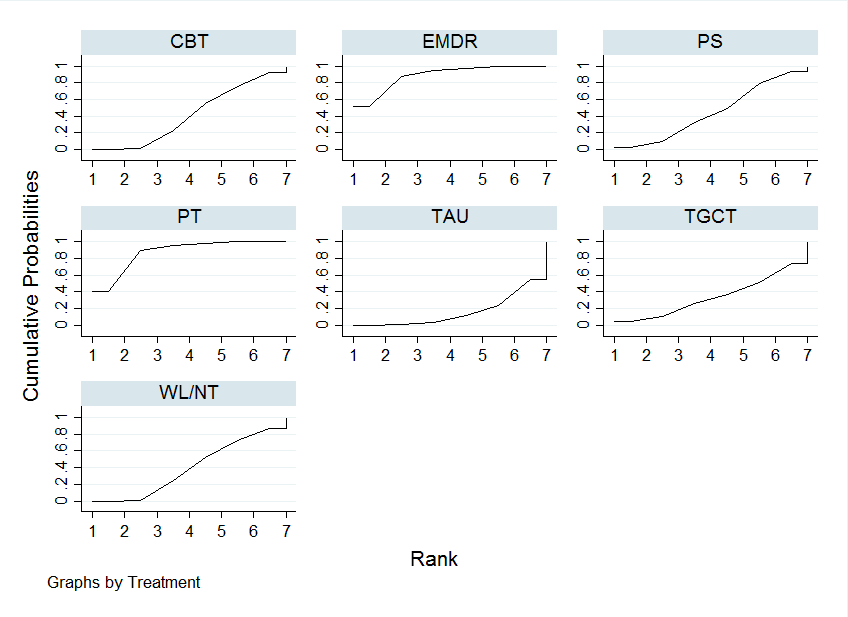


**Funnel Plot**


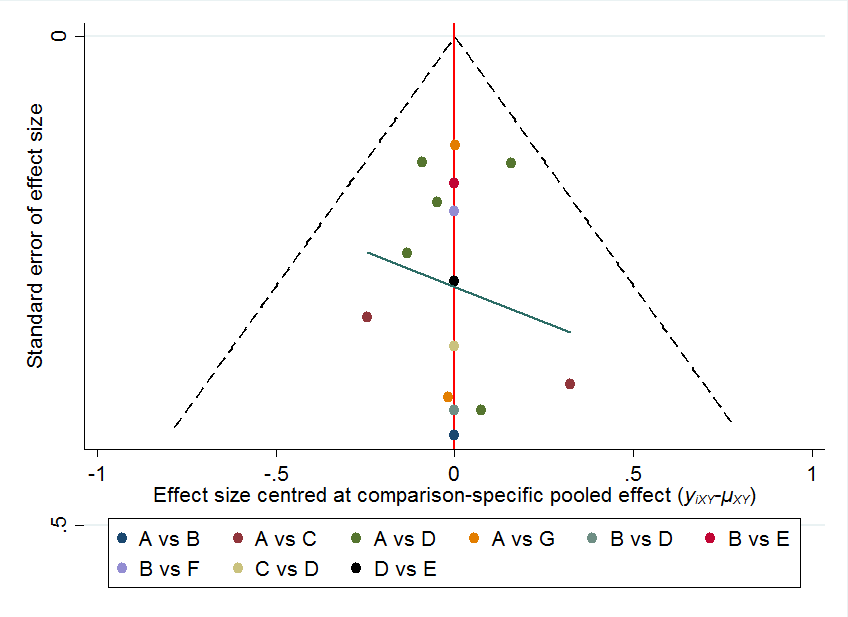


# Outcome4：Depression- follow

| WL/NT | A |
| --- | --- |
| TAU | B |
| EMDR | C |
| CBT | D |
| PT | E |
| IPT | F |
| PS | G |
| PSS | H |
| TGCT | I |

Studies contributing to the analysis n = 9

**Network map**


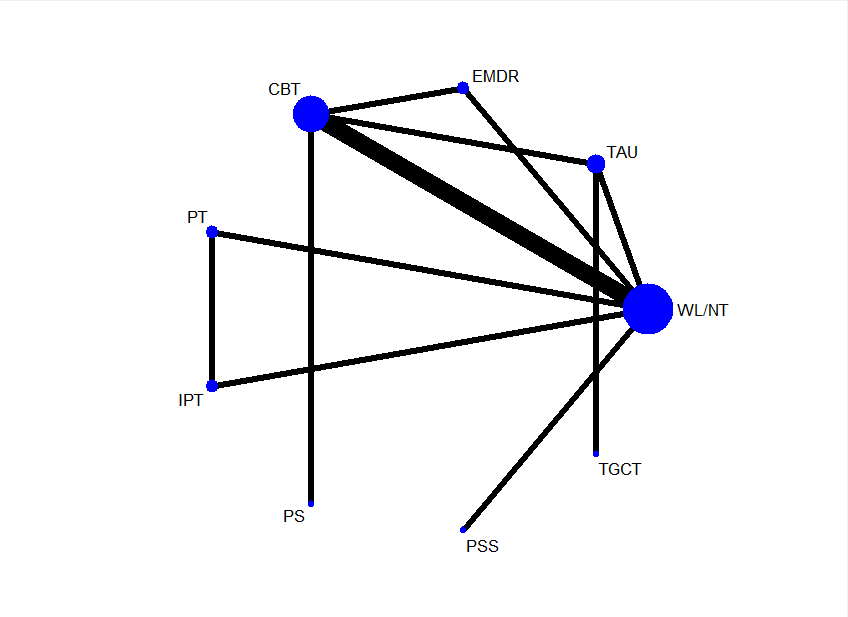


**Net league table**

| IPT | 0.31 (-1.05,1.68) | 0.44 (-1.10,1.98) | 0.51 (-0.69,1.71) | 0.50 (-1.39,2.40) | 0.63 (-1.00,2.26) | 0.72 (-0.71,2.15) | 0.69 (-0.34,1.73) | 0.91 (-0.13,1.95) |
| --- | --- | --- | --- | --- | --- | --- | --- | --- |
| -0.31 (-1.68,1.05) | EMDR | 0.13 (-1.25,1.51) | 0.20 (-0.70,1.09) | 0.19 (-1.58,1.95) | 0.31 (-1.10,1.73) | 0.41 (-0.93,1.75) | 0.38 (-0.52,1.28) | 0.60 (-0.77,1.96) |
| -0.44 (-1.98,1.10) | -0.13 (-1.51,1.25) | TAU | 0.07 (-1.06,1.20) | 0.06 (-1.04,1.16) | 0.19 (-1.39,1.77) | 0.28 (-1.24,1.80) | 0.25 (-0.89,1.40) | 0.47 (-1.07,2.01) |
| -0.51 (-1.71,0.69) | -0.20 (-1.09,0.70) | -0.07 (-1.20,1.06) | CBT | -0.01 (-1.58,1.57) | 0.12 (-0.98,1.22) | 0.21 (-0.96,1.38) | 0.18 (-0.43,0.80) | 0.40 (-0.80,1.60) |
| -0.50 (-2.40,1.39) | -0.19 (-1.95,1.58) | -0.06 (-1.16,1.04) | 0.01 (-1.57,1.58) | TGCT | 0.13 (-1.80,2.05) | 0.22 (-1.66,2.09) | 0.19 (-1.40,1.78) | 0.41 (-1.49,2.30) |
| -0.63 (-2.26,1.00) | -0.31 (-1.73,1.10) | -0.19 (-1.77,1.39) | -0.12 (-1.22,0.98) | -0.13 (-2.05,1.80) | PS | 0.09 (-1.52,1.70) | 0.06 (-1.20,1.33) | 0.28 (-1.35,1.91) |
| -0.72 (-2.15,0.71) | -0.41 (-1.75,0.93) | -0.28 (-1.80,1.24) | -0.21 (-1.38,0.96) | -0.22 (-2.09,1.66) | -0.09 (-1.70,1.52) | PSS | -0.03 (-1.02,0.97) | 0.19 (-1.24,1.62) |
| -0.69 (-1.73,0.34) | -0.38 (-1.28,0.52) | -0.25 (-1.40,0.89) | -0.18 (-0.80,0.43) | -0.19 (-1.78,1.40) | -0.06 (-1.33,1.20) | 0.03 (-0.97,1.02) | WL/NT | 0.22 (-0.82,1.25) |
| -0.91 (-1.95,0.13) | -0.60 (-1.96,0.77) | -0.47 (-2.01,1.07) | -0.40 (-1.60,0.80) | -0.41 (-2.30,1.49) | -0.28 (-1.91,1.35) | -0.19 (-1.62,1.24) | -0.22 (-1.25,0.82) | PT |

**Interval Plot**


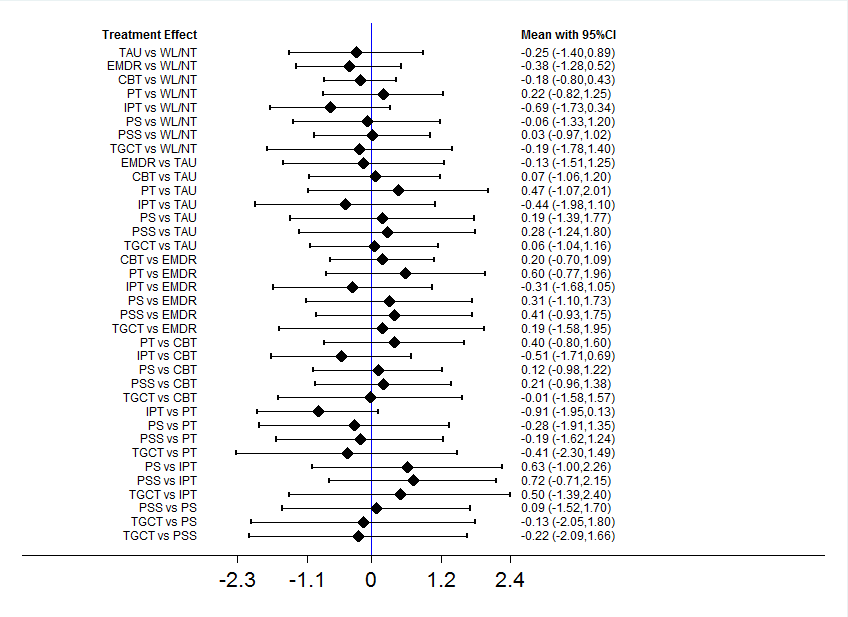


**Evaluation of incoherence**

**Overall incoherence**

Design-by-treatment test: P= 0.005

**Loop-specific heterogeneity**

| Loop | IF | seIF | z_value | p_value | CI_95 | Loop_Heterog_tau2 |
| --- | --- | --- | --- | --- | --- | --- |
| A-B-D | 1.362 | 0.579 | 2.353 | 0.019 | (0.23,2.50) | 0 |
| A-C-D | 0.349 | 1.171 | 0.298 | 0.766 | (0.00,2.64) | 0.144 |
| A-E-F | . | . | . | . |  | 0 |

**Consistency between direct and indirect estimates**

| Side | Direct Coef. | Std. Err. | Indirect Coef. | Std. Err. | Difference Coef. | Std. Err. | P>z | tau |
| --- | --- | --- | --- | --- | --- | --- | --- | --- |
| A B * | -0.829 | 0.420 | 1.899 | 0.704 | -2.728 | 0.844 | 0.001 | 0.000 |
| A C | -0.199 | 0.752 | -0.638 | 0.846 | 0.439 | 1.132 | 0.698 | 0.663 |
| A D * | -0.273 | 0.416 | 0.165 | 1.051 | -0.439 | 1.131 | 0.698 | 0.663 |
| A E | . | . | . | . | . | . | . | . |
| A F | . | . | . | . | . | . | . | . |
| A H | . | . | . | . | . | . | . | . |
| B D * | -0.434 | 0.385 | 2.294 | 0.761 | -2.727 | 0.843 | 0.001 | 0.000 |
| B I * | 0.063 | 0.562 | 0.503 | 63.269 | -0.440 | 63.272 | 0.994 | 0.504 |
| C D | 0.365 | 0.736 | -0.074 | 0.860 | 0.439 | 1.132 | 0.698 | 0.663 |
| D G * | 0.119 | 0.563 | 0.368 | 63.251 | -0.249 | 63.253 | 0.997 | 0.504 |
| E F | . | . | . | . | . | . | . | . |

**SUCRA and cumulative probability plots**

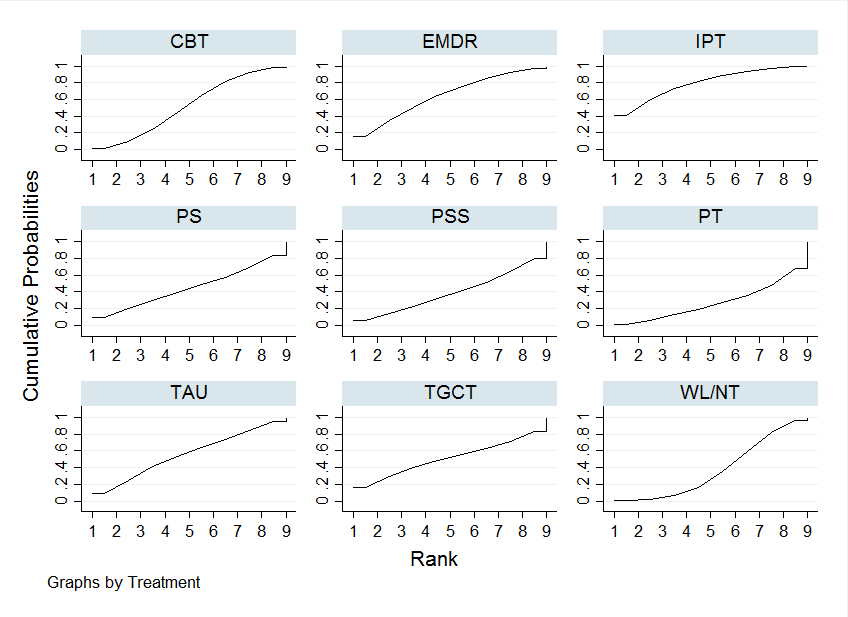


**Funnel Plot**


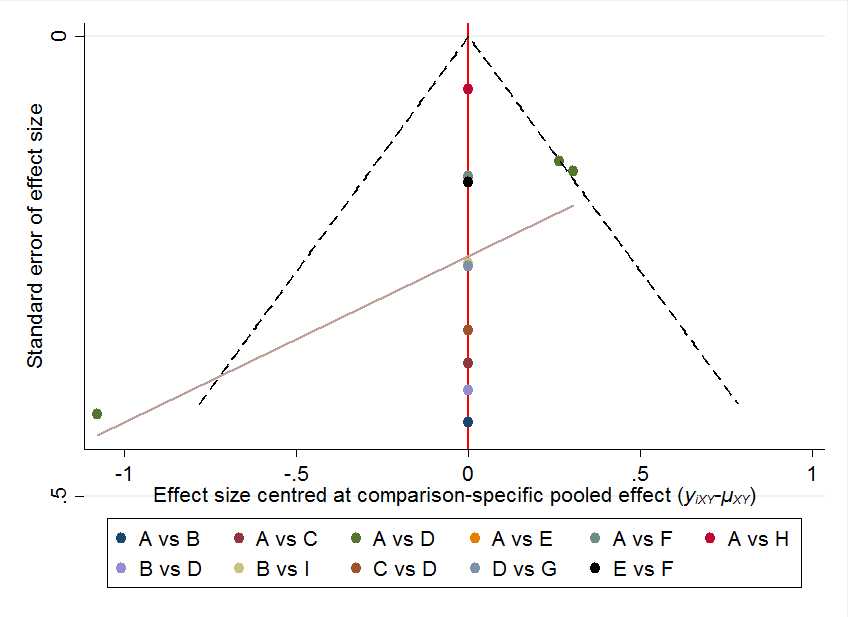


# Sensitivity analysis: excluding trials with a high risk of bias

**Intervention codes：**

| WL/NT | A |
| --- | --- |
| TAU | B |
| ET | C |
| EMDR | D |
| CBT | E |
| PS | F |
| BRI | G |
| MBT | H |
| TGCT | I |
| PT | J |

Studies contributing to the analysis n = 28

**Network map**


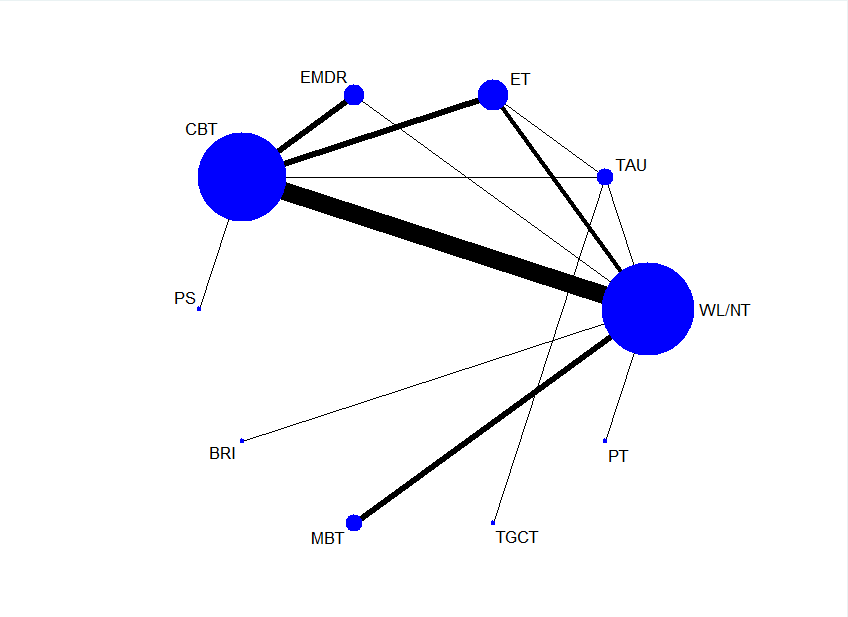


**Net league table**

| EMDR | 0.02 (-0.69,0.73) | 0.06 (-0.47,0.59) | 0.04 (-1.20,1.28) | 0.04 (-1.42,1.51) | 0.26 (-0.74,1.26) | 0.39 (-0.40,1.17) | 0.48 (-0.71,1.67) | 0.51 (-0.67,1.70) | 0.69 (0.11,1.27) |
| --- | --- | --- | --- | --- | --- | --- | --- | --- | --- |
| -0.02 (-0.73,0.69) | ET | 0.04 (-0.45,0.53) | 0.02 (-1.21,1.24) | 0.02 (-1.35,1.40) | 0.24 (-0.63,1.10) | 0.36 (-0.37,1.10) | 0.46 (-0.70,1.61) | 0.49 (-0.66,1.64) | 0.67 (0.16,1.18) |
| -0.06 (-0.59,0.47) | -0.04 (-0.53,0.45) | CBT | -0.02 (-1.14,1.10) | -0.02 (-1.39,1.35) | 0.20 (-0.66,1.06) | 0.32 (-0.29,0.94) | 0.42 (-0.67,1.50) | 0.45 (-0.63,1.53) | 0.63 (0.33,0.94) |
| -0.04 (-1.28,1.20) | -0.02 (-1.24,1.21) | 0.02 (-1.10,1.14) | PS | 0.00 (-1.77,1.78) | 0.22 (-1.20,1.63) | 0.34 (-0.93,1.62) | 0.44 (-1.12,2.00) | 0.47 (-1.09,2.03) | 0.65 (-0.51,1.82) |
| -0.04 (-1.51,1.42) | -0.02 (-1.40,1.35) | 0.02 (-1.35,1.39) | -0.00 (-1.78,1.77) | TGCT | 0.22 (-0.85,1.29) | 0.34 (-1.13,1.82) | 0.43 (-1.29,2.16) | 0.47 (-1.25,2.19) | 0.65 (-0.73,2.02) |
| -0.26 (-1.26,0.74) | -0.24 (-1.10,0.63) | -0.20 (-1.06,0.66) | -0.22 (-1.63,1.20) | -0.22 (-1.29,0.85) | TAU | 0.13 (-0.89,1.14) | 0.22 (-1.14,1.57) | 0.25 (-1.10,1.60) | 0.43 (-0.44,1.30) |
| -0.39 (-1.17,0.40) | -0.36 (-1.10,0.37) | -0.32 (-0.94,0.29) | -0.34 (-1.62,0.93) | -0.34 (-1.82,1.13) | -0.13 (-1.14,0.89) | MBT | 0.09 (-1.07,1.26) | 0.13 (-1.04,1.29) | 0.31 (-0.23,0.84) |
| -0.48 (-1.67,0.71) | -0.46 (-1.61,0.70) | -0.42 (-1.50,0.67) | -0.44 (-2.00,1.12) | -0.43 (-2.16,1.29) | -0.22 (-1.57,1.14) | -0.09 (-1.26,1.07) | BRI | 0.03 (-1.43,1.50) | 0.21 (-0.83,1.25) |
| -0.51 (-1.70,0.67) | -0.49 (-1.64,0.66) | -0.45 (-1.53,0.63) | -0.47 (-2.03,1.09) | -0.47 (-2.19,1.25) | -0.25 (-1.60,1.10) | -0.13 (-1.29,1.04) | -0.03 (-1.50,1.43) | PT | 0.18 (-0.85,1.21) |
| -0.69 (-1.27,-0.11) | -0.67 (-1.18,-0.16) | -0.63 (-0.94,-0.33) | -0.65 (-1.82,0.51) | -0.65 (-2.02,0.73) | -0.43 (-1.30,0.44) | -0.31 (-0.84,0.23) | -0.21 (-1.25,0.83) | -0.18 (-1.21,0.85) | WL/NT |

**Interval Plot**


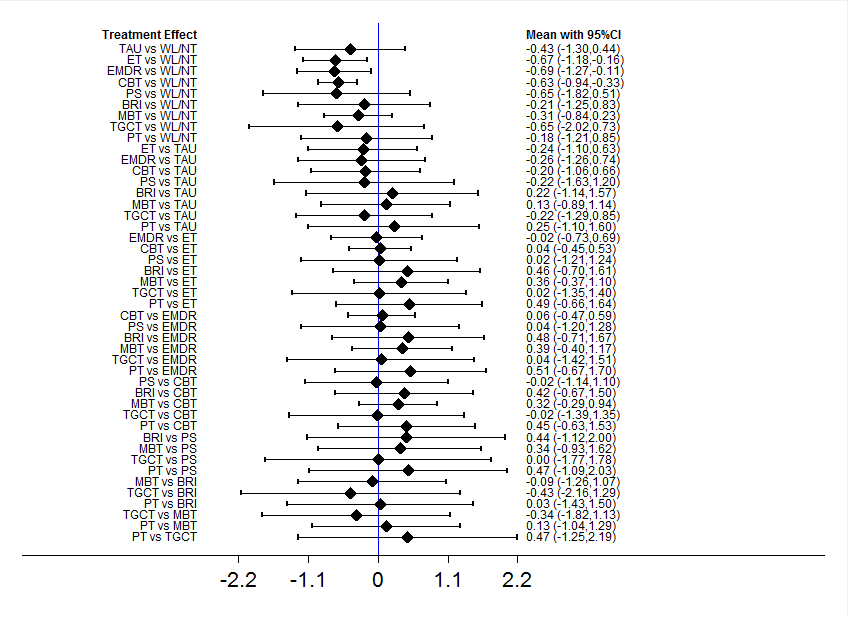


**Evaluation of incoherence**

**Overall incoherence**

Design-by-treatment test: P= 0.915

**Loop-specific heterogeneity**

| Loop | IF | seIF | z_value | p_value | CI_95 | Loop_Heterog_tau2 |
| --- | --- | --- | --- | --- | --- | --- |
| A-B-C | 0.461 | 0.685 | 0.674 | 0.5 | (0.00,1.80) | 0.065 |
| B-C-E | 0.432 | 0.698 | 0.62 | 0.535 | (0.00,1.80) | 0.084 |
| A-D-E | 0.42 | 0.838 | 0.501 | 0.616 | (0.00,2.06) | 0.234 |
| A-C-E | 0.238 | 0.589 | 0.405 | 0.686 | (0.00,1.39) | 0.279 |
| A-B-E | 0.197 | 1.274 | 0.155 | 0.877 | (0.00,2.69) | 0.287 |

**Consistency between direct and indirect estimates**

| Side | Direct Coef. | Std. Err. | Indirect Coef. | Std. Err. | Difference Coef. | Std. Err. | P>\|z\| | tau |
| --- | --- | --- | --- | --- | --- | --- | --- | --- |
| A B | -0.163 | 0.666 | -0.656 | 0.607 | 0.493 | 0.902 | 0.585 | 0.526 |
| A C | -0.639 | 0.337 | -0.727 | 0.434 | 0.088 | 0.548 | 0.872 | 0.533 |
| A D | -0.357 | 0.636 | -0.790 | 0.343 | 0.433 | 0.723 | 0.549 | 0.527 |
| A E | -0.648 | 0.165 | -0.409 | 0.617 | -0.240 | 0.639 | 0.707 | 0.531 |
| A G | . | . | . | . | . | . | . | . |
| A H | . | . | . | . | . | . | . | . |
| A J | . | . | . | . | . | . | . | . |
| B C | -0.014 | 0.638 | -0.458 | 0.628 | 0.444 | 0.895 | 0.620 | 0.527 |
| B E | -0.294 | 0.654 | -0.119 | 0.606 | -0.175 | 0.886 | 0.843 | 0.529 |
| B I * | -0.216 | 0.546 | 0.872 | 63.262 | -1.089 | 63.264 | 0.986 | 0.516 |
| C E | 0.151 | 0.285 | -0.368 | 0.546 | 0.519 | 0.614 | 0.398 | 0.525 |
| D E | 0.137 | 0.302 | -0.297 | 0.657 | 0.434 | 0.723 | 0.548 | 0.527 |
| E F * | -0.021 | 0.573 | 1.261 | 63.256 | -1.282 | 63.259 | 0.984 | 0.516 |

**SUCRA and cumulative probability plots**

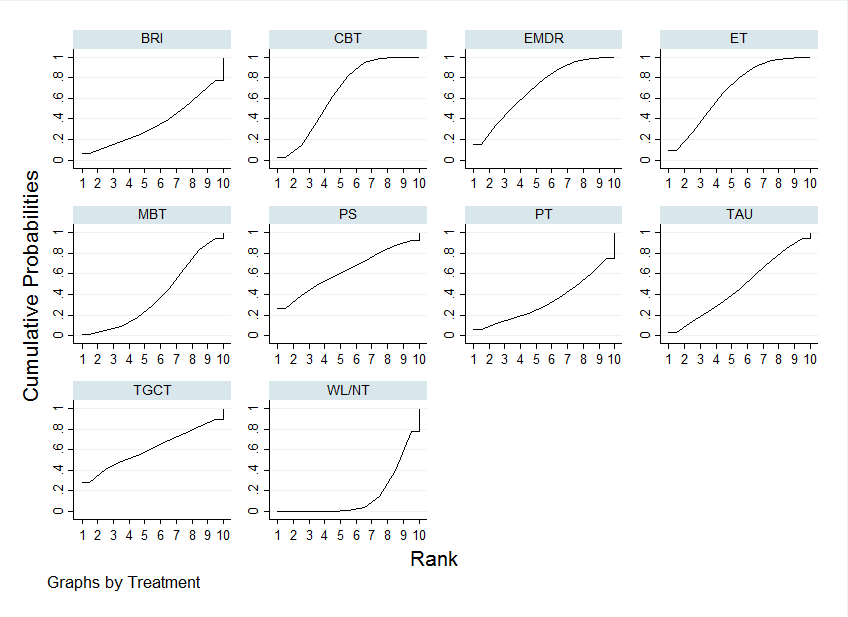


**Funnel Plot**


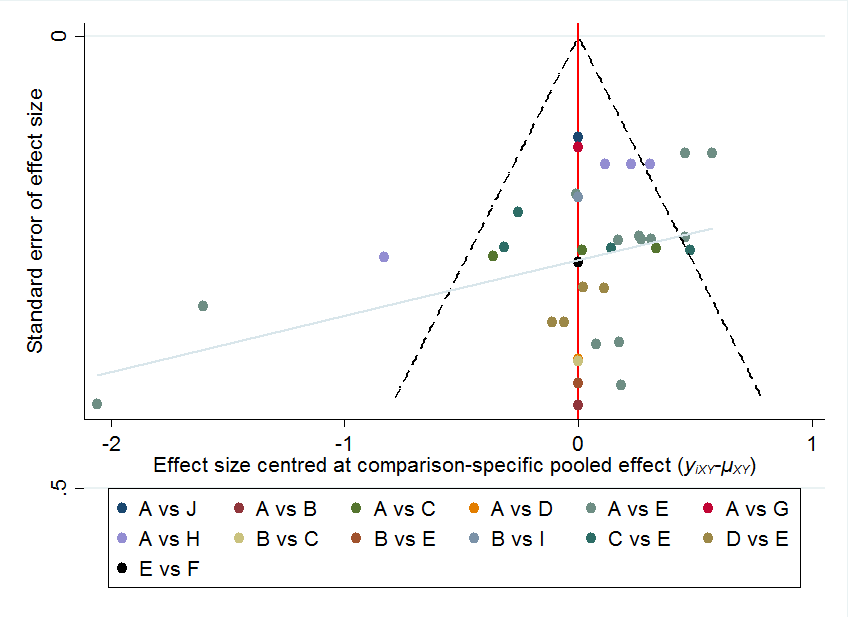


# Subgroup analysis : Level of Intervention

**PTSD-post**

Group n = 22 incoherence

Individual n=8

| WL/NT | A |
| --- | --- |
| ET | B |
| CBT | C |
| PS | D |
| TAU | E |
| EMDR | F |

**Network map**


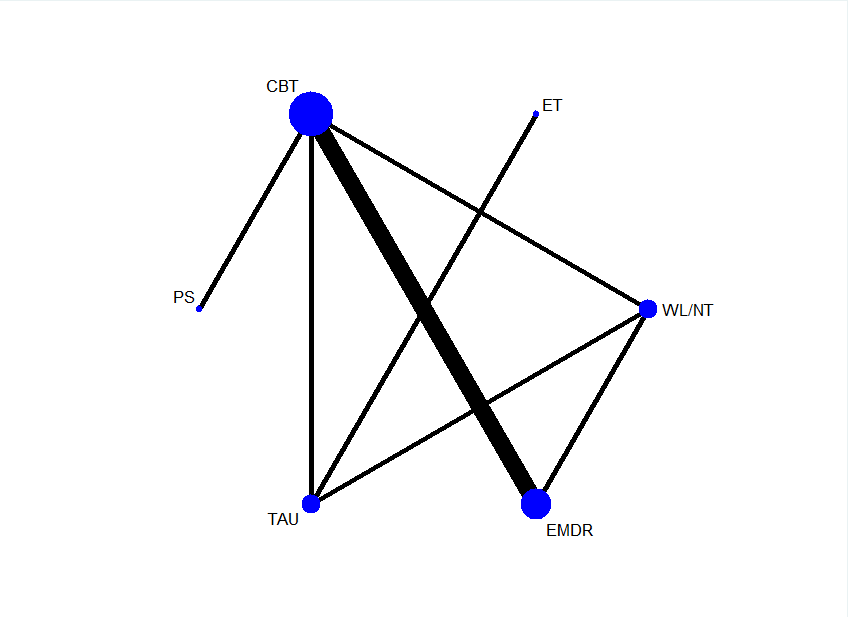


**Net league table**

| EMDR | 0.10 (-0.46,0.67) | 0.13 (-0.15,0.40) | 0.36 (-0.67,1.38) | 0.37 (-0.37,1.12) | 0.46 (-0.07,0.99) |
| --- | --- | --- | --- | --- | --- |
| -0.10 (-0.67,0.46) | PS | 0.02 (-0.47,0.51) | 0.25 (-0.86,1.37) | 0.27 (-0.60,1.13) | 0.36 (-0.37,1.09) |
| -0.13 (-0.40,0.15) | -0.02 (-0.51,0.47) | CBT | 0.23 (-0.77,1.24) | 0.25 (-0.47,0.96) | 0.34 (-0.20,0.87) |
| -0.36 (-1.38,0.67) | -0.25 (-1.37,0.86) | -0.23 (-1.24,0.77) | ET | 0.01 (-0.69,0.72) | 0.11 (-0.92,1.13) |
| -0.37 (-1.12,0.37) | -0.27 (-1.13,0.60) | -0.25 (-0.96,0.47) | -0.01 (-0.72,0.69) | TAU | 0.09 (-0.65,0.83) |
| -0.46 (-0.99,0.07) | -0.36 (-1.09,0.37) | -0.34 (-0.87,0.20) | -0.11 (-1.13,0.92) | -0.09 (-0.83,0.65) | WL/NT |

**Interval Plot**


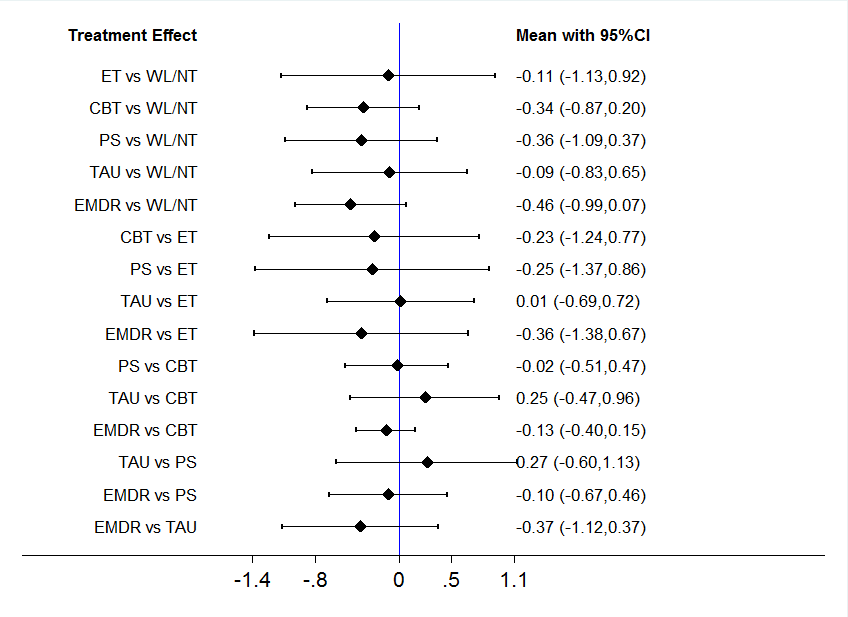


**Evaluation of incoherence**

**Overall incoherence**

Design-by-treatment test: P= 0.646

**Loop-specific heterogeneity**

| Loop | IF | seIF | z_value | p_value | CI_95 | Loop_Heterog_tau2 |
| --- | --- | --- | --- | --- | --- | --- |
| A-C-F | 0.251 | 0.546 | 0.460 | 0.645 | (0.00,1.32) | 0.000 |
| A-C-E | . | . | . | . |  | 0.000 |

**Consistency between direct and indirect estimates**

| Side | Direct Coef. | Std. Err. | Indirect Coef. | Std. Err. | Difference Coef. | Std. Err. | P>\|z\| | tau |
| --- | --- | --- | --- | --- | --- | --- | --- | --- |
| A C * | (0.464) | 0.386 | (0.212) | 0.387 | (0.252) | 0.546 | 0.645 | 0.125 |
| A E * | (0.163) | 0.409 | 0.340 | 1.011 | (0.503) | 1.092 | 0.645 | 0.846 |
| A F | (0.357) | 0.357 | (0.608) | 0.413 | 0.251 | 0.546 | 0.646 | 1.560 |
| B E * | 0.014 | 0.359 | (0.184) | 63.256 | 0.198 | 63.257 | 0.998 | 1.290 |
| C D * | (0.021) | 0.250 | 0.677 | 63.266 | (0.698) | 63.266 | 0.991 | 0.552 |
| C E * | 0.301 | 0.384 | (0.202) | 1.040 | 0.503 | 1.092 | 0.645 | 0.151 |
| C F | (0.144) | 0.148 | 0.107 | 0.526 | (0.251) | 0.546 | 0.645 | 0.000 |

**SUCRA and cumulative probability plots**


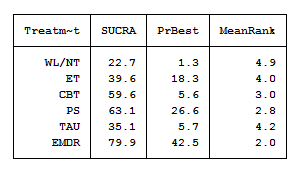


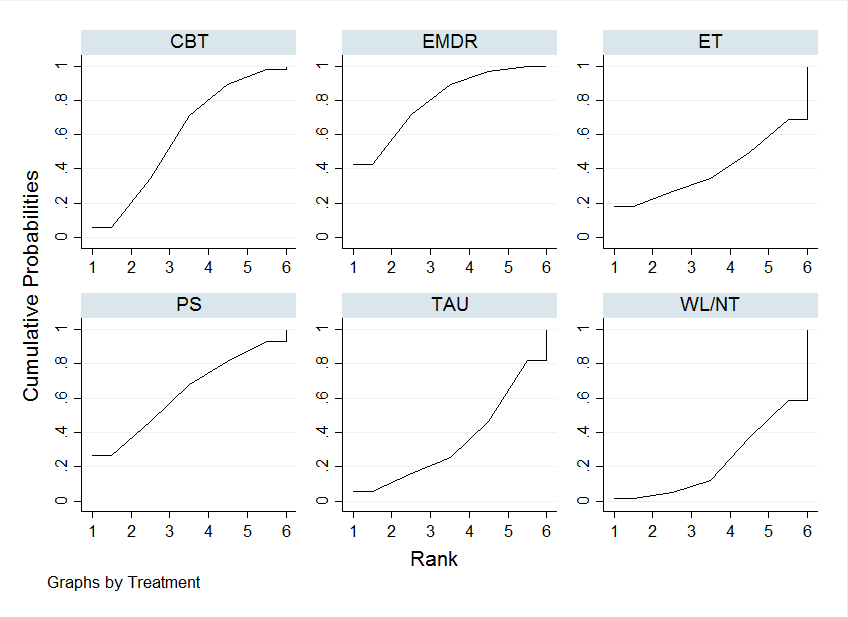


**Funnel Plot**


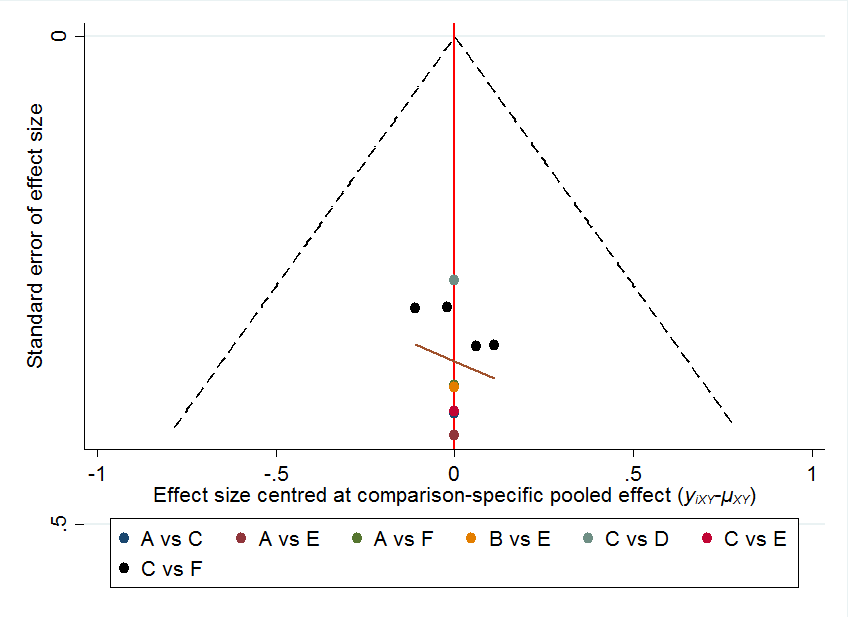


PTSD- follow：

Group n = 7 incoherence

Individual n=8

| WL/NT | A |
| --- | --- |
| ET | B |
| CBT | C |
| PS | D |
| TAU | E |
| EMDR | F |

**Network map**


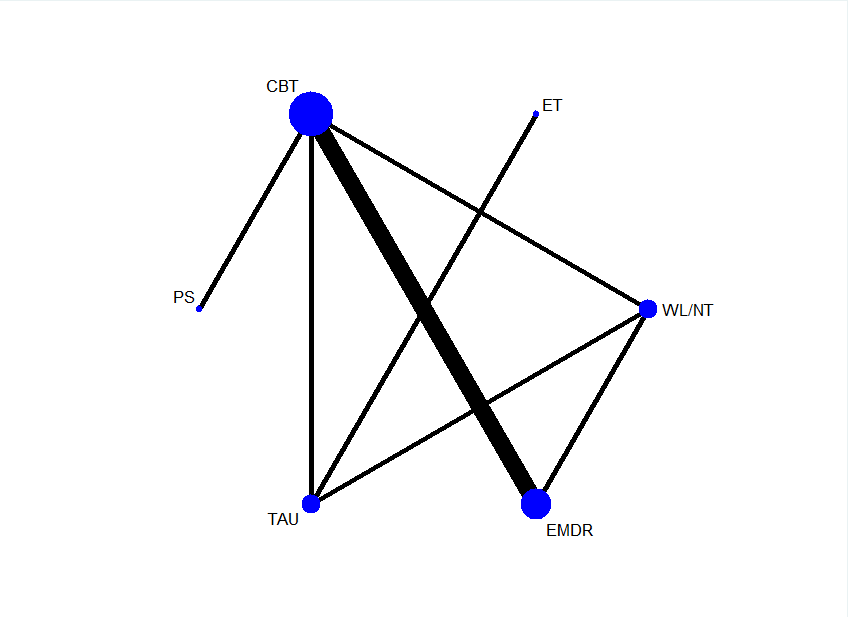


**Net league table**

| EMDR | 0.27 (-0.07,0.61) | 0.51 (-0.20,1.23) | 0.91 (0.06,1.76) | 0.88 (0.26,1.49) | 1.16 (-0.02,2.34) |
| --- | --- | --- | --- | --- | --- |
| -0.27 (-0.61,0.07) | CBT | 0.25 (-0.38,0.88) | 0.64 (-0.18,1.46) | 0.61 (-0.02,1.24) | 0.89 (-0.26,2.05) |
| -0.51 (-1.23,0.20) | -0.25 (-0.88,0.38) | PS | 0.39 (-0.64,1.43) | 0.36 (-0.53,1.25) | 0.65 (-0.67,1.96) |
| -0.91 (-1.76,-0.06) | -0.64 (-1.46,0.18) | -0.39 (-1.43,0.64) | TAU | -0.03 (-0.86,0.80) | 0.25 (-0.56,1.06) |
| -0.88 (-1.49,-0.26) | -0.61 (-1.24,0.02) | -0.36 (-1.25,0.53) | 0.03 (-0.80,0.86) | WL/NT | 0.28 (-0.88,1.45) |
| -1.16 (-2.34,0.02) | -0.89 (-2.05,0.26) | -0.65 (-1.96,0.67) | -0.25 (-1.06,0.56) | -0.28 (-1.45,0.88) | ET |

**Interval Plot**


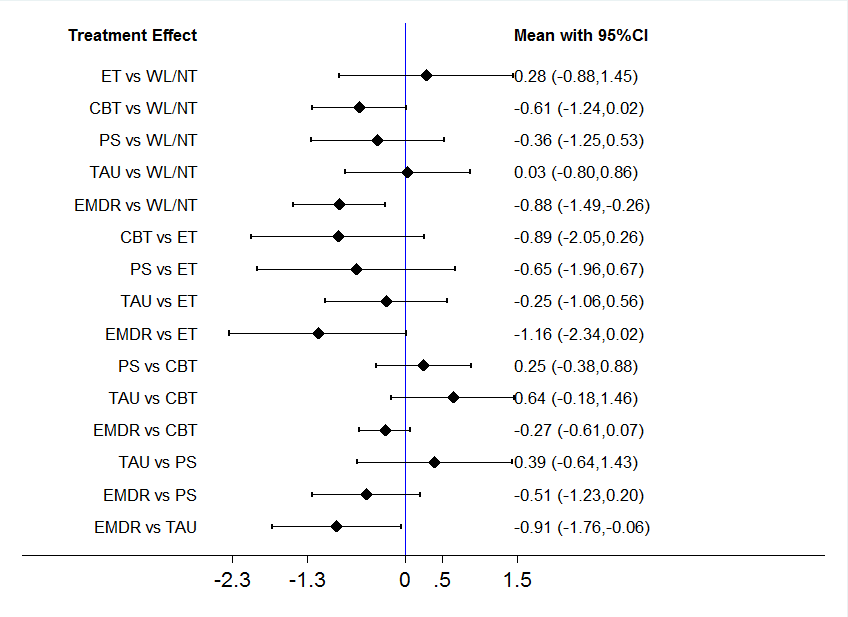


**Evaluation of incoherence**

**Overall incoherence**

Design-by-treatment test: P= 0.175

**Loop-specific heterogeneity**

| Loop | IF | seIF | z_value | p_value | CI_95 | Loop_Heterog_tau2 |
| --- | --- | --- | --- | --- | --- | --- |
| A-C-F | 0.816 | 0.594 | 1.373 | 0.170 | (0.00,1.98) | 0.018 |
| A-C-E | . | . | . | . |  | 0.000 |

**Consistency between direct and indirect estimates**

| Side | Direct Coef. | Std. Err. | Indirect Coef. | Std. Err. | Difference Coef. | Std. Err. | P>\|z\| | tau |
| --- | --- | --- | --- | --- | --- | --- | --- | --- |
| A C * | -1.014 | 0.426 | -0.198 | 0.423 | -0.816 | 0.600 | 0.174 | 0.145 |
| A E * | -0.182 | 0.434 | 1.448 | 1.115 | -1.630 | 1.200 | 0.174 | 0.145 |
| A F | -0.532 | 0.389 | -1.347 | 0.457 | 0.815 | 0.600 | 0.175 | 0.145 |
| B E * | -0.252 | 0.414 | 0.072 | 63.254 | -0.324 | 63.255 | 0.996 | 0.202 |
| C D * | 0.247 | 0.322 | 1.219 | 63.276 | -0.972 | 63.277 | 0.988 | 0.202 |
| C E * | 0.833 | 0.420 | -0.799 | 1.130 | 1.631 | 1.200 | 0.174 | 0.145 |
| C F | -0.334 | 0.166 | 0.482 | 0.577 | -0.816 | 0.600 | 0.174 | 0.145 |

**SUCRA and cumulative probability plots**


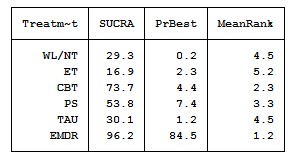


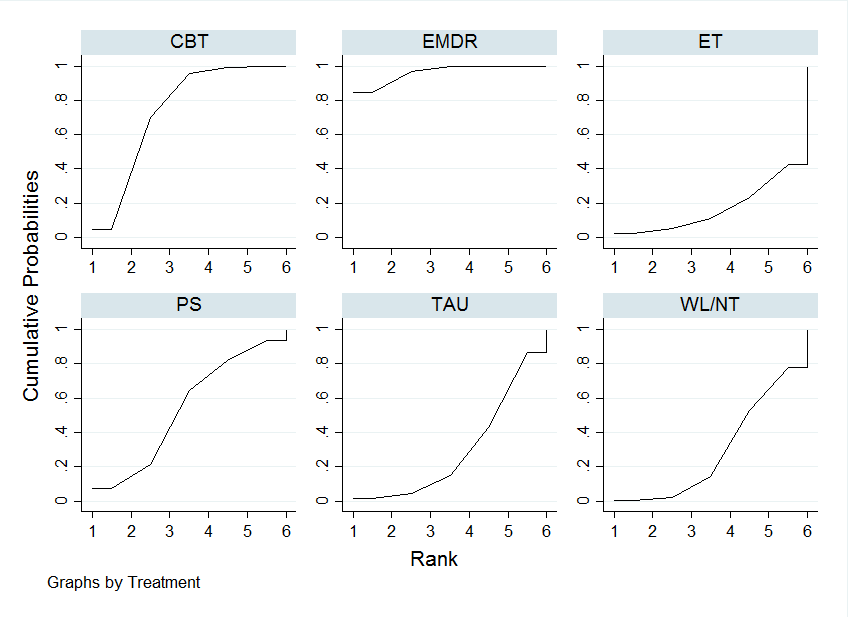


**Funnel Plot**


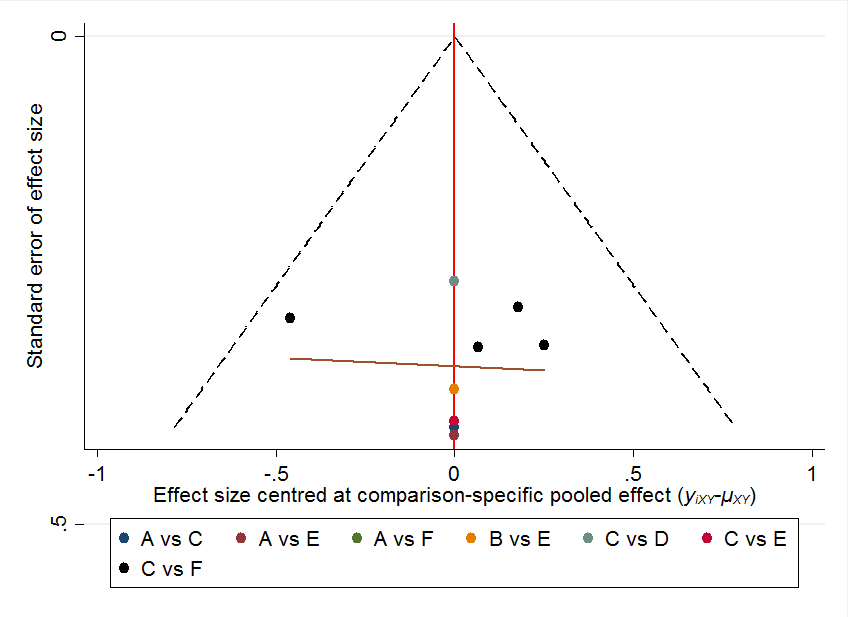


**Depression-post：**

Group n = 9 incoherence

Individual n=4 incoherence

**Depression- follow：**

Group n = 3 incoherence

Individual n=4 incoherence

# Subgroup analysis : Profession of Treatment Provider

**PTSD-post** B=3.739，95%CI[-1.505, 8.89]

Studies contributing to the analysis：

Psychologist/psychiatrist n = 16

| WL/NT | A |
| --- | --- |
| ET | B |
| CBT | C |
| EMDR | D |

Teacher/counselor n = 14

| WL/NT | A |
| --- | --- |
| ET | B |
| CBT | C |
| PS | D |
| TAU | E |
| BRI | F |
| MBT | G |
| PT | H |
| TGCT | I |

**Network map**

**-**Psychologist/psychiatrist


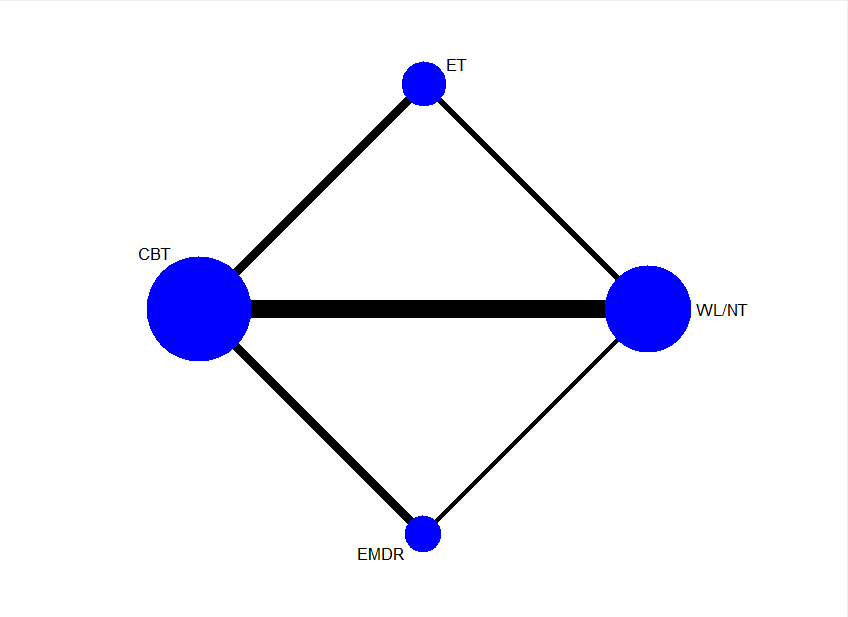


-Teacher/counselor


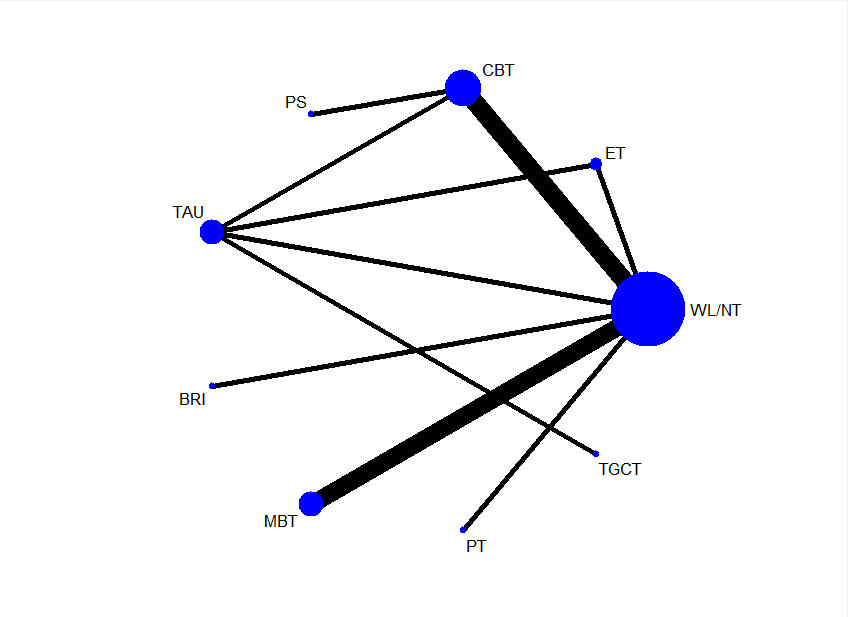


**Net league table**

**-**Psychologist/psychiatrist

| ET | 0.04 (-0.52,0.59) | 0.12 (-0.28,0.53) | 0.63 (0.20,1.06) |
| --- | --- | --- | --- |
| -0.04 (-0.59,0.52) | EMDR | 0.09 (-0.31,0.49) | 0.59 (0.16,1.03) |
| -0.12 (-0.53,0.28) | -0.09 (-0.49,0.31) | CBT | 0.51 (0.22,0.79) |
| -0.63 (-1.06,-0.20) | -0.59 (-1.03,-0.16) | -0.51 (-0.79,-0.22) | WL/NT |

-Teacher/counselor

| CBT | -0.02 (-1.36,1.32) | 0.21 (-1.52,1.94) | 0.32 (-0.92,1.55) | 0.43 (-0.72,1.58) | 0.58 (-0.35,1.51) | 0.68 (-0.76,2.11) | 0.71 (-0.72,2.14) | 0.89 (0.22,1.56) |
| --- | --- | --- | --- | --- | --- | --- | --- | --- |
| 0.02 (-1.32,1.36) | PS | 0.23 (-1.95,2.42) | 0.34 (-1.48,2.16) | 0.45 (-1.31,2.22) | 0.60 (-1.03,2.23) | 0.70 (-1.26,2.66) | 0.73 (-1.23,2.69) | 0.91 (-0.59,2.41) |
| -0.21 (-1.94,1.52) | -0.23 (-2.42,1.95) | TGCT | 0.10 (-1.62,1.83) | 0.22 (-1.08,1.51) | 0.36 (-1.45,2.18) | 0.46 (-1.65,2.58) | 0.50 (-1.62,2.61) | 0.68 (-1.02,2.37) |
| -0.32 (-1.55,0.92) | -0.34 (-2.16,1.48) | -0.10 (-1.83,1.62) | ET | 0.11 (-1.03,1.25) | 0.26 (-1.02,1.54) | 0.36 (-1.33,2.04) | 0.39 (-1.29,2.07) | 0.57 (-0.54,1.68) |
| -0.43 (-1.58,0.72) | -0.45 (-2.22,1.31) | -0.22 (-1.51,1.08) | -0.11 (-1.25,1.03) | TAU | 0.15 (-1.13,1.42) | 0.25 (-1.43,1.92) | 0.28 (-1.39,1.95) | 0.46 (-0.64,1.56) |
| -0.58 (-1.51,0.35) | -0.60 (-2.23,1.03) | -0.36 (-2.18,1.45) | -0.26 (-1.54,1.02) | -0.15 (-1.42,1.13) | MBT | 0.10 (-1.32,1.52) | 0.13 (-1.28,1.55) | 0.31 (-0.33,0.96) |
| -0.68 (-2.11,0.76) | -0.70 (-2.66,1.26) | -0.46 (-2.58,1.65) | -0.36 (-2.04,1.33) | -0.25 (-1.92,1.43) | -0.10 (-1.52,1.32) | BRI | 0.03 (-1.76,1.82) | 0.21 (-1.05,1.48) |
| -0.71 (-2.14,0.72) | -0.73 (-2.69,1.23) | -0.50 (-2.61,1.62) | -0.39 (-2.07,1.29) | -0.28 (-1.95,1.39) | -0.13 (-1.55,1.28) | -0.03 (-1.82,1.76) | PT | 0.18 (-1.08,1.44) |
| -0.89 (-1.56,-0.22) | -0.91 (-2.41,0.59) | -0.68 (-2.37,1.02) | -0.57 (-1.68,0.54) | -0.46 (-1.56,0.64) | -0.31 (-0.96,0.33) | -0.21 (-1.48,1.05) | -0.18 (-1.44,1.08) | WL |

**Interval Plot**

**-**Psychologist/psychiatrist


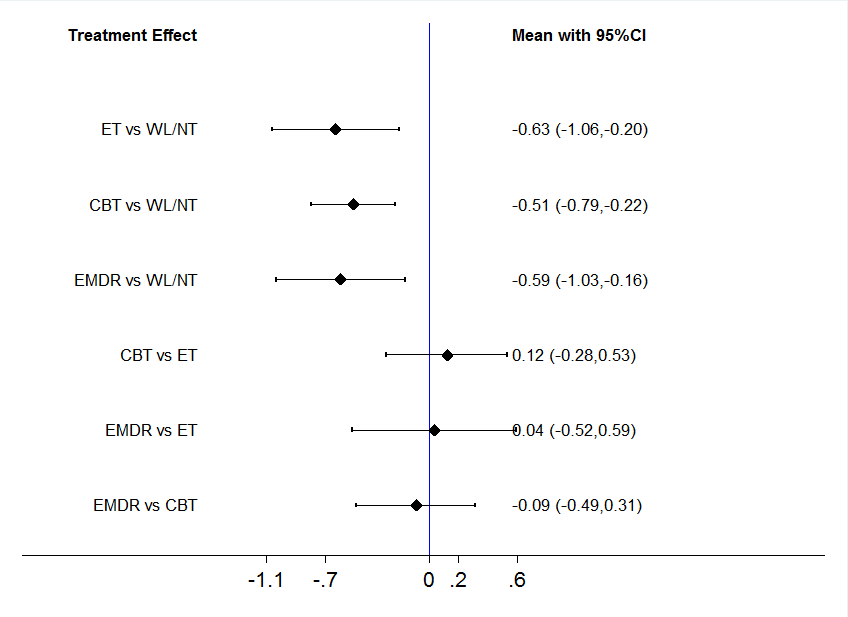


-Teacher/counselor


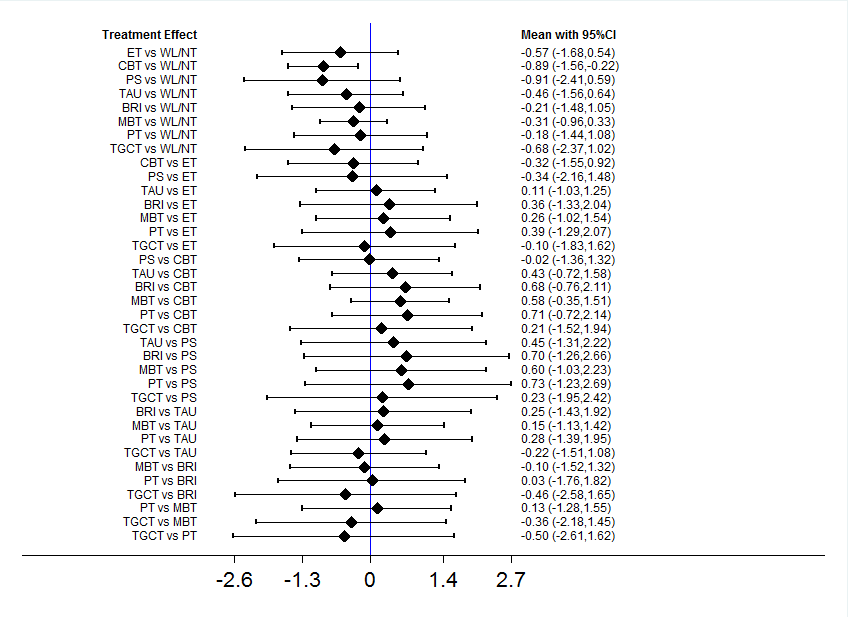


**Evaluation of incoherence**

**-**Psychologist/psychiatrist

**Overall incoherence**

Design-by-treatment test: P= 0.867

**Loop-specific heterogeneity**

| Loop | IF | seIF | z_value | p_value | CI_95 | Loop_Heterog_tau2 |
| --- | --- | --- | --- | --- | --- | --- |
| A-C-D | 0.156 | 0.548 | 0.284 | 0.776 | (0.00,1.23) | 0.128 |
| A-B-C | 0.113 | 0.573 | 0.197 | 0.844 | (0.00,1.24) | 0.199 |

**Consistency between direct and indirect estimates**

| Side | Direct Coef. | Std. Err. | Indirect Coef. | Std. Err. | Difference Coef. | Std. Err. | P>\|z\| | tau |
| --- | --- | --- | --- | --- | --- | --- | --- | --- |
| A B | A B | -0.638 | 0.275 | -0.630 | 0.424 | -0.008 | 0.505 | 0.987 |
| A C | A C | -0.522 | 0.165 | -0.469 | 0.410 | -0.053 | 0.443 | 0.904 |
| A D | A D | -0.486 | 0.366 | -0.673 | 0.300 | 0.187 | 0.474 | 0.693 |
| A E * | B C * | 0.149 | 0.236 | -0.068 | 0.606 | 0.216 | 0.648 | 0.738 |
| A G | C D | -0.138 | 0.253 | 0.049 | 0.401 | -0.187 | 0.474 | 0.693 |

-Teacher/counselor

**Overall incoherence**

Design-by-treatment test: P= 0.833

**Loop-specific heterogeneity**

| Loop | IF | seIF | z_value | p_value | CI_95 | Loop_Heterog_tau2 |
| --- | --- | --- | --- | --- | --- | --- |
| A-B-E | 0.483 | 0.605 | 0.799 | 0.424 | (0.00,1.67) | 0.000 |
| A-C-E | 0.325 | 0.574 | 0.566 | 0.571 | (0.00,1.45) | 0.000 |

**Consistency between direct and indirect estimates**

| Side | Direct Coef. | Std. Err. | Indirect Coef. | Std. Err. | Difference Coef. | Std. Err. | P>\|z\| | tau |
| --- | --- | --- | --- | --- | --- | --- | --- | --- |
| A B | -0.660 | 0.737 | -0.391 | 1.055 | -0.269 | 1.287 | 0.835 | 0.688 |
| A C * | -0.881 | 0.370 | -1.405 | 2.534 | 0.524 | 2.563 | 0.838 | 0.687 |
| A E | -0.163 | 0.786 | -0.826 | 0.872 | 0.663 | 1.174 | 0.573 | 0.672 |
| A F | . | . | . | . | . | . | . | . |
| A G | . | . | . | . | . | . | . | . |
| A H | . | . | . | . | . | . | . | . |
| B E | 0.014 | 0.776 | 0.282 | 1.026 | -0.268 | 1.287 | 0.835 | 0.688 |
| C D * | -0.021 | 0.683 | 1.782 | 63.265 | -1.803 | 63.269 | 0.977 | 0.635 |
| C E | 0.295 | 0.787 | 0.659 | 1.015 | -0.364 | 1.280 | 0.776 | 0.688 |
| E I * | -0.216 | 0.660 | 0.931 | 63.265 | -1.148 | 63.268 | 0.986 | 0.635 |

**SUCRA and cumulative probability plots**

**-**Psychologist/psychiatrist


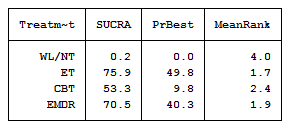


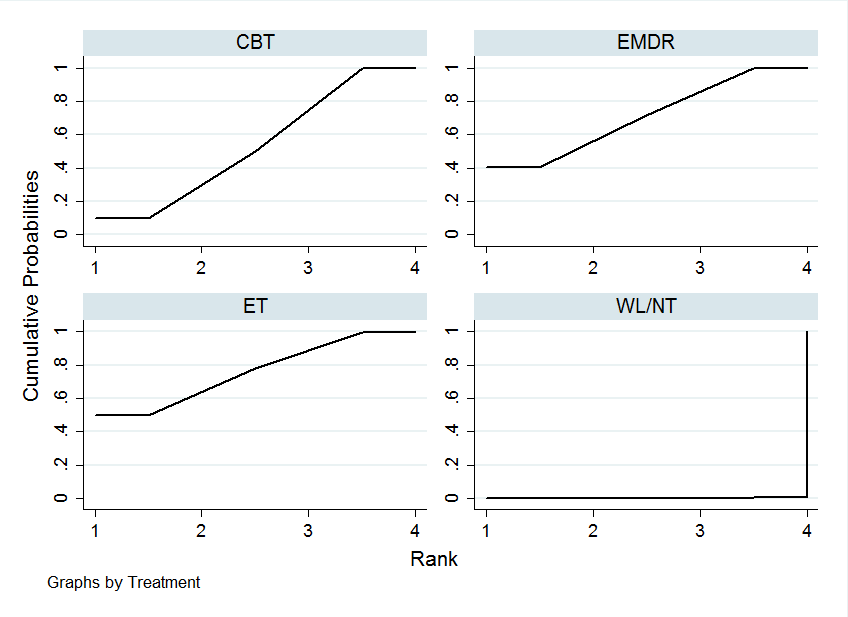


-Teacher/counselor


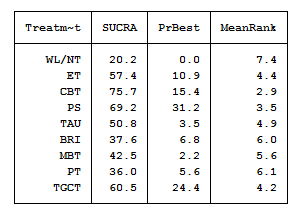


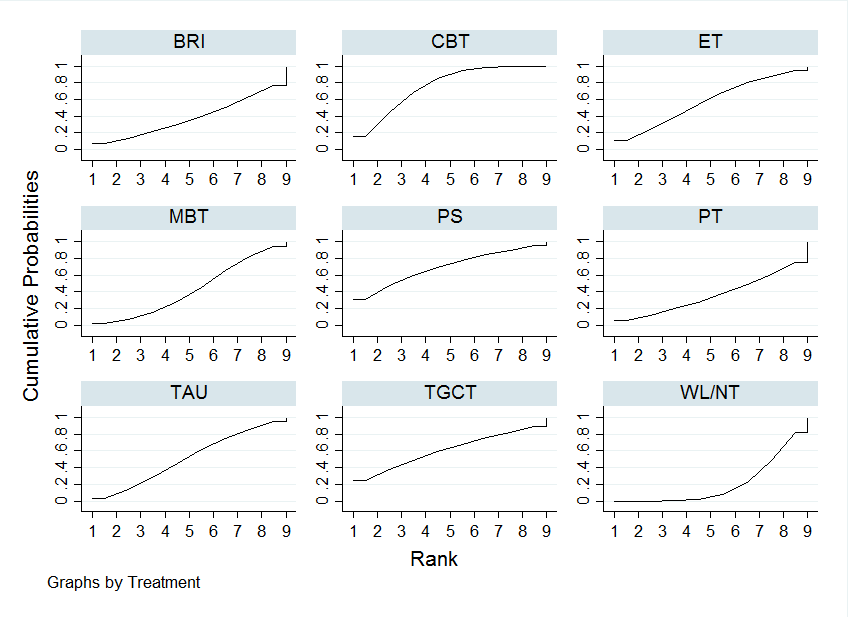


**Funnel Plot**

**-**Psychologist/psychiatrist


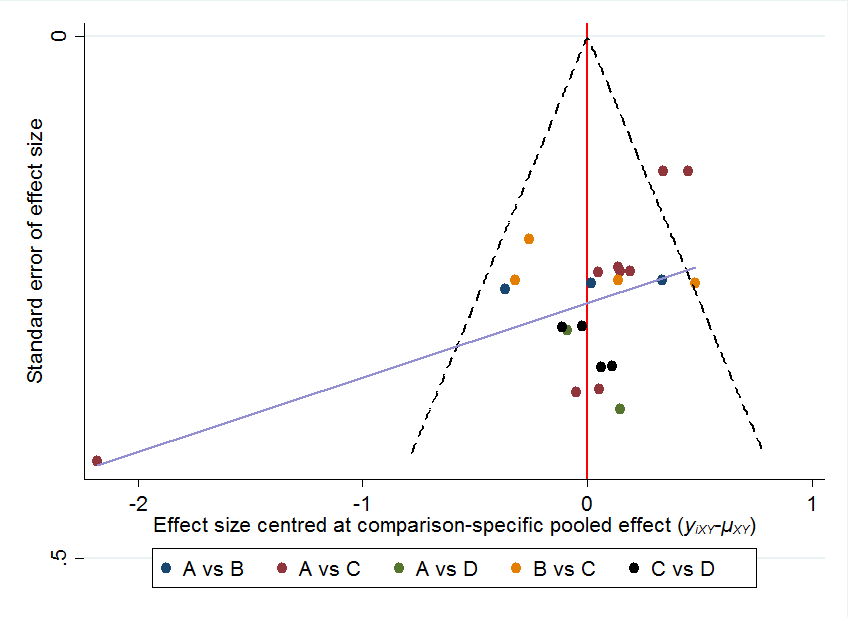


-Teacher/counselor


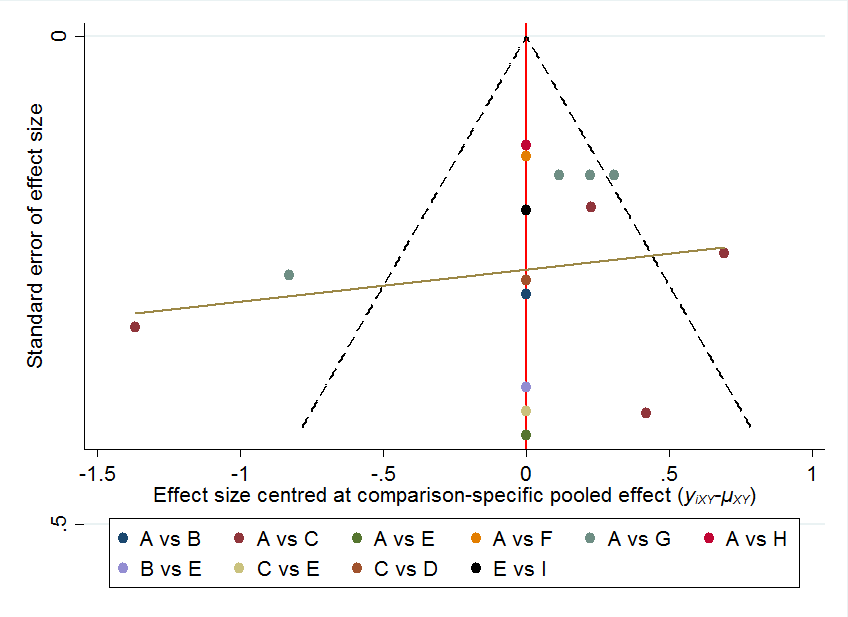


**PTSD-follow：**

Teacher/counselor n=5 incoherence

Psychologist n = 10

| WL/NT | A |
| --- | --- |
| ET | B |
| CBT | C |
| EMDR | D |

**Network map**


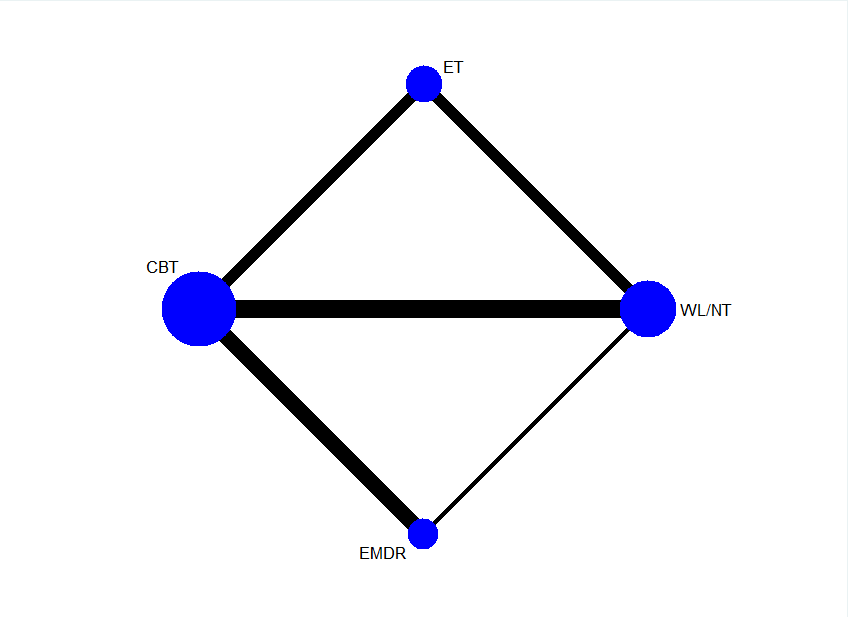


**Net league table**

| EMDR | 0.09 (-0.31,0.49) | 0.29 (0.02,0.56) | 0.80 (0.45,1.14) |
| --- | --- | --- | --- |
| -0.09 (-0.49,0.31) | ET | 0.20 (-0.11,0.51) | 0.71 (0.38,1.03) |
| -0.29 (-0.56,-0.02) | -0.20 (-0.51,0.11) | CBT | 0.51 (0.26,0.76) |
| -0.80 (-1.14,-0.45) | -0.71 (-1.03,-0.38) | -0.51 (-0.76,-0.26) | WL/NT |

**Interval Plot**


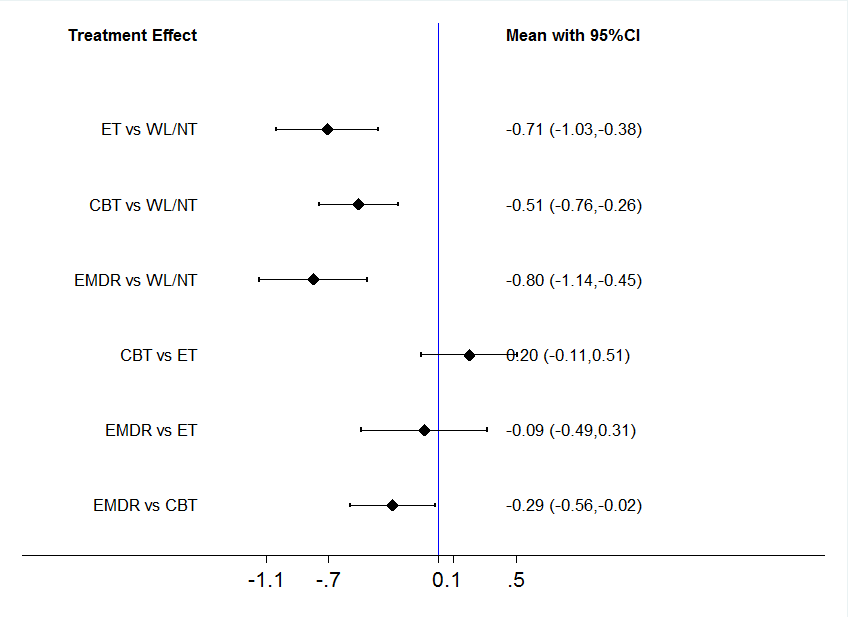


**Evaluation of incoherence**

**Overall incoherence**

Design-by-treatment test: P= 0.712

**Loop-specific heterogeneity**

| Loop | IF | seIF | z_value | p_value | CI_95 | Loop_Heterog_tau2 |
| --- | --- | --- | --- | --- | --- | --- |
| A-B-E | 1.428 | 0.630 | 2.267 | 0.023 | (0.19,2.66) | 0.000 |
| A-B-D | . | . | . | . |  | 0.000 |

**Consistency between direct and indirect estimates**

| Side | Direct Coef. | Std. Err. | Indirect Coef. | Std. Err. | Difference Coef. | Std. Err. | P>\|z\| | tau |
| --- | --- | --- | --- | --- | --- | --- | --- | --- |
| A B * | -0.737 | 0.179 | -0.492 | 0.501 | -0.245 | 0.532 | 0.645 | 0.054 |
| A C * | -0.545 | 0.135 | -0.197 | 0.391 | -0.348 | 0.414 | 0.400 | 0.000 |
| A D | -0.532 | 0.361 | -0.880 | 0.202 | 0.348 | 0.414 | 0.400 | 0.000 |
| B C * | 0.181 | 0.167 | 0.426 | 0.514 | -0.245 | 0.532 | 0.645 | 0.054 |
| C D | -0.335 | 0.149 | 0.013 | 0.386 | -0.348 | 0.414 | 0.400 | 0.000 |

**SUCRA and cumulative probability plots**


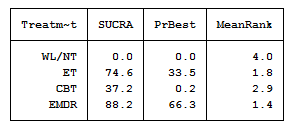


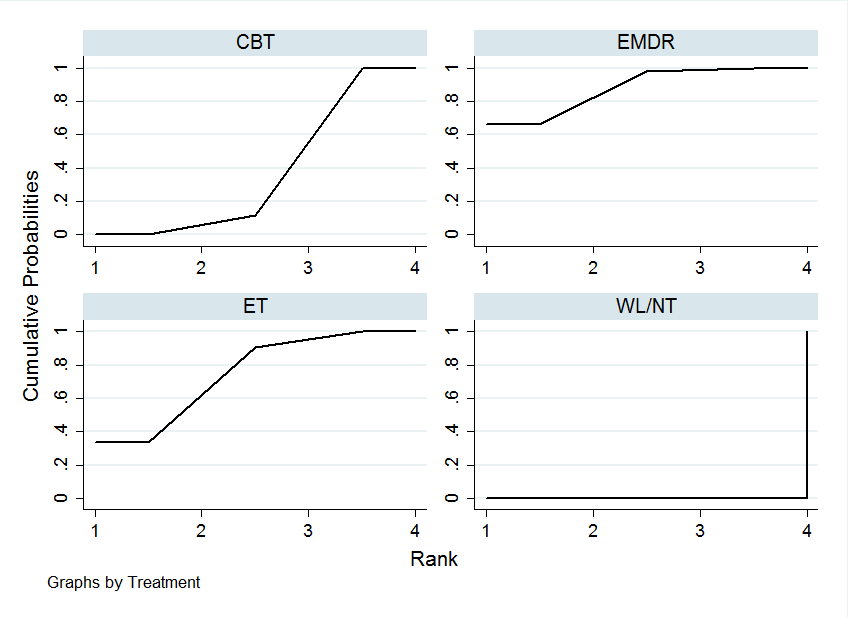


**Funnel Plot**


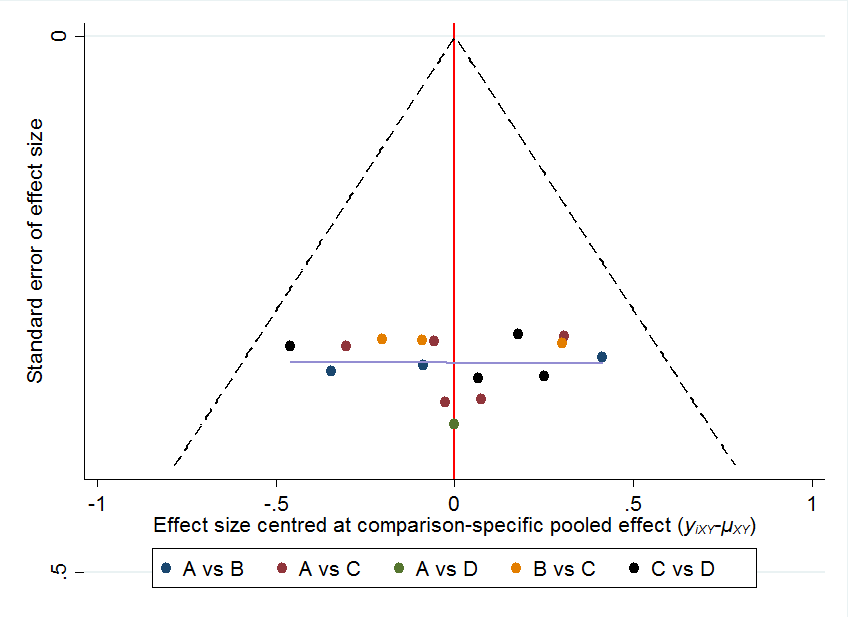


Depression-post：

Psychologist n = 6 incoherence

Teacher n=7

| WL/NT | A |
| --- | --- |
| CBT | B |
| PS | C |
| TAU | D |
| PT | E |
| TGCT | F |

**Network map**


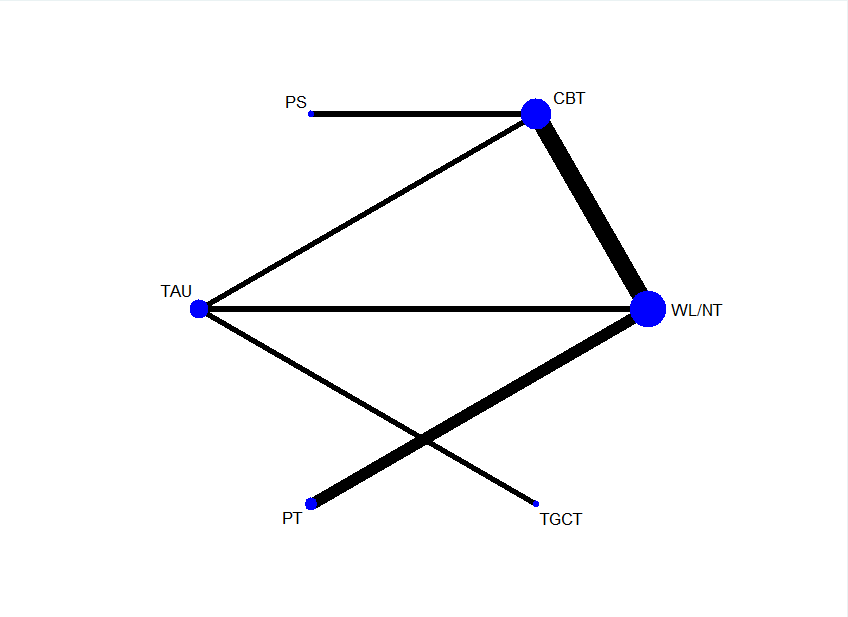


**Net league table**

| PT | 0.11 (-0.69,0.91) | 0.20 (-0.52,0.93) | 0.34 (0.02,0.67) | 0.37 (0.16,0.58) | 0.53 (-0.06,1.12) |
| --- | --- | --- | --- | --- | --- |
| -0.11 (-0.91,0.69) | TGCT | 0.10 (-0.26,0.45) | 0.23 (-0.54,1.00) | 0.26 (-0.52,1.04) | 0.42 (-0.49,1.34) |
| -0.20 (-0.93,0.52) | -0.10 (-0.45,0.26) | TAU | 0.14 (-0.55,0.82) | 0.16 (-0.53,0.86) | 0.33 (-0.51,1.17) |
| -0.34 (-0.67,-0.02) | -0.23 (-1.00,0.54) | -0.14 (-0.82,0.55) | CBT | 0.03 (-0.22,0.28) | 0.19 (-0.30,0.68) |
| -0.37 (-0.58,-0.16) | -0.26 (-1.04,0.52) | -0.16 (-0.86,0.53) | -0.03 (-0.28,0.22) | WL/NT | 0.16 (-0.39,0.72) |
| -0.53 (-1.12,0.06) | -0.42 (-1.34,0.49) | -0.33 (-1.17,0.51) | -0.19 (-0.68,0.30) | -0.16 (-0.72,0.39) | PS |

**Interval Plot**


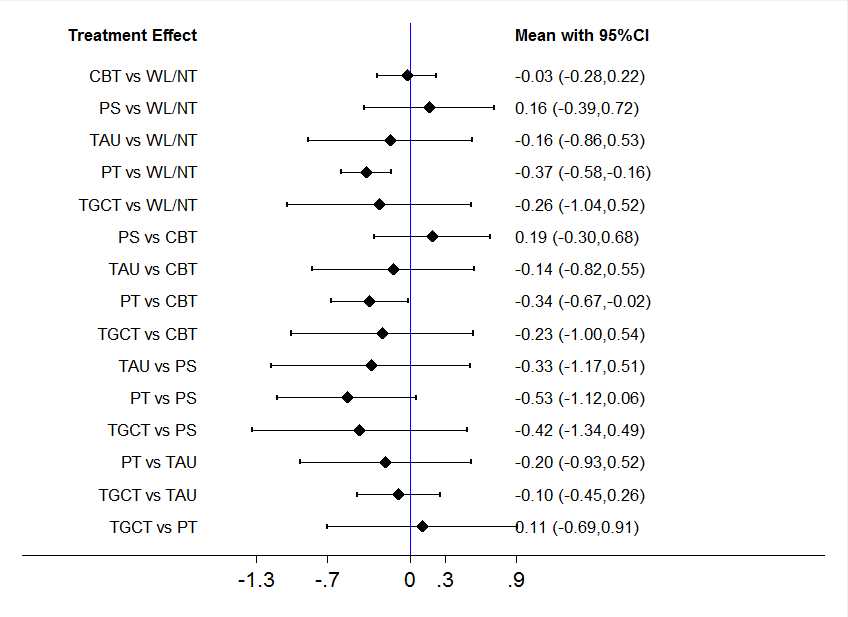


**Evaluation of incoherence**

**Overall incoherence**

Design-by-treatment test: P= 0.703

**Loop-specific heterogeneity**

| Loop | IF | seIF | z_value | p_value | CI_95 | Loop_Heterog_tau2 |
| --- | --- | --- | --- | --- | --- | --- |
| A-B-D | 0.155 | 0.576 | 0.269 | 0.788 | (0.00,1.28) | 0.000 |

**Consistency between direct and indirect estimates**

| Side | Direct Coef. | Std. Err. | Indirect Coef. | Std. Err. | Difference Coef. | Std. Err. | P>\|z\| | tau |
| --- | --- | --- | --- | --- | --- | --- | --- | --- |
| A B * | (0.026) | 0.127 | 0.004 | 28.315 | (0.030) | 28.315 | 0.999 | 0.000 |
| A D * | (0.086) | 0.408 | (0.395) | 0.701 | 0.309 | 0.810 | 0.703 | 0.000 |
| A E | . | . | . | . | . | . | . | . |
| B C * | 0.190 | 0.251 | 0.052 | 63.362 | 0.139 | 63.363 | 0.998 | 0.000 |
| B D * | (0.198) | 0.383 | 0.112 | 0.743 | (0.310) | 0.810 | 0.703 | 0.000 |
| D F * | (0.095) | 0.179 | 0.333 | 63.175 | (0.428) | 63.175 | 0.995 | 0.000 |

**SUCRA and cumulative probability plots**


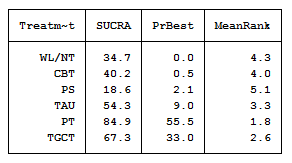


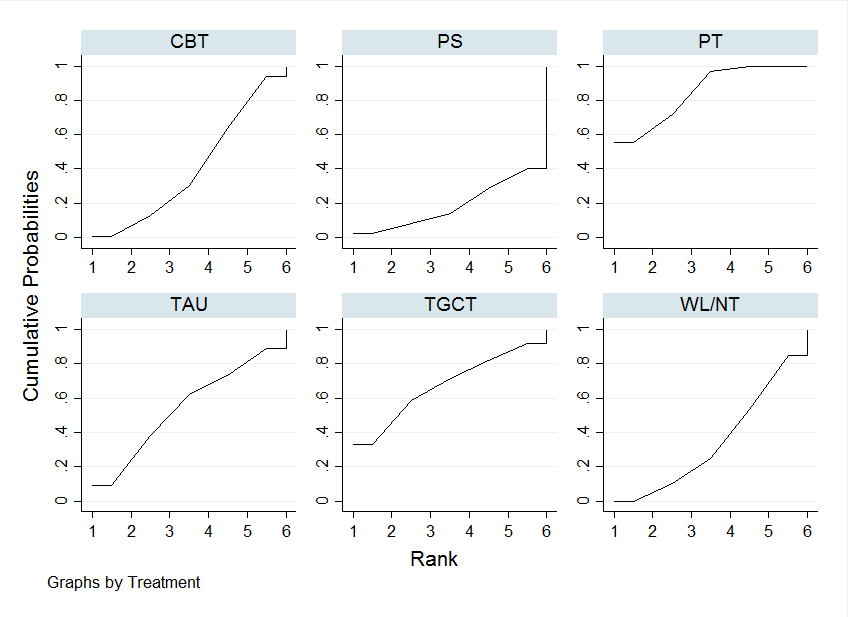


**Funnel Plot**


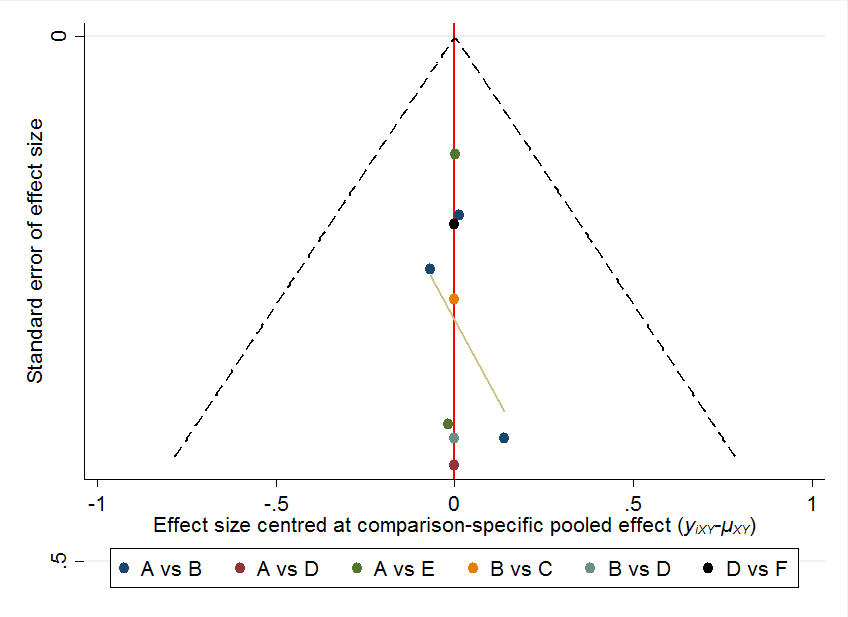


**Depression-follow：**

Psychologist n = 2 incoherence

Teacher/counselor n=5 incoherence

# Subgroup analysis : Country Income

**PTSD-post** B=3.418 95%CI(-5.401, 12.14)

Studies contributing to the analysis：

LMIC n = 22

| WL/NT | A |
| --- | --- |
| TAU | B |
| ET | C |
| EMDR | D |
| CBT | E |
| PS | F |
| MBT | G |
| TGCT | H |
| PT | I |

HIC n = 8

| WL/NT | A |
| --- | --- |
| ET | B |
| EMDR | C |
| CBT | D |
| BRI | E |

**Network map**

LMIC


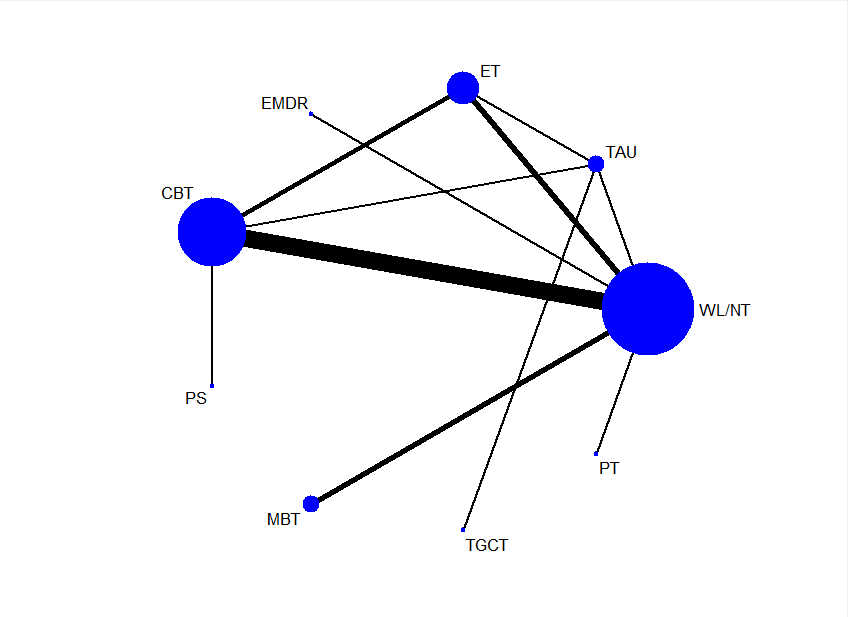


HIC


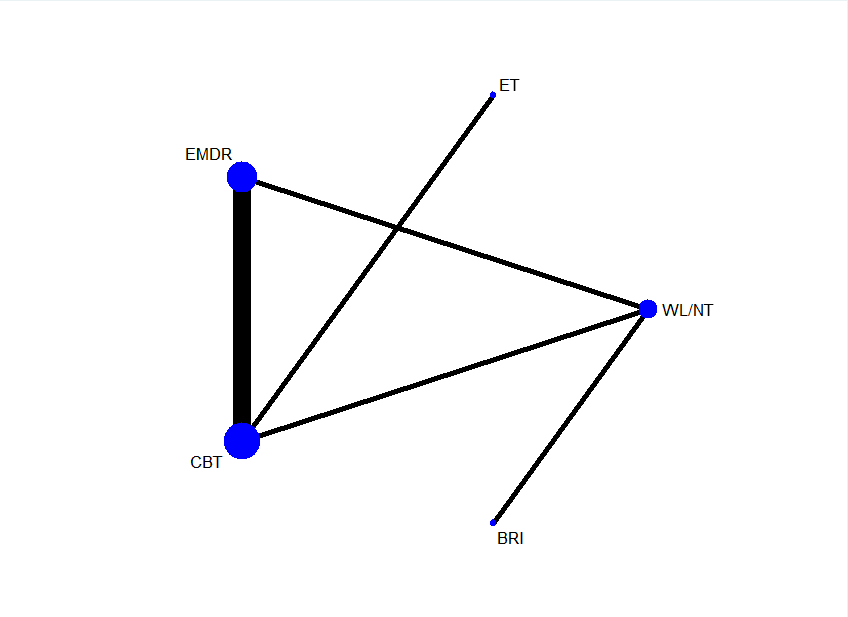


**Net league table**

LMIC

| ET | 0.03 (-0.55,0.61) | 0.01 (-1.38,1.39) | 0.04 (-1.50,1.57) | 0.13 (-1.27,1.53) | 0.25 (-0.70,1.20) | 0.41 (-0.41,1.23) | 0.54 (-0.76,1.85) | 0.72 (0.17,1.28) |
| --- | --- | --- | --- | --- | --- | --- | --- | --- |
| -0.03 (-0.61,0.55) | CBT | -0.02 (-1.28,1.24) | 0.01 (-1.53,1.55) | 0.10 (-1.23,1.44) | 0.22 (-0.73,1.18) | 0.38 (-0.32,1.09) | 0.52 (-0.72,1.75) | 0.70 (0.33,1.06) |
| -0.01 (-1.39,1.38) | 0.02 (-1.24,1.28) | PS | 0.03 (-1.96,2.02) | 0.12 (-1.71,1.96) | 0.24 (-1.34,1.82) | 0.40 (-1.04,1.85) | 0.54 (-1.23,2.30) | 0.72 (-0.59,2.03) |
| -0.04 (-1.57,1.50) | -0.01 (-1.55,1.53) | -0.03 (-2.02,1.96) | TGCT | 0.09 (-1.91,2.10) | 0.22 (-0.99,1.43) | 0.38 (-1.28,2.03) | 0.51 (-1.43,2.45) | 0.69 (-0.85,2.23) |
| -0.13 (-1.53,1.27) | -0.10 (-1.44,1.23) | -0.12 (-1.96,1.71) | -0.09 (-2.10,1.91) | EMDR | 0.12 (-1.48,1.72) | 0.28 (-1.14,1.70) | 0.41 (-1.33,2.16) | 0.59 (-0.69,1.88) |
| -0.25 (-1.20,0.70) | -0.22 (-1.18,0.73) | -0.24 (-1.82,1.34) | -0.22 (-1.43,0.99) | -0.12 (-1.72,1.48) | TAU | 0.16 (-0.96,1.29) | 0.29 (-1.22,1.81) | 0.47 (-0.48,1.42) |
| -0.41 (-1.23,0.41) | -0.38 (-1.09,0.32) | -0.40 (-1.85,1.04) | -0.38 (-2.03,1.28) | -0.28 (-1.70,1.14) | -0.16 (-1.29,0.96) | MBT | 0.13 (-1.19,1.46) | 0.31 (-0.29,0.92) |
| -0.54 (-1.85,0.76) | -0.52 (-1.75,0.72) | -0.54 (-2.30,1.23) | -0.51 (-2.45,1.43) | -0.41 (-2.16,1.33) | -0.29 (-1.81,1.22) | -0.13 (-1.46,1.19) | PT | 0.18 (-1.00,1.36) |
| -0.72 (-1.28,-0.17) | -0.70 (-1.06,-0.33) | -0.72 (-2.03,0.59) | -0.69 (-2.23,0.85) | -0.59 (-1.88,0.69) | -0.47 (-1.42,0.48) | -0.31 (-0.92,0.29) | -0.18 (-1.36,1.00) | WL/NT |

HIC

| EMDR | 0.13 (-0.36,0.61) | 0.15 (-0.13,0.42) | 0.26 (-0.21,0.73) | 0.34 (-0.08,0.76) |
| --- | --- | --- | --- | --- |
| -0.13 (-0.61,0.36) | BRI | 0.02 (-0.43,0.47) | 0.13 (-0.46,0.72) | 0.21 (-0.03,0.46) |
| -0.15 (-0.42,0.13) | -0.02 (-0.47,0.43) | CBT | 0.11 (-0.27,0.49) | 0.20 (-0.18,0.57) |
| -0.26 (-0.73,0.21) | -0.13 (-0.72,0.46) | -0.11 (-0.49,0.27) | ET | 0.08 (-0.45,0.62) |
| -0.34 (-0.76,0.08) | -0.21 (-0.46,0.03) | -0.20 (-0.57,0.18) | -0.08 (-0.62,0.45) | WL/NT |

**Interval Plot**

LMIC


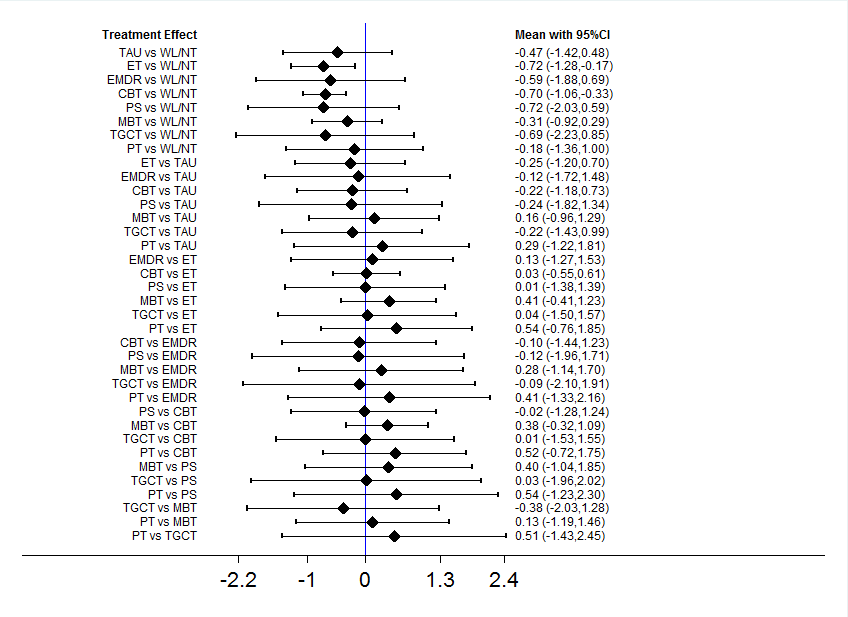


HIC


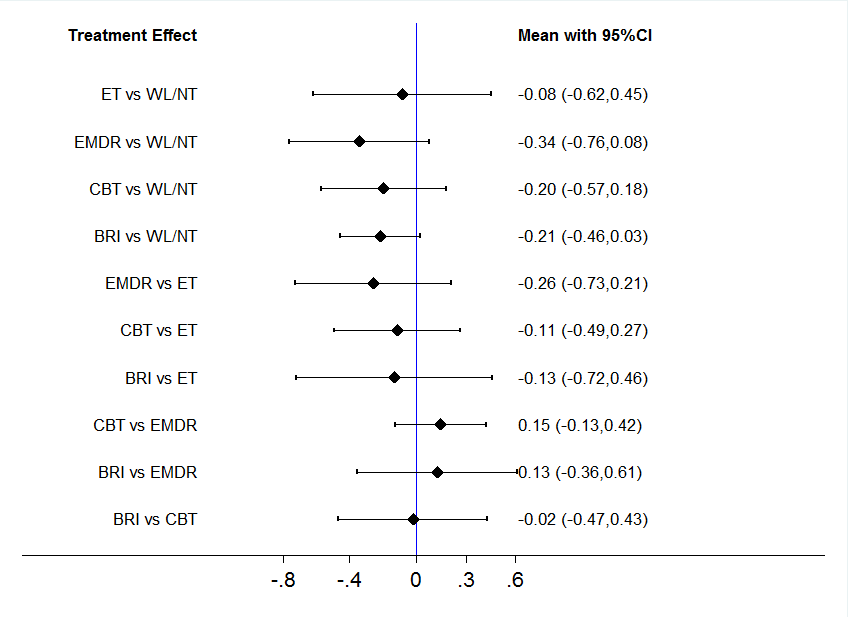


**Evaluation of incoherence**

LMIC

**Overall incoherence**

Design-by-treatment test: P= 0.872

**Loop-specific heterogeneity**

| Loop | IF | seIF | z_value | p_value | CI_95 | Loop_Heterog_tau2 |
| --- | --- | --- | --- | --- | --- | --- |
| B-C-E | 0.53 | 0.735 | 0.722 | 0.47 | (0.00,1.97) | 0.105 |
| A-B-C | 0.47 | 0.608 | 0.765 | 0.444 | (0.00,1.66) | 0.026 |
| A-C-E | 0.4 | 0.602 | 0.656 | 0.512 | (0.00,1.57) | 0.303 |
| A-B-E | 0.25 | 1.309 | 0.188 | 0.851 | (0.00,2.81) | 0.313 |

**Consistency between direct and indirect estimates**

| Side | Direct Coef. | Std. Err. | Indirect Coef. | Std. Err. | Difference Coef. | Std. Err. | P>\|z\| | tau |
| --- | --- | --- | --- | --- | --- | --- | --- | --- |
| A B | -0.163 | 0.731 | -0.732 | 0.669 | 0.569 | 0.991 | 0.566 | 0.606 |
| A C | -0.645 | 0.328 | -1.019 | 0.628 | 0.374 | 0.707 | 0.597 | 0.609 |
| A D | . | . | . | . | . | . | . | . |
| A E * | -0.701 | 0.194 | -0.537 | 1.474 | -0.163 | 1.486 | 0.913 | 0.615 |
| A G | . | . | . | . | . | . | . | . |
| A I | . | . | . | . | . | . | . | . |
| B C | -0.014 | 0.705 | -0.482 | 0.694 | 0.468 | 0.990 | 0.636 | 0.607 |
| B E | -0.294 | 0.722 | -0.162 | 0.683 | -0.132 | 0.989 | 0.894 | 0.611 |
| B H * | -0.216 | 0.618 | 0.954 | 63.273 | -1.170 | 63.276 | 0.985 | 0.591 |
| C E | 0.244 | 0.371 | -0.355 | 0.494 | 0.600 | 0.617 | 0.331 | 0.598 |
| E F * | -0.021 | 0.642 | 1.390 | 63.254 | -1.411 | 63.257 | 0.982 | 0.591 |

HIC

**Overall incoherence**

Design-by-treatment test: P= 0.959

**Loop-specific heterogeneity**

| Loop | IF | seIF | z_value | p_value | CI_95 | Loop_Heterog_tau2 |
| --- | --- | --- | --- | --- | --- | --- |
| A-C-D | 0.02 | 0.446 | 0.052 | 0.959 | (0.00,0.90) | 0 |

**Consistency between direct and indirect estimates**

| Side | Direct Coef. | Std. Err. | Indirect Coef. | Std. Err. | Difference Coef. | Std. Err. | P>\|z\| | tau |
| --- | --- | --- | --- | --- | --- | --- | --- | --- |
| A C | -0.357 | 0.357 | -0.334 | 0.267 | -0.023 | 0.446 | 0.958 | 0.000 |
| A D | -0.189 | 0.222 | -0.212 | 0.387 | 0.023 | 0.446 | 0.959 | 0.000 |
| A E | . | . | . | . | . | . | . | . |
| B D * | -0.112 | 0.194 | -0.416 | 63.217 | 0.304 | 63.217 | 0.996 | 0.000 |
| C D | 0.144 | 0.148 | 0.167 | 0.421 | -0.023 | 0.446 | 0.959 | 0.000 |

**SUCRA and cumulative probability plots**

LMIC

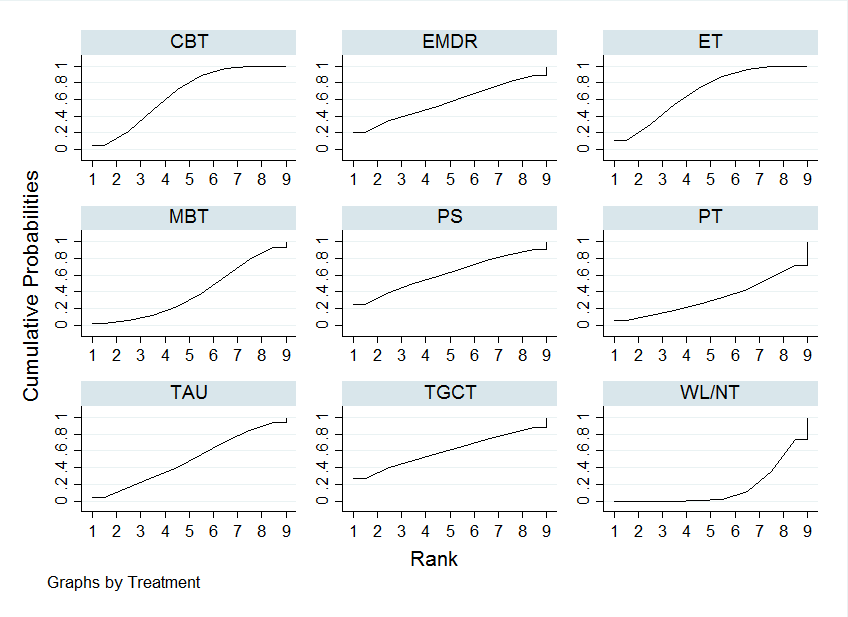


HIC

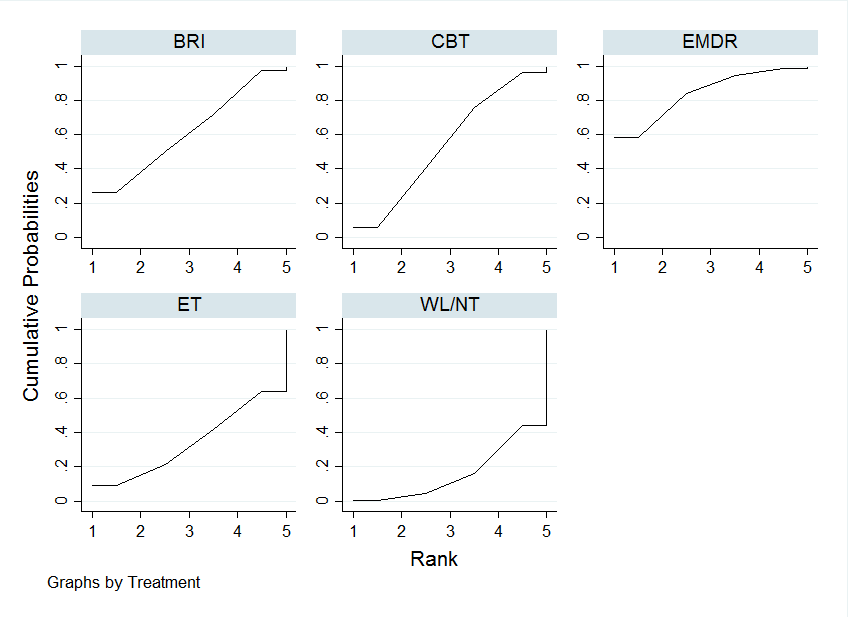


**Funnel Plot**

LMIC


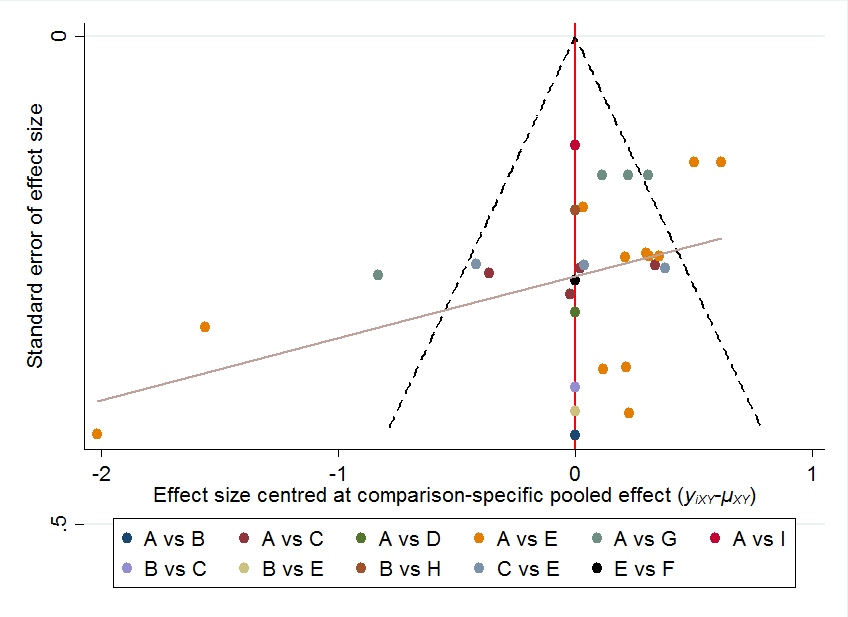


HIC


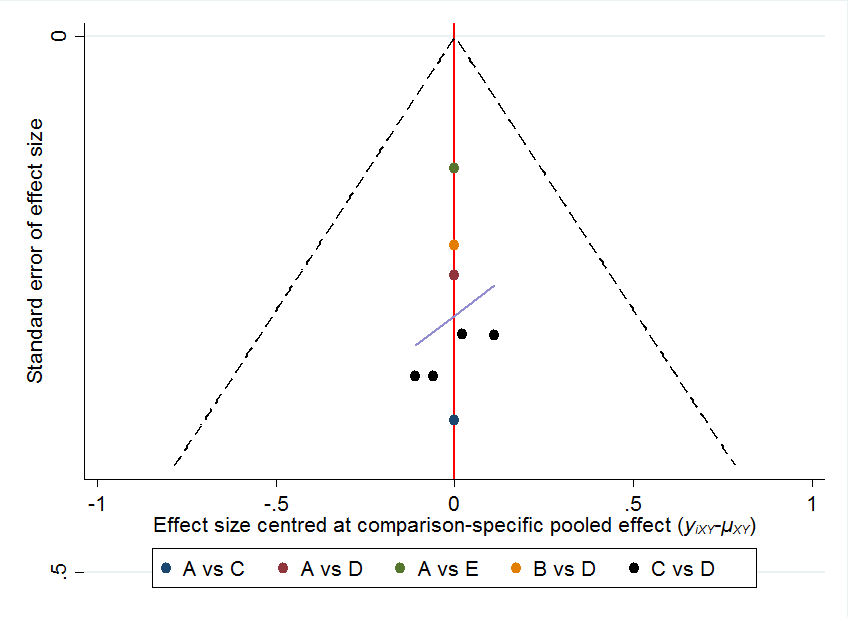


**PTSD-follow**

HIC n=5 incoherence

LMIC n=10

| WL/NT | A |
| --- | --- |
| ET | B |
| CBT | C |
| PS | D |
| TAU | E |
| PSS | F |
| TGCT | G |

**Network map**


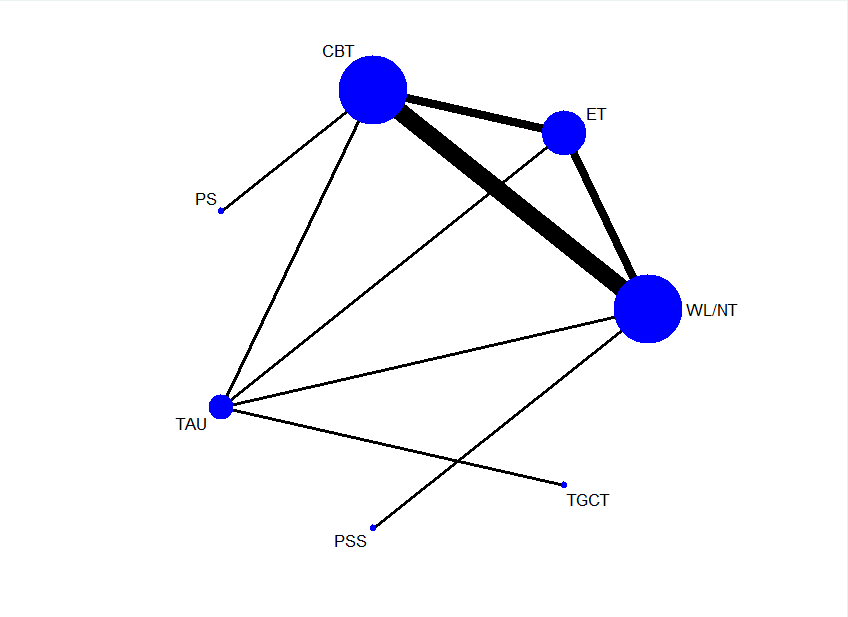


**Net league table**

| ET | 0.07 (-0.26,0.41) | 0.14 (-0.64,0.92) | 0.25 (-0.29,0.80) | 0.32 (-0.33,0.97) | 0.62 (0.17,1.07) | 0.67 (0.33,1.01) |
| --- | --- | --- | --- | --- | --- | --- |
| -0.07 (-0.41,0.26) | CBT | 0.07 (-0.72,0.86) | 0.18 (-0.38,0.74) | 0.25 (-0.31,0.81) | 0.54 (0.14,0.94) | 0.60 (0.32,0.87) |
| -0.14 (-0.92,0.64) | -0.07 (-0.86,0.72) | TGCT | 0.11 (-0.45,0.67) | 0.18 (-0.79,1.15) | 0.47 (-0.37,1.32) | 0.53 (-0.27,1.32) |
| -0.25 (-0.80,0.29) | -0.18 (-0.74,0.38) | -0.11 (-0.67,0.45) | TAU | 0.07 (-0.72,0.86) | 0.36 (-0.27,1.00) | 0.42 (-0.15,0.98) |
| -0.32 (-0.97,0.33) | -0.25 (-0.81,0.31) | -0.18 (-1.15,0.79) | -0.07 (-0.86,0.72) | PQ | 0.30 (-0.39,0.99) | 0.35 (-0.28,0.97) |
| -0.62 (-1.07,-0.17) | -0.54 (-0.94,-0.14) | -0.47 (-1.32,0.37) | -0.36 (-1.00,0.27) | -0.30 (-0.99,0.39) | PSS | 0.05 (-0.24,0.34) |
| -0.67 (-1.01,-0.33) | -0.60 (-0.87,-0.32) | -0.53 (-1.32,0.27) | -0.42 (-0.98,0.15) | -0.35 (-0.97,0.28) | -0.05 (-0.34,0.24) | WL/NT |

**Interval Plot**


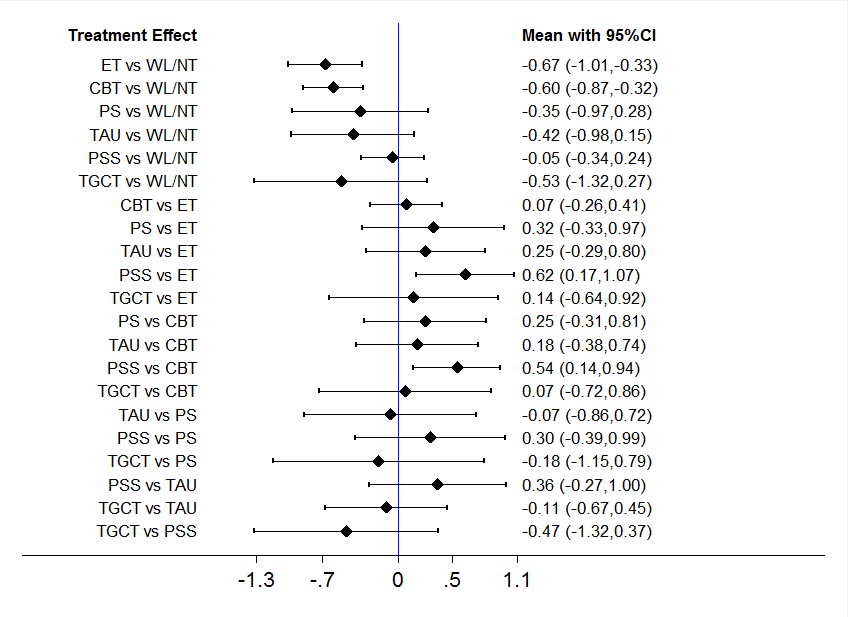


**Evaluation of incoherence**

**Overall incoherence**

Design-by-treatment test: P= 0.151

**Loop-specific heterogeneity**

| Loop | IF | seIF | z_value | p_value | CI_95 | Loop_Heterog_tau2 |
| --- | --- | --- | --- | --- | --- | --- |
| B-C-E | 1.267 | 0.559 | 2.265 | 0.024 | (0.17,2.36) | 0.000 |
| A-B-E | 0.813 | 0.678 | 1.200 | 0.230 | (0.00,2.14) | 0.056 |
| A-C-E | 0.467 | 0.584 | 0.799 | 0.424 | (0.00,1.61) | 0.000 |
| A-B-C | 0.096 | 0.319 | 0.302 | 0.763 | (0.00,0.72) | 0.001 |

**Consistency between direct and indirect estimates**

| Side | Direct Coef. | Std. Err. | Indirect Coef. | Std. Err. | Difference Coef. | Std. Err. | P>\|z\| | tau |
| --- | --- | --- | --- | --- | --- | --- | --- | --- |
| A B | -0.740 | 0.201 | -0.344 | 0.430 | -0.395 | 0.475 | 0.405 | 0.166 |
| A C * | -0.595 | 0.141 | 0.060 | 25.824 | -0.655 | 25.825 | 0.980 | 0.137 |
| A E | -0.171 | 0.436 | -0.613 | 0.392 | 0.442 | 0.588 | 0.452 | 0.152 |
| A F | . | . | . | . | . | . | . | . |
| B C | 0.173 | 0.173 | -0.470 | 0.403 | 0.642 | 0.434 | 0.139 | 0.098 |
| B E | -0.252 | 0.361 | 0.802 | 0.378 | -1.054 | 0.523 | 0.044 | 0.000 |
| C D * | 0.247 | 0.286 | 1.190 | 63.202 | -0.943 | 63.203 | 0.988 | 0.137 |
| C E | 0.829 | 0.394 | -0.370 | 0.357 | 1.200 | 0.529 | 0.023 | 0.000 |
| E G * | -0.109 | 0.285 | 0.844 | 63.254 | -0.954 | 63.255 | 0.988 | 0.137 |

**SUCRA and cumulative probability plots**


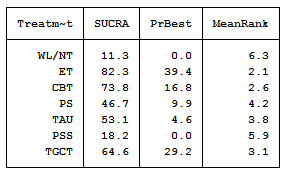


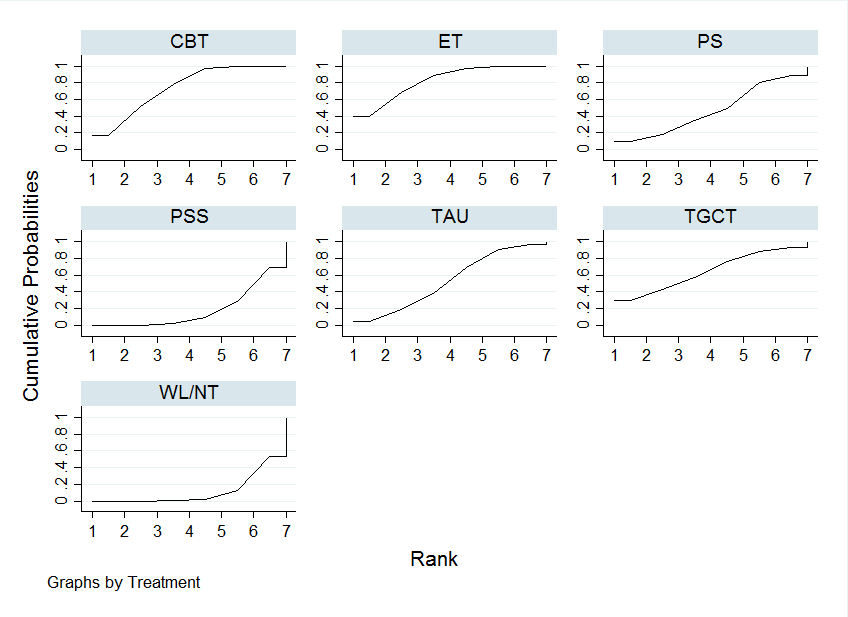


**Funnel Plot**


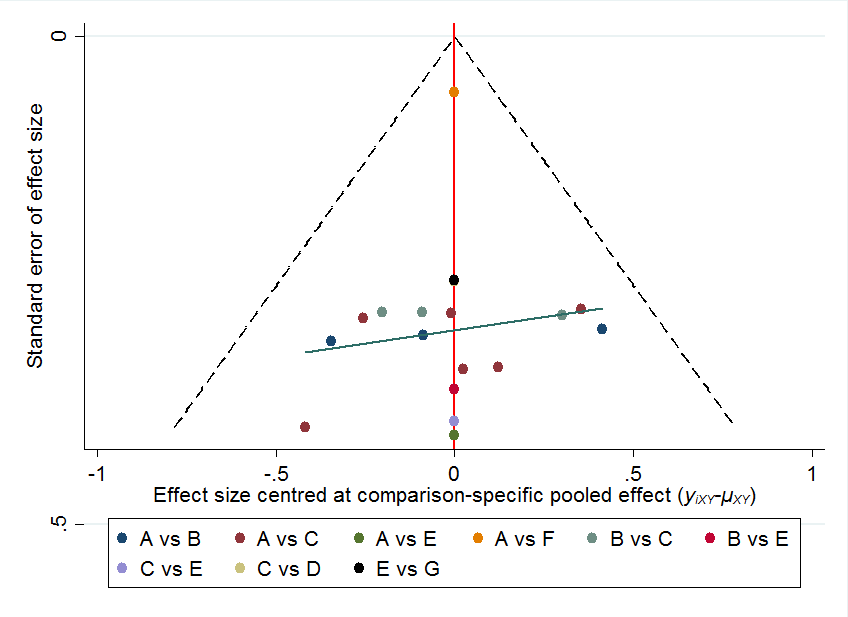


**Depression-post：**

HIC n=4 incoherence

LMIC n = 9 incoherence

**Depression- follow：**

HIC n=2 incoherence

LMIC n = 5 incoherence

# CINeMA-Final report

| Comparison | Number of studies | Within-study bias | Reporting bias | Indirectness | Imprecision | Heterogeneity | Incoherence | Confidence rating |
| --- | --- | --- | --- | --- | --- | --- | --- | --- |
| BRI:WL/NT | 1 | Some concerns | Some concerns | No concerns | Major concerns | No concerns | No concerns | Low |
| CBT:EMDR | 4 | No concerns | Some concerns | No concerns | Major concerns | No concerns | No concerns | Low |
| CBT:ET | 4 | Some concerns | Some concerns | No concerns | Major concerns | No concerns | No concerns | Low |
| CBT:PS | 1 | No concerns | Some concerns | No concerns | Major concerns | No concerns | No concerns | Low |
| CBT:TAU | 1 | Some concerns | Some concerns | No concerns | Major concerns | No concerns | No concerns | Low |
| CBT:WL/NT | 13 | Some concerns | Some concerns | No concerns | No concerns | Major concerns | No concerns | Low |
| EMDR:WL/NT | 2 | No concerns | Some concerns | No concerns | No concerns | Major concerns | No concerns | Low |
| ET:TAU | 1 | Some concerns | Some concerns | No concerns | Major concerns | No concerns | No concerns | Low |
| ET:WL/NT | 4 | Some concerns | Some concerns | No concerns | No concerns | Major concerns | No concerns | Low |
| MBT:WL/NT | 4 | No concerns | Some concerns | No concerns | Major concerns | No concerns | No concerns | Low |
| PT:WL/NT | 1 | No concerns | Some concerns | No concerns | Major concerns | No concerns | No concerns | Low |
| TAU:TGCT | 1 | Some concerns | Some concerns | No concerns | Major concerns | No concerns | No concerns | Low |
| TAU:WL/NT | 1 | Some concerns | Some concerns | No concerns | Major concerns | No concerns | No concerns | Low |
| BRI:CBT | 0 | Some concerns | Some concerns | No concerns | Major concerns | No concerns | No concerns | Low |
| BRI:EMDR | 0 | Some concerns | Some concerns | No concerns | Major concerns | No concerns | No concerns | Low |
| BRI:ET | 0 | Some concerns | Some concerns | No concerns | Major concerns | No concerns | No concerns | Low |
| BRI:MBT | 0 | Some concerns | Some concerns | No concerns | Major concerns | No concerns | No concerns | Low |
| BRI:PS | 0 | Some concerns | Some concerns | No concerns | Major concerns | No concerns | No concerns | Low |
| BRI:PT | 0 | No concerns | Some concerns | No concerns | Major concerns | No concerns | No concerns | Low |
| BRI:TAU | 0 | Some concerns | Some concerns | No concerns | Major concerns | No concerns | No concerns | Low |
| BRI:TGCT | 0 | Some concerns | Some concerns | No concerns | Major concerns | No concerns | No concerns | Low |
| CBT:MBT | 0 | No concerns | Some concerns | No concerns | Major concerns | No concerns | No concerns | Low |
| CBT:PT | 0 | No concerns | Some concerns | No concerns | Major concerns | No concerns | No concerns | Low |
| CBT:TGCT | 0 | Some concerns | Some concerns | No concerns | Major concerns | No concerns | No concerns | Low |
| EMDR:ET | 0 | No concerns | Some concerns | No concerns | Major concerns | No concerns | No concerns | Low |
| EMDR:MBT | 0 | No concerns | Some concerns | No concerns | Major concerns | No concerns | No concerns | Low |
| EMDR:PS | 0 | No concerns | Some concerns | No concerns | Major concerns | No concerns | No concerns | Low |
| EMDR:PT | 0 | No concerns | Some concerns | No concerns | Major concerns | No concerns | No concerns | Low |
| EMDR:TAU | 0 | No concerns | Some concerns | No concerns | Major concerns | No concerns | No concerns | Low |
| EMDR:TGCT | 0 | Some concerns | Some concerns | No concerns | Major concerns | No concerns | No concerns | Low |
| ET:MBT | 0 | Some concerns | Some concerns | No concerns | Major concerns | No concerns | No concerns | Low |
| ET:PS | 0 | No concerns | Some concerns | No concerns | Major concerns | No concerns | No concerns | Low |
| ET:PT | 0 | No concerns | Some concerns | No concerns | Major concerns | No concerns | No concerns | Low |
| ET:TGCT | 0 | Some concerns | Some concerns | No concerns | Major concerns | No concerns | No concerns | Low |
| MBT:PS | 0 | No concerns | Some concerns | No concerns | Major concerns | No concerns | No concerns | Low |
| MBT:PT | 0 | No concerns | Some concerns | No concerns | Major concerns | No concerns | No concerns | Low |
| MBT:TAU | 0 | Some concerns | Some concerns | No concerns | Major concerns | No concerns | No concerns | Low |
| MBT:TGCT | 0 | Some concerns | Some concerns | No concerns | Major concerns | No concerns | No concerns | Low |
| PS:PT | 0 | No concerns | Some concerns | No concerns | Major concerns | No concerns | No concerns | Low |
| PS:TAU | 0 | No concerns | Some concerns | No concerns | Major concerns | No concerns | No concerns | Low |
| PS:TGCT | 0 | Some concerns | Some concerns | No concerns | Major concerns | No concerns | No concerns | Low |
| PS:WL/NT | 0 | No concerns | Some concerns | No concerns | Major concerns | No concerns | No concerns | Low |
| PT:TAU | 0 | No concerns | Some concerns | No concerns | Major concerns | No concerns | No concerns | Low |
| PT:TGCT | 0 | Some concerns | Some concerns | No concerns | Major concerns | No concerns | No concerns | Low |
| TGCT:WL/NT | 0 | Some concerns | Some concerns | No concerns | Major concerns | No concerns | No concerns | Low |
